# Synthesis and Evaluation of Anticancer Activities of Novel C-28 Guanidine-Functionalized Triterpene Acid Derivatives

**DOI:** 10.3390/molecules23113000

**Published:** 2018-11-16

**Authors:** Anna Spivak, Rezeda Khalitova, Darya Nedopekina, Lilya Dzhemileva, Milyausha Yunusbaeva, Victor Odinokov, Vladimir D’yakonov, Usein Dzhemilev

**Affiliations:** Institute of Petrochemistry and Catalysis, Russian Academy of Sciences, 141 Prospekt Oktyabrya, Ufa 450075, Russia; rezedamuf@yandex.ru (R.K.); dashana25@gmail.com (D.N.); milyausha_ufa@mail.ru (M.Y.); odinokov@anrb.ru (V.O.); DyakonovVA@gmail.com (V.D.); dzhemilev@anrb.ru (U.D.)

**Keywords:** triterpenoids, betulinic acid, ursolic acid, oleanolic acid, amino group, guanidine group, cytotoxicity, apoptosis, cell cycle

## Abstract

Triterpene acids, namely, 20,29-dihydrobetulinic acid (BA), ursolic acid (UA) and oleanolic acid (OA) were converted into C-28-amino-functionalized triterpenoids **4**–**7**, **8a**, **15**, **18** and **20**. These compounds served as precursors for the synthesis of novel guanidine-functionalized triterpene acid derivatives **9b**–**12b**, **15c**, **18c** and **20c**. The influence of the guanidine group on the antitumor properties of triterpenoids was investigated. The cytotoxicity was tested on five human tumor cell lines (Jurkat, K562, U937, HEK, and Hela), and compared with the tests on normal human fibroblasts. The antitumor activities of the most tested guanidine derivatives was lower, than that of corresponding amines, but triterpenoids with the guanidine group were less toxic towards human fibroblasts. The introduction of the tris(hydroxymethyl)aminomethane moiety into the molecules of triterpene acids markedly enhanced the cytotoxic activity of the resulting conjugates **15**, **15c**, **18b**,**c** and **20b**,**c** irrespective of the triterpene skeleton type. The dihydrobetulinic acid amine **15**, its guanidinium derivative **15c** and guanidinium derivatives of ursolic and oleanolic acids **18c** and **20c** were selected for extended biological investigations in Jurkat cells, which demonstrated that the antitumor activity of these compounds is mediated by induction of cell cycle arrest at the S-phase and apoptosis.

## 1. Introduction

**Figure 1:**
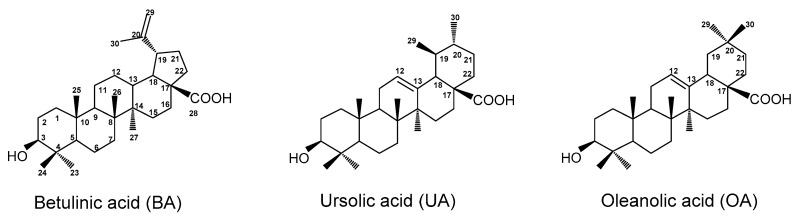
Betulinic, ursolic and oleanolic acids.

Among natural products of plant origin that are considered as abundant sources of lead structures for the discovery of new drugs, the pentacyclic lupane, ursane, and oleanane triterpenoids occupy a prominent place [1,2]. Triterpene acids (betulinic, ursolic, and oleanolic acids, Figure 1) are of interest for pharmacological research, as they exhibit a variety of biological activities including antimicrobial, antiparasitic, antitumor, and antiviral, in particular, anti-HIV, types of activity [3,4,5,6]. Among these properties of triterpenoids, of special interest is their anticancer activity and the ability to trigger the mitochondrial apoptosis pathway in various types of human cancer cells [5,6,7,8,9]. Thus, betulinic acid is capable of inducing apoptosis in tumor cells, such as melanoma, adenocarcinoma, neuroblastoma, medulloblastoma, glioblastoma and neuroectodermal tumors [7,8,9]. The *in vivo* anticancer activity of betulinic acid was identified using xenograft models [10,11]. The ursolic acid can also induce apoptosis, autophagy, and cell cycle arrest through various pathways, such as inhibition of DNA replication, stimulation of reactive oxygen species (ROS) production, and affecting the balance between proapoptotic and antiapoptotic proteins [6,12,13].

The useful pharmacological properties of triterpene acids are successfully combined with their acceptable systemic toxicity towards animals. However, the relatively low anticancer potential and high hydrophobicity of these secondary metabolites markedly hamper their advancement as anticancer drug candidates. For this reason, active search is in progress for analogues of natural triterpenoids with a higher biological potential and enhanced pharmacological characteristics (hydrophilicity, bioavailability) [8,14,15]. It has been shown [16,17,18,19,20,21,22,23,24,25] that conversion of triterpene compounds to cationic derivatives such as quaternary ammonium [16,17], pyridinium [18,19] or triphenylphosphonium salts [20,21,22,23,24,25] may serve as an efficient approach to improving bioavailability and selectivity of their biological action. Our recent study has shown that triphenylphosphonium derivatives of betulinic and ursolic acids are substantially superior over their prototypes in the antitumor activity and in the triggering mitochondria-dependent apoptosis of cancer cells [24,25].

However, the cytotoxic activity of the phosphonium salts was comparable with their cytotoxic activity against normal peripheral blood cells. In continuation of the search for efficient and selective antitumor agents, we have investigated novel cationic derivatives of pentacyclic triterpenoids containing guanidine groups, which are readily protonated at a physiological pH level. The introduction of hydrophilic guanidine groups into hydrophobic triterpene acid molecules may enhance their transmembrane transport and physicochemical characteristics. Meanwhile, the new hybrid molecules may preserve the selectivity of cytotoxic action against normal cells inherent in the natural triterpene acids. The guanidine group is a common key unit in various natural and synthetic compounds demonstrating antimicrobial, antiviral, and antitumor activities [26]. High symmetry of the Y-shaped guanidinium group promotes the formation of two parallel hydrogen bonds with the biologically relevant counterparts. Unlike ammonium groups, in which the charge is localized on one nitrogen atom (hard cations), guanidinium groups with a delocalized charge actively interact through hydrogen bonds with soft ions such as phosphates and sulfates. This feature of the guanidinium cation induce the efficient transport of biologically active substances through liposomal and cell membranes [27,28,29]. Furthermore, because of high basicity (pKa 13.5), the guanidinium group is important for selective delivery of cytotoxic molecules to tumor cells. Guanidine derivatives can be accumulated in the mitochondria of tumor cells, thus destroying the mitochondrial potential and inhibiting the mitochondrial respiratory chain [29,30].

Polyamines, which are precursors of aminoalkylguanidines, are also used to develop chemotherapeutic agents, including antibacterial and antitumor compounds [31,32]. Structurally, polyamine molecules contain positively charged nitrogen atoms at physiological pH value and can serve as electrostatic bridges between negatively charged phosphates. They are able to bind to negatively charged DNA macromolecules. However, some of physiological diamines, polyamines, and their synthetic analogues have exhibited high toxicity toward normal cells. A large body of data has now been accumulated on the biological activity of polyaminosterols, among which squalamine, trodusquemine, and their synthetic analogues are best known [33,34,35,36]. The synthesis and biological properties of polyamino triterpene acids are described in several publications [6,37,38,39,40]; the effect of introduction of the guanidine group into triterpenoid molecules has not been studied so far. Here we describe the synthesis and comparative evaluation of the cytotoxic and apoptosis-inducing activities of new guanidine derivatives of pentacyclic lupane, ursane, and oleanane triterpenoids and their precursors—C-28 conjugates of triterpene acids with some linear and branched mono-, di-, and triaminoalkanes.

## 2. Results and Discussion

### 2.1. Chemistry

**Scheme 1:**
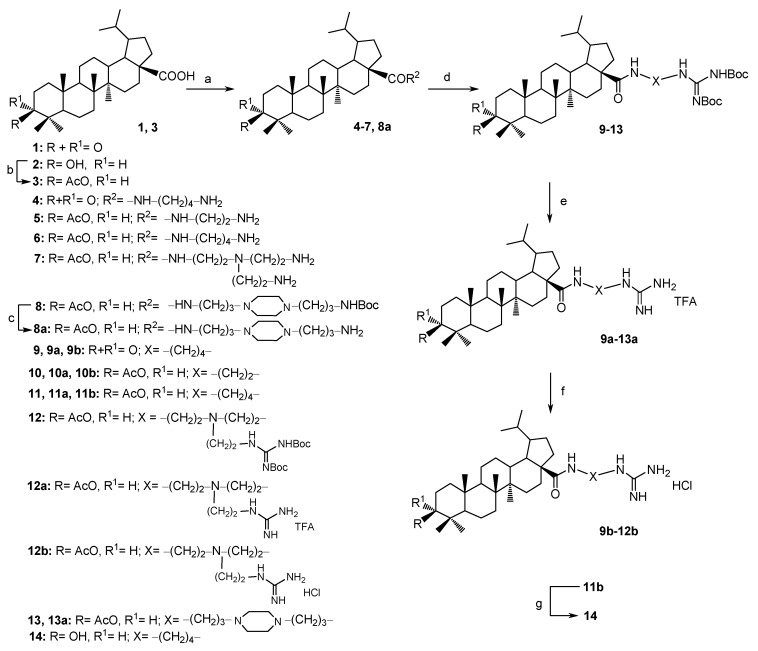
Synthesis of target compounds **9b**–**12b** and **14**. Reagents and conditions: (**a**) 1. (COCl)_2_, CH_2_Cl_2_; 2. 1,2-diaminoethane or 1,4-diaminobutane or tris(2-aminoethyl)amine or *N*-*tert*-butoxy-carbonyl-1,4-bis(3-aminopropyl)piperazine, Et_3_N, CH_2_Cl_2_, r.t.; (**b**) AcCl, THF, Py, DMAP, r.t.; (**c**) 10% TFA, CH_2_Cl_2_, 3 h, r.t.; (**d**) 1,3-di-Boc-2-(trifluoromethylsulfonyl)guanidine, Et_3_N, CH_2_Cl_2_; (**e**) 50% TFA, CH_2_Cl_2_, 2–4 h, r.t.; (**f**) 5 M HCl, MeOH; (**g**) 4 N NaOH, MeOH, THF.

While synthesizing the target compounds, we found that the Boc-deprotection of guanidine derivatives of betulinic and betulonic acids in acid medium (50% TFA in CH_2_Cl_2_) is complicated by skeletal rearrangements of the lupane skeleton. It is known from the literature [41,42] that hydrogenation of the C-20 double bond of lupane triterpenoids does not considerably affect their cytotoxic activity and selectivity between normal and tumor cell lines; therefore, in the subsequent experiments, we used 20,29-dihydrobetulinic and 20,29-dihydrobetulonic acids **1** and **2** to prepare target compounds **9b**–**12b** and **14** (Scheme 1).

Acetate **3** obtained upon hydroxyl group protection in dihydrobetulinic acid **2**, and dihydrobetulonic acid **1** were converted to C-28 amide derivatives **4**–**7** and **8a** via relatively unstable acid chlorides (Scheme 1).

**Scheme 2:**
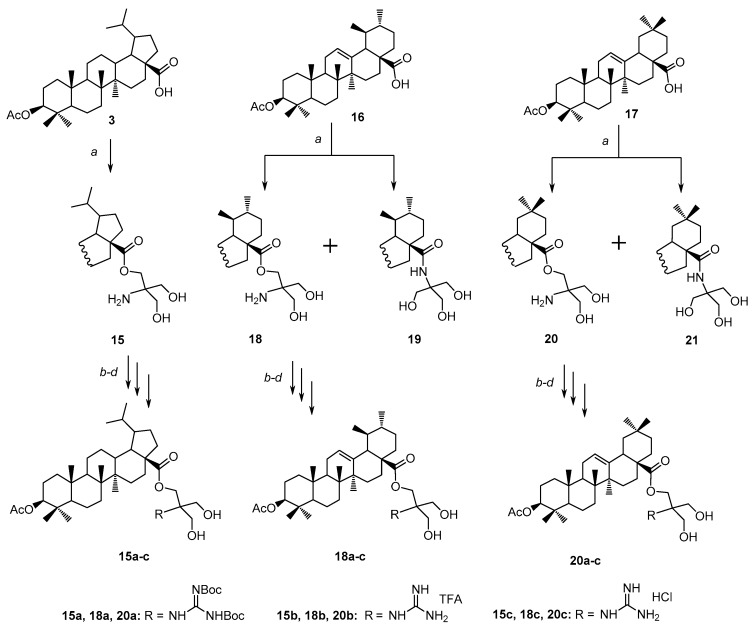
Synthesis of compounds **15a**–**c**, **18a**–**c**, **20a**–**c**. *Reagents and conditions*: (**a**) 1. (COCl)_2_, CH_2_Cl_2_; 2. tris(hydroxymethyl)aminomethane, DMAP, Py, CH_2_Cl_2_; (**b**) 1,3-di-Boc-2-(trifluoromethyl-sulfonyl)guanidine, Et_3_N, CHCl_3_, reflux; (**c**) 50% TFA, CH_2_Cl_2_, 2–4 h, r.t.; (**d**) 5M HCl, MeOH.

The reactions were carried out with 1,2-diaminoethane, 1,4-diaminobutane (putrescine), tris(2-aminoethyl)amine, and *N*-*tert*-butyloxycarbonyl-1,4-bis(3-aminopropyl)piperazine. The use of branched triamine resulted in two guanidine functions being introduced into the triterpenoid molecule. Amides **4**–**7** were synthesized using a 3-fold molar excess of amines over triterpenes in order to avoid the formation of dimeric products. Compound **8a** was formed in a good yield only with the use of *N*-Boc bis-aminopropylpiperazine, which was synthesized as described in reference [43]. The *N*-Boc protection was then removed by treatment with 10% trifluoroacetic acid in CH_2_Cl_2_. Guanylation of amines **4**–**7** and **8a** was carried out according to the typical procedure [44] by using commercially available *N*,*N*′-di-Boc-*N*″-triflylguanidine. The expected compounds **9**–**13** were obtained in 60–88% yields after column chromatography on SiO_2_. The subsequent treatment of Boc derivatives **9**–**13** with CF_3_COOH and then with HCl/MeOH gave hydrochlorides **9b**–**13b**. Dihydrobetulinic acid guanidinium hydrochloride **14** was prepared by saponification of the 3-OAc function in conjugate **11b**. A well studied and promising betulinic acid derivative is 2-amino-3-hydroxy-2-(hydroxymethyl)propyl 3-*O*-acetyl betulinate, known as the anticancer agent NVX-207 [45,46,47,48]. We have synthesized its C-20,29 hydrogenated derivative **15**. Ursolic and oleanolic acetates **16** and **17** were converted into NVX-207 analogues **18** and **20** with ursane and oleanane skeletons (Scheme 2).

The reactions under reported conditions [45] afforded the desired esters **15**, **18**, and **20** containing the terminal NH_2_ group in the branched C-28 chain and amides **19** and **21** in 15–23% and 24–32% yields, respectively. Guanylation of the free amino groups in the resulting amines **15**, **18**, and **20** on treatment with *N*,*N*′-di-Boc-*N*″-triflylguanidine gave rise to guanidine derivatives **15a**, **18a**, and **20a** in 63–79% yields. The Boc-deprotection with 50% TFA/CH_2_Cl_2_ and the subsequent treatment of salts **15b**, **18b**, and **20b** with 5M HCl in MeOH yielded hydrochlorides **15c**, **18c**, and **20c**.

The structures of all products were confirmed by 1D (^1^H, ^13^C, ^19^F) and 2D (HSQC, HMBC, COSY) NMR experiments and MALDI TOF MS. The ^13^C-NMR spectra of compounds **4**–**7**, **8a**, **15**, **18**, and **20** show signals for amide carbon atoms at 176.3–179.3 ppm, while the amide group protons resonate at 5.86–6.79 ppm in the ^1^H-NMR spectra. In the spectra of guanidine derivatives **9**–**13**, **15a**, **18a**, and **20a**, the NH-C=N and NH-Boc proton signals occurred at 8.30–9.06 ppm and 11.47–11.53 ppm, respectively. The signals for the guanidine group carbon atoms were observed at 161.8–163.6 ppm.

### 2.2. Biological Evaluation

The *in vitro* cytotoxic activity of triterpene acids (dihydrobetulinic, ursolic and oleanolic acids), twelve guanidinium salts, and some of their precursors, primary amines **4**–**6**, **8a**, and **15**, was evaluated on five human tumor cell lines: Jurkat (T-lymphoblastic leukemia), K562 (chronic myeloid leukemia), U937 (histiocytic lymphoma), HEK 293 (embryonic kidney), and HeLa (cervical cancer). The possible cell toxicity was assessed against normal human fibroblasts. Most of the tested compounds showed moderate or significant activity against Jurkat, K562, and U937 cells as compared to triterpenoic acids (Table 1).

Amino derivatives **4**–**6** and **8a** showed cytotoxic activity against all tumor cell cultures with IC_50_ values of 1.3–8 µM. However, these compounds were also cytotoxic against fibroblasts. Contrary to our expectations, guanylation of the terminal amino groups of lupane triterpenoids with C-28 linear aminoalkane chains (compounds **9b**–**11b**, **13a**, and **14**) or the branched tris-aminoethyl moiety (compounds **12a**,**b**) did not considerably enhance the cytotoxic action. Of the listed guanidinium salts, only compounds **9b**, **11b**, and **14** showed an approximately 3-fold increase in the cytotoxic activity against Jurkat cells in comparison with dihydrobetulinic acid **2** and also showed selectivity towards Jurkat cells with an SI of 8.5, 7.3, and 5.6 respectively (SI = IC_50_ fibroblasts/IC_50_ Jurkat cells). The introduction of the tris(hydroxymethyl)aminomethane moiety into the molecules of triterpene acids **3**, **16** and **17** markedly enhanced the cytotoxic activities for the resulting conjugates **15**, **15c**, **18b**,**c**, and **20b**,**c**, irrespective of the triterpene skeleton type. The C-28 esters of dihydrobetulinic, ursolic, and oleanolic acids with amino and guanidine groups in the ester side chain had from moderate to good activity against Jurkat and K562 cell lines. For example, the IC_50_ values of compounds **15c** and **18c** were 3.1 and 3.8 µM for T-lymphoblastic leukemia cells and 2.3 and 11.0 µM for chronic myeloid leukemia cells. The most pronounced differences in the antitumor activity were found for oleanolic acid and its conjugates **20b**,**c**. Indeed, the IC_50_ values of oleanolic acid, **20b**, and **20c** for Jurkat cells were 271, 6.7, and 7.6 µM, respectively. It is worth noting that amine **15**, its guanidine derivative **15c**, and guanidinium salts of ursolic and oleanolic acids **18b**,**c** and **20b**,**c** showed acceptable selectivity towards Jurkat tumor cell with an SI from 6.9 to 13.4.

**Figure 2:**
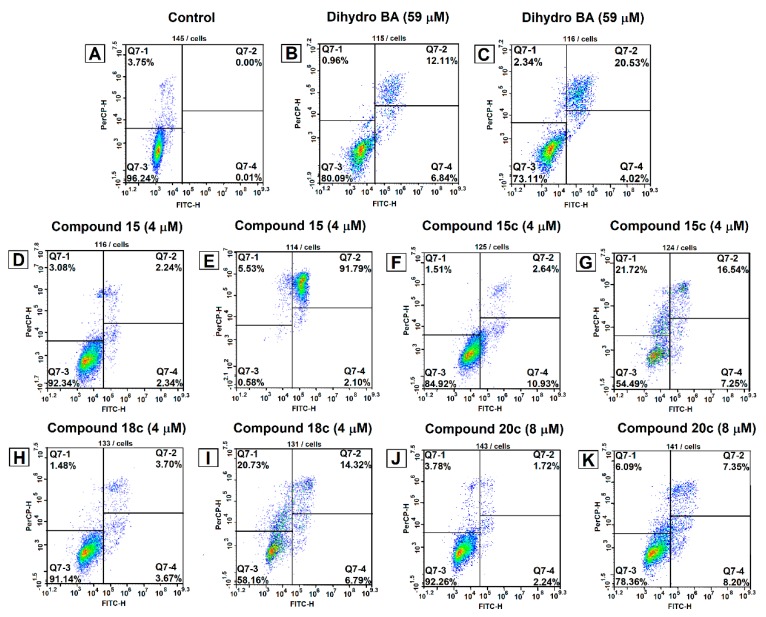
AnnexinV/7-AAD staining upon induction of apoptosis in Jurkat cells. Cells were treated with compounds at their IC_50_ concentration for 24 and 48 h. Then, the cells were harvested, stained with Annexin V/7-AAD and analyzed by flow cytometry. The experiments were performed three times, and the results of the representative experiments are shown. The first cytogram represents an untreated cell sample (**A**); after incubation with dihydrobetulinic acid for 24 h (**B**) and for 48 h (**C**); after incubation with compound **15** for 24 h (**D**) and for 48 h (**E**); after incubation with compound **15c** for 24 h (**F**) and for 48 h (**G**); after incubation with compound **18c** for 24 h (**H**) and for 48 h (**I**); after incubation with compound **20c** for 24 h (**J**) and for 48 h (**K**). Q7-1, necrotic cells; Q7-2, late apoptotic cells; Q7-3, living cells; Q7-4, early apoptotic cells.

The identified lead compounds with the highest cytotoxicity characteristics, **15**, **15c**, **18c**, and **20c**, were further evaluated for the possible apoptosis induction in tumor cell cultures. The measurements were done by flow cytometry (Figure 2).

The highest percentage of late apoptosis (91.7%) was detected upon the treatment of Jurkat cells with the test compound **15** at IC_50_ concentration exposure for 48 h as depicted in Figure 2E. Compounds **15c**, **18c** and **20c** also showed apoptotic mode of cell death on Jurkat cells line, but, in this case, the apoptotic effect of these guanidine derivatives was notably weaker. Thus, after treatment of Jurkat cells with compound **15c** at IC_50_ concentration (4 μM) the number of vital cells is decreased from 96.2% (control) to 54.5%. Total number of apoptotic cells population constituted 23.7% (7.2% and 16.5% of early and late apoptotic cells, respectively) and number of necrotic cells was 21.7%. Comparable results were obtained with the guanidine derivative of ursolic acid **18c** (6.8, 14.3, and 20.7% of early, late apoptotic cells and necrotic cells, respectively). Treatment of the Jurkat cells with **20c** resulted in about 15.5% apoptotic cells and 6.1% necrotic cells, with 78.4% of the cells still being considered vital (Figure 2K). Next, we analyzed the ability of dihydrobetulinic acid to stimulate apoptosis. Our results showed that dihydrobetulinic acid triggers apoptosis in Jurkat cells at higher doses as compared to derivatives **15**, **15c**, and **18c**. The number of apoptotic cells on treatment with dihydrobetulinic acid (59 μM) for 48 h constituted around 24% (4.0% of early-stage and 20.5% of secondary necrotic/late-stage apoptotic), while the number of vital cells was 73.1% (Figure 2C).

In summary, our results indicate that the apoptosis is induced in Jurkat cells by all test compounds. However, we observed a higher rate of necrosis after **15c**, **18c** and **20c** incubation compared to derivative **15**.

**Figure 3:**
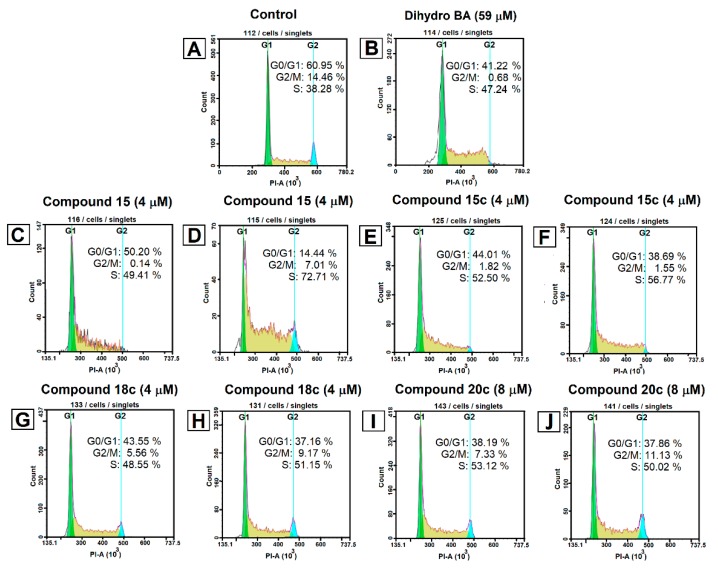
Cell cycle analysis in Jurkat cells. Jurkat cells treated with tested compounds at the IC_50_ concentration for 24 and 48 h. The cells were trypsinized, harvested and washed three times with ice-PBS for PI-stained DNA content detected by flow cytometry. The experiments were performed three times, and the results of the representative experiments are shown. The first cytogram represents an untreated cell sample (**A**); after incubation with dihydrobetulinic acid for 48 h (**B**); after incubation with compound **15** for 24 h (**C**) and for 48 h (**D**); after incubation with compound **15c** for 24 h (**E**) and for 48 h (**F**); after incubation with compound **18c** for 24 h (**G**) and for 48 h (**H**); after incubation with compound **20c** for 24 h (**I**) and for 48 h (**J**).

DNA flow cytometry was also used to analyze the cell cycle kinetics in Jurkat cells pre-incubated with dihydrobetulinic acid and derivatives **15**, **15c**, **18c**, or **20c** at their IC_50_ concentration for 24 and 48 h (Figure 3).

The distribution of DNA content in Jurkat cells reveals whether cell proliferation is arrested at one checkpoint. The results of all experiments showed in significant S-phase arrest in cells after treating with these compounds. Thus, the ratio of cells in the S phase increased from 38.3% (control) to 72.7% in cells treated with **15** (4 μM for 48 h) and increased to 47.2–56.8% in cells treated with dihidrobetulinic acid, **15c**, **18c**, or **20c**. The appropriate decrease of the number of cells in the G2/M phase was observed. For example, the treatment of Jurkat cells with **15** and **15c** resulted in a decrease of cells in the G2/M phase from 14.5% (control) to 7.0 and 1.5% (Figure 3D,F). Considering these results, we assume that dihydrobetulinic acid and compounds **15**, **15c**, **18c**, or **20c** are able to trigger programmed cell death, including apoptotic mechanisms and cell cycle arrest in the S-phase.

## 3. Materials and Methods

### 3.1. Chemistry

IR spectra (thin films or solutions in CHCl_3_) were obtained with use of a Vertex 70v spectrometer (Bruker, Karlsruhe, Germany). ^1^H- and ^13^C-NMR spectra were recorded in CDCl_3_, in MeOD or in *d*_6_-DMSO with Me_4_Si as the internal standard on an AVANCE–500 instrument (500.13 (^1^H), 125.78 MHz (^13^C), 470.59 MHz (^19^F)) or on an AVANCE-400 (400.13 (^1^H), 100.62 MHz (^13^C), 376.50 MHz (^19^F)) (Bruker). Mass spectra of new compounds were recorded on a Bruker–Autoflex III spectrometer (MALDI TOF, positive ion mode, sinapic acid as the matrices). Optical rotation was determined on a 141 polarimeter (Perkin–Elmer, Beaconsfield, UK). Specific rotation [α]_D_ is expressed in (deg·mL)/(g·dm)^−1^; the concentration of the solution *c* is expressed in g/100 mL. Elemental analysis was carried out on a 1106 analyzer (Carlo Erba, Milan, Italy). TLC was carried out on Sorbfil plates (Sorbpolimer, Krasnodar, Russia) in hexane–EtOAc and chloroform–methanol, spots were visualized with anisaldehyde. Silica gel L (KSKG grade, 50–160 μm) was employed for column chromatography. All reagents and solvents were of the purest grade available, and generally were used without further treatment. The starting compounds ursolic, oleanolic acids and reagents: sodium borohydride (NaBH_4_), acetyl chloride, 10% Pd/C, oxalyl chloride, 1,2-diaminoethane, 1,4-diaminobutane, tris(2-aminoethyl)amine, 1,4-bis(3-aminopropyl)piperazine, tris(hydroxymethyl)aminomethane, 1,3-di-Boc-2-(trifluoromethylsulfonyl)guanidine, triethylamine (Et_3_N), dimethylaminopyridine (DMAP), trifluoroacetic acid (TFA) were purchased from Acros Organics (Geel, Belgium). Dihydrobetulonic and dihydrobetulinic acids were obtained from betulin according to the known procedures [41]. Acetates of oleanolic, ursolic, dihydrobetulonic and dihydrobetulinic acids were synthesized according to the typical procedures. Mono-Boc-protected bis-aminopropylpiperazine and compounds **19**–**23** were prepared by a reported methods [43,45]. NMR ^1^H and ^13^C spectra of all new compounds are in Supplementary Materials.

#### 3.1.1. General Procedure for the Synthesis of Amines **4**–**7**

Oxalyl chloride (0.13 mL, 1.5 mmol) was added with stirring to a solution of compounds **1** or **3** (0.5 mmol) in dry CH_2_Cl_2_ (5 mL) precooled to 0 °C, and stirring of the reaction mixture was continued at room temperature for 2 h. Then the solvent and excess oxalyl chloride were removed under vacuum. The amine (1.5 mmol) was dissolved in dry CH_2_Cl_2_ (2 mL) and under vigorous stirring were added Et_3_N (0.2 mL, 1.5 mmol) and a solution of the acid chloride of **1** or **3** (0.5 mmol) in dry CH_2_Cl_2_ (4 mL). The mixture was stirred for 24 h (monitoring by TLC). The mixture was then poured into cold H_2_O and extracted with CH_2_Cl_2_ (2 × 15 mL each). The organic phases washed brine and were dried over Na_2_SO_4_ and evaporated under reduced pressure. The residue was chromatographed on silica gel, using CH_2_Cl_2_/MeOH 30:1→1:1 (*v*/*v*), to obtain pure compounds **4**–**7**.

*N-(4-Aminobuthyl)-3-oxolupane-28-amide* (**4**), White powder, 78% yield; mp 170–172 °C (EtOH); ${\left[\alpha \right]}_{D}^{19}$: +4° (*c* 0.23, CH_2_Cl_2_); IR (CHCl_3_) ν_max_ 1641, 1702 (C=O), 3357 (NH) cm^−1^; ^1^H-NMR (500 MHz, CDCl_3_) *δ*: 5.86 (t, *J* = 5.5 Hz, 1H, CONH), 3.32–3.20 (m, 2H, H-1′), 2.76 (t, *J* = 6.5 Hz, 2H, H-4′), 2.55–1.14 (m, 26H, CH, CH_2_ in pentacyclic skeleton, 4H, H-2′, H-3′), 1.08, 1.03, 0.98, 0.97, 0.94 (all s, 3H each, H-23–H-27), 0.87 (d, *J* = 6.5 Hz, 3H, H-29), 0.75 (d, *J* = 7.0 Hz, 3H, H-30); ^13^C-NMR (125 MHz, CDCl_3_) *δ*: 218.2 (C-3), 176.3 (C-28), 56.0 (C-17), 55.0 (C-5), 49.8 (C-9), 49.5 (C-19), 47.4 (C-4), 44.2 (C-18), 42.7 (C-14), 41.6 (C-4′), 40.7 (C-8), 39.6 (C-1), 39 (C-22), 38.8 (C-1′), 37.7 (C-13), 36.9 (C-10), 34.2 (C-2), 33.8 (C-16), 33.6 (C-7), 30.6 (C-3′), 29.9 (C-20), 29.4 (C-15), 27.2 (C-23), 27.0 (C-2’), 26.6 (C-12), 23.1 (C-29), 23.0 (C-21), 21.5 (C-24), 21.1 (C-11), 19.7 (C-6), 16.0 (C-25), 15.9 (C-26), 14.6 (C-30), 14.5 (C-27); Anal. Calcd. for C_34_H_58_N_2_O_2_: C, 77.51, H, 11.10. Found: C, 78.03, H, 11.02%. MS: *m*/*z* 527.45 [M + H]^+^ (calcd. for C_34_H_58_N_2_O_2_, 526.45).

*3β-N-(2-Aminoethyl)-3-O-acetyl-lupane-28-amide* (**5**), White powder, 74% yield; mp 120–122 °C (EtOH); ${\left[\alpha \right]}_{D}^{19}$ −8° (*c* 0.24, CH_2_Cl_2_); IR (CHCl_3_) *ν*_max_ 1647, 1735 (C=O), 3367 (NH) cm^−1^; ^1^H-NMR (500 MHz, CDCl_3_) *δ*: 6.14 (t, *J* = 5.5 Hz, 1H, CONH), 4.48–4.44 (m, 1H, H-3), 3.35–3.21 (m, 2H, H-1′), 2.81 (t, *J* = 6.0 Hz, 2H, H-2′), 2.43–0.97 (m, 25H, CH, CH_2_ in pentacyclic skeleton), 2.01 (s, 3H, CH_3_CO–), 0.94, 0.92, 0.85, 0.84, 0.83, 0.82 (all s, 3H each, H-23–H-27 and H-29), 0.77 (d, *J* = 10.0 Hz, 1H, H-5), 0.73 (d, *J* = 7.0 Hz, 3H, H-30); ^13^C-NMR (125 MHz, CDCl_3_) *δ*: 176.7 (C-28), 171.0 (COCH_3_), 80.9 (C-3), 56.2 (C-17), 55.4 (C-5), 50.3 (C-9), 49.5 (C-19), 44.3 (C-18), 42.6 (C-14), 41.8 (C-2′), 41.7 (C-1′), 40.8 (C-8), 38.7 (C-1), 38.4 (C-22), 37.8 (C-4), 37.6 (C-13), 37.1 (C-10), 34.4 (C-7), 33.6 (C-16), 29.9 (C-20), 29.5 (C-15), 27.9 (C-23), 27.0 (C-2), 23.7 (C-12), 23.1 (C-21, C-29), 21.3 (COCH_3_), 21.0 (C-11), 18.2 (C-6), 16.5 (C-25), 16.2 (C-24, C-26), 14.6 (C-30), 14.5 (C-27); Anal. Calcd. for C_34_H_58_N_2_O_3_: C, 75.23, H, 8.84. Found: C, 75.74, H, 8.79%. MS: *m*/*z* 543.40 [M + H]^+^ (calcd. for C_34_H_58_N_2_O_3_, 542.44).

*3β-N-(4-Aminobuthyl)-3-O-acetyl-lupane-28-amide* (**6**), White powder, 58% yield; mp 104–106 °C (EtOH); ${\left[\alpha \right]}_{D}^{19}$ −11° (*c* 0.69, CHCl_3_); IR (CHCl_3_) *ν*_max_ 1646, 1734 (C=O), 3367 (NH) cm^−1^; ^1^H-NMR (500 MHz, CDCl_3_) *δ*: 5.86 (t, *J* = 5.5 Hz, 1H, CONH), 4.48–4.45 (m, 1H, H-3), 3.30–3.17 (m, 2H, H-1′), 2.73 (t, *J* = 6.5 Hz, 2H, H-4′), 2.47–0.98 (m, 25H, CH, CH_2_ in pentacyclic skeleton, 4H, H-2′, H-3′), 2.03 (s, 3H, CH_3_CO–), 0.95, 0.93, 0.92, 0.84, 0.83, 0.82, (all s, 3H each, H-23–H-27 and H-29), 0.78 (d, *J* = 10.0 Hz, 1H, H-5), 0.73 (d, *J* = 7.0 Hz, 3H, H-30); ^13^C-NMR (125 MHz, CDCl_3_) *δ*: 176.3 (C-28), 171.0 (COCH_3_), 80.9 (C-3), 56.0 (C-17), 55.4 (C-5), 50.3 (C-9), 49.5 (C-19), 44.3 (C-18), 42.6 (C-14), 41.6 (C-4′), 40.8 (C-8), 39.0 (C-1′), 38.7 (C-22), 38.4 (C-1), 37.8 (C-4), 37.6 (C-13), 37.1 (C-10), 34.4 (C-7), 33.6 (C-16), 30.7 (C-3′), 29.9 (C-20), 29.4 (C-15), 27.9 (C-23), 27.2 (C-2′), 27.0 (C-2), 23.7 (C-12), 23.0 (C-21, C-29), 21.3 (COCH_3_), 21.0 (C-11), 18.2 (C-6), 16.5 (C-25), 16.2 (C-26, C-24), 14.6 (C-30), 14.5 (C-27); Anal. Calcd. for C_36_H_62_N_2_O_3_: C, 75.74, H, 10.95. Found: C, 76.01, H, 10.89%. MS: *m*/*z* 609.43 [M + K]^+^ (calcd. for C_36_H_62_N_2_O_3_, 570.48).

*3β-N-[2-(N,N*′*-bis-Aminoethyl)-aminoethyl]-3-O-acetyl-lupane-28-amide* (**7**), White powder, 69% yield; mp 108–110 °C (EtOH); ${\left[\alpha \right]}_{D}^{19}$ −5° (*c* 0.16, C_2_H_5_OH); IR (CHCl_3_) *ν*_max_ 1648, 1735 (C=O), 3441 (NH) cm^−1^; ^1^H-NMR (400 MHz, MeOD) *δ*: 4.49–4.45 (m, 1H, H-3), 3.33–3.27 (m, 2H, H-1′), 2.74–2.72 (m, 4H, H-4′, H-42″), 2.60–2.58 (m, 6H, H-2′, H-3′, H-3″), 2.32–0.84 (m, 26H, CH, CH_2_ in pentacyclic skeleton), 2.05 (s, 3H, CH_3_CO–), 1.02, 1.00, 0.93, 0.90, 0.89, 0.88 (all s, 3H each, H-23–H-27 and H-29), 0.79 (d, *J* = 6.4 Hz, 3H, H-30); ^13^C-NMR (100 MHz, MeOD) *δ*: 179.3 (C-28), 172.8 (COCH_3_), 82.5 (C-3), 57.7 (C-17), 57.4 (C-5), 57.0 (C-3′, C-3″), 55.2 (C-2’), 51.9 (C-9), 51.1 (C-19), 45.5 (C-18), 43.8 (C-14), 42.2 (C-8), 40.2 (C-22), 39.9 (C-1), 39.8 (C-4′, C-4″), 39.0 (C-4), 38.8 (C-1′), 38.4 (C-13), 38.3 (C-10), 35.8 (C-7), 34.1 (C-16), 31.4 (C-20), 30.9 (C-15), 28.7 (C-23), 28.5 (C-2), 24.8 (C-12), 24.2 (C-29), 23.8 (C-21), 22.4 (COCH_3_), 21.4 (C-11), 19.4 (C-6), 17.2 (C-25), 17.0 (C-24, C-26), 15.3 (C-30, C-27); Anal. Calcd. for C_38_H_68_N_4_O_3_: C, 72.56, H, 10.90. Found: C, 73.04, H, 10.87%. MS: *m*/*z* 629.56 [M + H]^+^ (calcd. for C_38_H_68_N_4_O_3_, 628.53).

*3β-N-{[3-(3-Aminopropyl)piperazinyl]propyl}-3-O-acetyl-lupane-28-amide* (**8a**), The acid chloride of **3** was synthesized according to the general procedure for synthesis of amines **4**–**7**. Then the *N*-*tert*-butoxycarbonyl-1,4-bis(3-aminopropyl)piperazine (0.45 g, 1.5 mmol) and Et_3_N (0.25 mL, 1.8 mmol) were added to acid chloride of 3 (0.50 g, 1 mmol) in dry CH_2_Cl_2_ (10 mL). The mixture was stirred at room temperature for 16 h (monitoring by TLC), then was poured into cold H_2_O and extracted with CH_2_Cl_2_ (2 × 15 mL). The organic phases washed brine, dried over Na_2_SO_4_ and evaporated under reduced pressure to obtain compound 8. Then the compound 8 was dissolved in CH_2_Cl_2_ (7 mL), acidified with TFA 10% (*v*/*v*) in CH_2_Cl_2_ (17 mL) for deprotection reaction and stirred for around 5 h. The reaction was quenched using saturated aqueous potassium carbonate solution (20 mL). The aqueous phase was extracted with CH_2_Cl_2_ (2 × 15 mL). The organic phases washed brine, dried over Na_2_SO_4_ and evaporated under reduced pressure. The residue was chromatographed on silica gel, using CH_2_Cl_2_/MeOH 50:1→5:1, to obtain pure compound 8a. White powder, 78% yield; mp 119–121 °C (EtOH); ${\left[\alpha \right]}_{D}^{21}$ −7.4° (*c* 0.5, CHCl_3_); IR (CHCl_3_) *ν*_max_ 1735 (C=O), 3361 (NH) cm^−1^; ^1^H-NMR (500 MHz, CDCl_3_) *δ*: 6.79 (t, *J* = 5.5 Hz, 1H, CONH), 4.47–4.44 (m, 1H, H-3), 3.31–3.25 (m, 2H, H-1′), 2.78–2.77 (m, 2H, H-1″), 2.50–2.42 (m, 12H, H-3′–H-5′, H-3″–H-5″), 2.41–0.98 (m, 25H, CH, CH_2_ in pentacyclic skeleton, 4H, H-2′, H-2″), 2.02 (s, 3H, CH_3_CO–), 0.93, 0.92, 0.91, 0.83, 0.82, 0.81 (3H each, all s, H-23–H-27 and H-29), 0.77 (d, *J* = 10.5 Hz, 1H, H-5), 0.72 (d, *J* = 6.5 Hz, 3H, H-30); ^13^C-NMR (125 MHz, CDCl_3_) *δ*: 176.3 (C-28), 171.0 (COCH_3_), 80.9 (C-3), 57.8 (C-3′), 56.7 (C-3″), 55.9 (C-17), 55.4 (C-5), 53.5 (C-5′, C-5″), 53.3 (C-4′), 53.3 (C-4″), 50.3 (C-9), 49.6 (C-19), 44.1 (C-18), 42.6 (C-14), 40.9 (C-1″), 40.8 (C-8), 39.3 (C-1′), 38.8 (C-22), 38.4 (C-1), 37.8 (C-4), 37.4 (C-13), 37.1 (C-10), 34.4 (C-7), 33.7 (C-16), 29.9 (C-20), 29.8 (C-2′), 29.7 (C-15), 27.9 (C-23), 26.9 (C-2), 25.3 (C-2″), 23.7 (C-12), 23.0 (C-21, C-29), 21.3 (COCH_3_), 21.0 (C-11), 18.2 (C-6), 16.5 (C-25), 16.3 (C-24), 16.2 (C-26), 14.6 (C-30), 14.5 (C-27); Anal. Calcd. for C_42_H_74_N_4_O_3_: C, 73.85, H, 10.92. Found: C, 74.13, H, 10.84%. MS: *m*/*z* 683.52 [M + H]^+^ (calcd. for C_42_H_74_N_4_O_3_, 682.58).

#### 3.1.2. General Procedure for the Synthesis of Compounds **15**, **18**–**21**

Acid chlorides of **3**, **16**, **17** was synthesized according to the general procedure for synthesis of amines **4**–**7**. Then the acid chlorides of **3**, **16**, **17** (1 mmol) was dissolved in a mixture of pyridine (4 mL), CH_2_Cl_2_ (1 mL) and DMAP (0.09 g, 0.7 mmol) was added. After complete dissolution of DMAP, a solution containing of TRIS (tris(hydroxymethyl)aminomethane) (0.24 g, 2 mmol) in pyridine (0,5 mL) was added. The mixture was stirred for 10 h at room temperature and the solvent was removed rapidly under vacuum. The residue was chromatographed on silica gel, using CH_2_Cl_2_/MeOH 30:1→1:1, to obtain pure compounds **15**, **18**–**21**.

*3β-[2-Amino-3-hydroxy-2-(hydroxymethyl)propyl]-3-O-acetyl-lupane-28-oate* (**15**), White powder, 16% yield; mp 134–136 °C (EtOH); ${\left[\alpha \right]}_{D}^{21}$ −13.1° (*c* 0.51, CHCl_3_); IR (CHCl_3_) *ν*_max_ 1731 (C=O), 3366 (OH), 3446 (NH) cm^−1^; ^1^H-NMR (400 MHz, CDCl_3_) *δ*: 4.49–4.45 (m, 1H, H-3), 4.09, 4.03 (both d, *J* = 11.6 Hz, 1H each, H-1′), 3.52 (br s, 4H, H-3′, H-4′), 2.70 (br s, 4H, NH_2_, OH), 2.05 (s, 3H, CH_3_CO–), 2.24–0.81 (m, 26H, CH, CH_2_ in pentacyclic skeleton), 0.95, 0.91, 0.85, 0.84, 0.83, 0.82 (all s, 3H each, H-23–H-27 and H-29), 0.76 (d, *J* = 6.8 Hz, 3H, H-30); ^13^C-NMR (100 MHz, CDCl_3_) *δ*: 177.0 (C-28), 171.1 (COCH_3_), 80.9 (C-3), 64.9 (C-1′), 64.2 (C-3′, C-4′), 57.3 (C-17, C-2′), 55.4 (C-5), 50.2 (C-9), 48.8 (C-19), 44.2 (C-18), 42.6 (C-14), 40.7 (C-8), 38.4 (C-22), 38.1 (C-1), 37.8 (C-4), 37.5 (C-13), 37.1 (C-10), 34.3 (C-7), 32.0 (C-16), 29.7 (C-15, C-20), 27.9 (C-23), 26.9 (C-2), 23.7 (C-12), 23.0 (C-29), 22.8 (C-21), 21.3 (COCH_3_), 20.9 (C-11), 18.2 (C-6), 16.5 (C-25), 16.2 (C-24), 16.1 (C-26), 14.7 (C-30), 14.6 (C-27); Anal. Calcd. for C_36_H_61_NO_6_: C, 71.60, H, 10.18. Found: C, 72.09, H, 10.11%. MS: *m*/*z* 604.42 [M + H]^+^ (calcd. for C_36_H_61_NO_6_, 603.45).

*3β-[2-Amino-3-hydroxy-2-(hydroxymethyl)propyl]-3-O-acetylurs-12-en-28-oate* (**18**), White powder, 15% yield; mp 129–131 °C (EtOH); ${\left[\alpha \right]}_{D}^{18}$ +40° (*c* 0.74, CHCl_3_); IR (CHCl_3_) *ν*_max_ 1721 (C=O), 3444 (NH), 3468 (OH) cm^−1^; ^1^H-NMR (400 MHz, CDCl_3_) *δ*: 5.25 (br s, 1H, H-12), 4.51–4.47 (m, 1H, H-3), 4.02, 3.93 (both d, *J* = 12 Hz, 1H each, H-1′), 3.48 (br s, 4H, H-3′, H-4′), 2.68 (br s, 4H, NH_2_, OH), 2.19 (d, *J* = 11.2 Hz, 1H, H-18), 2.05 (s, 3H, CH_3_CO–), 1.99–0.81 (m, 22H, CH, CH_2_ in pentacyclic skeleton), 1.08, 0.96, 0.94, 0.88, 0.87, 0.86, 0.74 (all s, 3H each, H-23–H-27, H-29 and H-30); ^13^C-NMR (100 MHz, CDCl_3_) *δ*: 178.1 (C-28), 171.1 (COCH_3_), 138.3 (C-13), 125.6 (C-12), 80.9 (C-3), 65.7 (C-1′), 64.4 (C-3′, C-4′), 56.6 (C-2′), 55.3 (C-5), 53.0 (C-18), 48.6 (C-17), 47.4 (C-9), 42.1 (C-14), 39.5 (C-4, C-8), 38.9 (C-19), 38.3 (C-20), 37.7 (C-1), 37.1 (C-22), 36.8 (C-10), 32.9 (C-7), 30.6 (C-21), 28.1 (C-23), 27.9 (C-15), 24.3 (C-16, C-27), 23.5 (C-2, C-11), 21.3 (COCH_3_), 21.1 (C-30), 18.2 (C-6), 17.2 (C-29), 17.0 (C-26), 16.7 (C-24), 15.5 (C-25); Anal. Calcd. for C_36_H_59_NO_6_: C, 71.84, H, 9.88. Found: C, 72.13, H, 9.83%. MS: *m*/*z* 602.40 [M + H]^+^ (calcd. for C_36_H_59_NO_6_, 601.43).

*3β-N-[(1*′*,1*′,*1*′*-tris-Hidroxymethyl)methyl]-3-O-acetyl-ursolamide* (**19**), White powder, 24% yield; mp 203–205 °C (EtOH); ${\left[\alpha \right]}_{D}^{18}$ +26.9° (*c* 0.74, CH_3_OH); IR (CH_3_OH) *ν*_max_ 1734 (C=O), 2923, 2871, 2852 (OH), 3352 (NH) cm^−1^; ^1^H-NMR (500 MHz, MeOD) *δ*: 5.38 (br s, 1H, H-12), 4.50–4.47 (m, 1H, H-3), 3.66, 3.58 (both d, *J* = 10 Hz, 3H each, H-2′–H-4′), 2.12–0.88 (m, 23H, CH, CH_2_ in pentacyclic skeleton), 2.05 (s, 3H, CH_3_CO–), 1.18, 1.04, 0.99, 0.95, 0.93, 0.92, 0.91 (all s, 3H each, H-23–H-27, H-29 and H-30); ^13^C-NMR (125 MHz, CDCl_3_) *δ*: 178.5 (C-28), 170.7 (COCH_3_), 138.1 (C-13), 125.8 (C-12), 80.4 (C-3), 61.8 (C-1′), 60.8 (C-2′–C-4′), 55.1 (C-5), 53.6 (C-18), 48.3 (C-17), 47.4 (C-9), 42.3 (C-14), 39.5 (C-4, C-8), 38.9 (C-19), 38.4 (C-20), 37.7 (C-22, C-1), 36.8 (C-10), 33 (C-7), 30.9 (C-21), 28.3 (C-23), 27.8 (C-15), 24.5 (C-16), 23.7 (C-2), 23.4 (C-11, C-27), 21.5 (COCH_3_), 21.4 (C-30), 18.2 (C-6), 17.5 (C-29, C-26), 17.1 (C-24), 15.7 (C-25); Anal. Calcd. for C_36_H_59_NO_6_: C, 71.84, H, 9.88. Found: C, 72.09, H, 9.79%. MS: *m*/*z* 602.41 [M + H]^+^ (calcd. for C_36_H_59_NO_6_, 601.43).

*3β-[2-Amino-3-hydroxy-2(hydroxymethyl)propyl]-3-O-acetylolean-12-en-28-oate* (**20**), White powder, 23% yield; mp 141–143 °C (EtOH); ${\left[\alpha \right]}_{D}^{18}$ +49° (*c* 0.75, CHCl_3_); IR (CHCl_3_) *ν*_max_ 1618, 1727 (C=O), 3447 (NH) cm^−1^; ^1^H-NMR (500 MHz, CDCl_3_) *δ*: 5.30 (br s, 1H, H-12), 4.51–4.48 (m, 1H, H-3), 4.04, 3.99 (both d, *J* = 11.5 Hz, 1H each, H-1′), 3.50 (br s, 4H, H-3′, H-4′), 2.84 (d, *J* = 10.0 Hz, 1H, H-18), 2.68–2.60 (m, 4H, NH_2_, OH), 2.05 (s, 3H, CH_3_CO–), 2.01–0.83 (m, 22H, CH, CH_2_ in pentacyclic skeleton), 1.14, 0.94, 0.93, 0.92, 0.87, 0.86, 0.73 (all s, 3H each, H-23–H-27, H-29 and H-30); ^13^C-NMR (125 MHz, CDCl_3_) *δ*: 178.2 (C-28), 171.1 (COCH_3_), 143.7 (C-13), 122.6 (C-12), 80.9 (C-3), 65.7 (C-1′), 64.6 (C-3′, C-4′), 56.6 (C-2′), 55.3 (C-5), 47.5 (C-9), 47.2 (C-17), 45.7 (C-19), 41.8 (C-14), 41.5 (C-18), 39.3 (C-8), 38.1 (C-1), 37.7 (C-4), 36.9 (C-10), 33.8 (C-22), 33.0 (C-30), 32.8 (C-7), 32.7 (C-21), 30.7 (C-20), 28.0 (C-23), 27.6 (C-15), 25.8 (C-27), 23.6 (C-29), 23.5 (C-11), 23.4 (C-2), 23.1 (C-16), 21.3 (COCH_3_), 18.2 (C-6), 17.1 (C-26), 16.7 (C-24), 15.4 (C-25); Anal. Calcd. for C_36_H_59_NO_6_: C, 71.84, H, 9.88. Found: C, 72.04, H, 9.82%. MS: *m*/*z* 602.40 [M + H]^+^ (calcd. for C_36_H_59_NO_6_, 601.43).

*3β-N-[(1*′*,1*′*,1*′*-tris-Hydroxymethyl)methyl]-3-O-acetyl-oleanolamide* (**21**), White powder, 32% yield; mp 249–251 °C (EtOH); ${\left[\alpha \right]}_{D}^{18}$ +33.9° (*c* 0.61, CH_3_OH); IR (CH_3_OH) *ν*_max_ 1732(C=O), 2945, 2927 (OH), 3353 (NH) cm^−1^; ^1^H-NMR (500 MHz, *d*_6_-DMSO) *δ*: 6.62 (s, 1H, NH), 5.24 (br s, 1H, H-12), 4.97 (t, *J* = 5 Hz, 3H, –OH), 4.42–4.38 (m, 1H, H-3), 3.49–3.42 (m, 6H, H-2′–H-4′), 2.61 (d, *J* = 10.0 Hz, 1H, H-18), 2.00 (s, 3H, CH_3_CO–), 1.97–0.84 (m, 22H, CH, CH_2_ in pentacyclic skeleton), 1.91, 0.90, 0.88, 0.87, 0.82, 0.81, 0.78 (all s, 3H each, H-23–H-27, H-29 and H-30); ^13^C-NMR (125 MHz, *d*_6_-DMSO) *δ*: 178.3 (C-28), 170.6 (COCH_3_), 143.7 (C-13), 122.6 (C-12), 80.4 (C-3), 61.8 (C-1′), 60.8 (C-2′–C-4′), 55 (C-5), 47.4 (C-9), 46.8 (C-17), 46.6 (C-19), 42.0 (C-14), 41.9 (C-18), 39.5 (C-8), 38.2 (C-1), 37.7 (C-4), 36.9 (C-10), 34.1 (C-22), 33.3 (C-7), 33.2 (C-30), 32.7 (C-21), 30.8 (C-20), 28.2 (C-23), 27.4 (C-15), 25.8 (C-27), 23.8 (C-29), 23.7 (C-2), 23.4 (C-11, C-16), 21.4 (COCH_3_), 18.2 (C-6), 17.4 (C-26), 17.1 (C-24), 15.6 (C-25); Anal. Calcd. for C_36_H_59_NO_6_: C, 71.84, H, 9.88. Found: C, 72.11, H, 9.85%. MS: *m*/*z* 602.39 [M + H]^+^ (calcd. for C_36_H_59_NO_6_, 601.43).

#### 3.1.3. General Procedure for the Guanilation of Amines **4**–**7**, **8a**, **15**, **18** and **20**

The amine (0.5 mmol) is added neat to a solution of 1,3-di-Boc-2-(trifluoromethyl-sulfonyl)guanidine (0.18 g, 0.45 mmol) or (0.60 g, 0.9 mmol) for amine **7** and Et_3_N (0.07 mL, 0.5 mmol) in CH_2_Cl_2_ (at r.t.) of compounds **4**–**7**, **8a** or in CHCl_3_ (at reflux) of compounds **15**, **18**, **20** (10 mL). The mixture was stirred for 2–12 h (TLC monitoring CH_2_Cl_2_/MeOH 20:1). After completion of the reaction, the mixture is diluted with CH_2_Cl_2_ and washed witH-NH_4_Cl, NaHCO_3_ and brine. After drying with sodium sulfate and filtering the solvent is removed under reduced pressure. The residue was chromatographed on silica gel, using hexane/EtOAc 10:1→1:1, to obtain pure compounds **9**–**13**, **15a**, **18a** and **20a**.

*N-[4-tert-Butyloxycarbonyl buthylguanidine]-3-oxo-lupane-28-amide* (**9**), White powder, 88% yield; mp 158–160 °C (EtOH); ${\left[\alpha \right]}_{D}^{23}$ +3.4° (*c* 0.59, CH_2_Cl_2_); IR (CHCl_3_) *ν*_max_ 1637, 1718 (C=O), 3337 (NH) cm^−1^; ^1^H-NMR (500 MHz, CDCl_3_) *δ*: 11.52 (s, 1H, NH in Boc), 8.30 (br s, 1H, NH–C=N), 5.70 (br s, 1H, CONH), 3.48–3.41 (m, 2H, H-2′), 3.37–3.20 (m, 2H, H-1′), 1.51, 1.48 (both s, 9H each, CH_3_ in Boc), 2.54–1.15 (m, 26H, CH, CH_2_ in pentacyclic skeleton, 4H, H-2′, H-3′), 1.08, 1.03, 0.98, 0.97, 0.95 (all s, 3H each, H-23–H-27), 0.87 (d, *J* = 6.5 Hz, 3H, H-29), 0.75 (d, *J* = 6.5 Hz, 3H, H-30); ^13^C-NMR (125 MHz, CDCl_3_) *δ*: 218.1 (C-3), 176.3 (C-28), 163.6 (C=N), 156.1, 153.3 (CONH-Boc), 83.0, 79.2 (C in Boc), 56.0 (C-17), 55.0 (C-5), 49.8 (C-9), 49.5 (C-19), 47.3 (C-4), 44.1 (C-18), 42.6 (C-14), 40.7 (C-8), 40.5 (C-4′), 39.6 (C-1), 38.7 (C-22, C-1′), 37.6 (C-13), 36.9 (C-10), 34.1 (C-2), 33.8 (C-16), 33.6 (C-7), 29.9 (C-20), 29.4 (C-15), 28.4, 28.0 (CH_3_ in Boc), 27.7 (C-23), 27.0 (C-2′, C-3′), 26.6 (C-12), 23.0 (C-21, C-29), 21.5 (C-24), 21.0 (C-11), 19.6 (C-6), 16.0 (C-25), 15.9 (C-26), 14.6 (C-30), 14.5 (C-27); Anal. Calcd. for C_45_H_76_N_4_O_6_: C, 70.27, H, 9.96. Found: C, 70.62, H, 9.91%. MS: *m*/*z* 791.56 [M + Na]^+^ (calcd. for C_45_H_76_N_4_O_6_, 768.58).

*3β-N-(2-tert-Butyloxycarbonylethylguanidine)-3-O-acetyl-lupane-28-amide* (**10**), White powder, 86% yield; mp 176–177 °C (EtOH); ${\left[\alpha \right]}_{D}^{23}$ +3.8° (*c* 0.56, CHCl_3_); IR (CHCl_3_) *ν*_max_ 1724 (C=O), 3329 (NH) cm^−1^; ^1^H-NMR (500 MHz, CDCl_3_) *δ*: 11.53 (s, 1H, NH in Boc), 8.65 (br s, 1H, NH–C=N), 6.88 (br s, 1H, CONH), 4.50–4.47 (m, 1H, H-3), 3.69–3.54 (m, 2H, H-2′), 3.47–3.35 (m, 2H, H-1′), 2.05 (s, 3H, CH_3_CO–), 1.51, 1.50 (both s, 9H each, CH_3_ in Boc), 2.46–0.96 (m, 25H, CH, CH_2_ in pentacyclic skeleton), 0.94, 0.90, 0.87, 0.86, 0.85, 0.84 (all s, 3H each, H-23–H-27 and H-29), 0.79 (d, *J* = 9.5 Hz, 1H, H-5), 0.75 (d, *J* = 7.0 Hz, 3H, H-30); ^13^C-NMR (125 MHz, CDCl_3_) *δ*: 176.9 (C-28), 171.0 (COCH_3_), 163.1 (C=N), 153.5, 153.0 (CONH-Boc), 83.5 (C in Boc), 80.9 (C-3), 77.3 (C in Boc), 56.0 (C-17), 55.4 (C-5), 50.3 (C-9), 49.7 (C-19), 44.2 (C-18), 42.5 (C-14), 41.2 (C-2′), 40.7 (C-8), 39.7 (C-1′), 38.6 (C-22), 38.4 (C-1), 37.8 (C-4), 37.5 (C-13), 37.1 (C-10), 34.3 (C-7), 33.4 (C-16), 29.8 (C-20), 29.4 (C-15), 28.3, 28.0 (CH_3_ in Boc), 27.9 (C-23), 26.9 (C-2), 23.7 (C-12), 23.1 (C-29), 23.0 (C-21), 21.3 (COCH_3_), 21.0 (C-11), 18.3 (C-6), 16.5 (C-25), 16.2 (C-24), 16.1 (C-26), 14.7 (C-30), 14.6 (C-27); Anal. Calcd. for C_45_H_76_N_4_O_7_: C, 68.84, H, 9.76. Found: C, 69.23, H, 9.69%. MS: *m*/*z* 807.54 [M + Na]^+^ (calcd. for C_45_H_76_N_4_O_7_, 784.57).

*3β-N-(4-tert-Butyloxycarbonylbutylguanidine)-3-O-acetyl-lupane-28-amide* (**11**), White powder, 82% yield; mp 148–150 °C (EtOH); ${\left[\alpha \right]}_{D}^{17}$ −4° (*c* 0.52, CHCl_3_); IR (CHCl_3_) *ν*_max_ 1640, 1720 (C=O), 3288, 3337, 3410 (NH) cm^−1^; ^1^H-NMR (500 MHz, CDCl_3_) *δ*: 11.48 (s, 1H, NH in Boc), 8.30 (t, *J* = 5.0 Hz, 1H, NH–C=N), 5.73 (t, *J* = 5.5 Hz, 1H, CONH), 4.46–4.43 (m, 1H, H-3), 3.42–3.37 (m, 2H, H-2′), 3.31–3.16 (m, 2H, H-1′), 2.01 (s, 3H, CH_3_CO–), 1.47, 1.46 (both s, 9H each, CH_3_ in Boc), 2.45–0.94 (m, 25H, CH, CH_2_ in pentacyclic skeleton, 4H, H-2′, H-3′), 0.91, 0.90, 0.83, 0.82, 0.81, 0.80 (all s, 3H each, H-23–H-27 and H-29), 0.75 (d, *J* = 9.5 Hz, 1H, H-5), 0.71 (d, *J* = 7.0 Hz, 3H, H-30); ^13^C-NMR (125 MHz, CDCl_3_) *δ*: 176.2 (C-28), 170.9 (COCH_3_), 163.6 (C=N), 156.1, 153.3 (CONH-Boc), 83.0 (C in Boc), 81.0 (C-3), 79.2 (C in Boc), 56.0 (C-17), 55.4 (C-5), 50.3 (C-9), 49.5 (C-19), 44.2 (C-18), 42.6 (C-14), 40.8 (C-8), 40.5 (C-4′), 38.7 (C-1′, C-22), 38.4 (C-1), 37.8 (C-4), 37.5 (C-13), 37.1 (C-10), 34.4 (C-7), 33.6 (C-16), 29.9 (C-3′), 29.4 (C-20), 28.3, 28.0 (CH_3_ in Boc), 27.8 (C-15), 27.3 (C-23), 26.9 (C-2′), 26.5 (C-2), 23.7 (C-12), 23.0 (C-21, C-29), 21.3 (COCH_3_), 21.0 (C-11), 18.2 (C-6), 16.5 (C-25), 16.2 (C-24, C-26), 14.6 (C-30), 14.5 (C-27); Anal. Calcd. for C_47_H_80_N_4_O_7_: C, 69.42, H, 9.92. Found: C, 69.74, H, 9.85%. MS: *m*/*z* 835.51 [M + Na]^+^ (calcd. for C_47_H_80_N_4_O_7_, 812.60).

*3β-N-[2-(N,N′-bis-tert-Butyloxycarbonylethylgyanidine)-aminoethyl]-3-O-acetyl-lupane-28-amide* (**12**), White powder, 87% yield; mp 188–190 °C (EtOH); ${\left[\alpha \right]}_{D}^{19}$ +1.02° (*c* 0.96, CHCl_3_); IR (CHCl_3_) *ν*_max_ 1641, 1722 (C=O), 3335, 3442 (NH) cm^−1^; ^1^H-NMR (500 MHz, CDCl_3_) *δ*: 11.52 (s, 2H, NH in Boc), 8.52 (br s, 2H, NH–C=N), 6.35 (t, 1H, *J* = 5.5 Hz, CONH), 4.49–4.46 (m, 1H, H-3), 3.53–3.43 (m, 4H, H-4′, H-4″), 3.41–3.22 (m, 2H, H-1′), 2.65–2.54 (m, 6H, H-2′, H-3′, H-3″), 2.04 (s, 3H, CH_3_CO–), 1.50, 1.48 (both br s, 18H each, CH_3_ in Boc), 2.32–0.98 (m, 25H, CH, CH_2_ in pentacyclic skeleton), 0.93, 0.92, 0.85, 0.84, 0.83, 0.82 (all s, 3H each, H-23–H-27 and H-29), 0.78 (d, *J* = 9.0 Hz, 1H, H-5), 0.73 (d, *J* = 6.5 Hz, 3H, H-30); ^13^C-NMR (125 MHz, CDCl_3_) *δ*: 176.7 (C-28), 171.0 (COCH_3_), 163.5 (C=N), 155.9, 153.2 (CONH-Boc), 82.9 (C in Boc), 81.0 (C-3), 79.2 (C in Boc), 55.9 (C-17), 55.5 (C-5), 54.8 (C-2′), 53.8 (C-3′, C-3″), 50.4 (C-9), 49.6 (C-19), 43.8 (C-18), 42.5 (C-14), 40.8 (C-8), 39.0 (C-4′, C-4″), 38.9 (C-22), 38.4 (C-1), 38.0 (C-13), 37.8 (C-1′), 37.2 (C-4), 37.1 (C-10), 34.4 (C-7), 33.4 (C-16), 29.8 (C-20), 29.5 (C-15), 28.3, 28.1 (CH_3_ in Boc), 28.0 (C-23), 27.0 (C-2), 23.7 (C-12), 23.1 (C-29), 23.0 (C-21), 21.3 (COCH_3_), 21.0 (C-11), 18.3 (C-6), 16.5 (C-25), 16.4 (C-24), 16.2 (C-26), 14.7 (C-30), 14.4 (C-27); Anal. Calcd. for C_60_H_104_N_8_O_11_: C, 64.72; H, 9.41. Found: C, 65.02; H, 9.34%. MS: *m*/*z* 1135.71 [M + Na]^+^ (calcd. for C_60_H_104_N_8_O_11_, 1112.78).

*3β-N-{[3-(3-tert-Butyloxycarbonylpropylgyanidine)piperazinyl]propyl}-3-O-acetyl-lupane-28-amide* (**13**), White powder, 60% yield; mp 131–134 °C (EtOH); ${\left[\alpha \right]}_{D}^{21}$ −7.9° (*c* 0.57, CHCl_3_); IR (CHCl_3_) *ν*_max_ 1640, 1722 (C=O), 3289, 3333 (NH) cm^−1^; ^1^H-NMR (500 MHz, CDCl_3_) *δ*: 11.50 (br s, 1H, NH in Boc), 8.53 (br s, 1H, NH–C=N), 6.86 (br s, 1H, CONH), 4.47–4.45 (m, 1H, H-3), 3.51–3.42 (m, 2H, H-1′′), 3.32–3.27 (m, 2H, H-1′), 2.48–2.41 (m, 14H, H-3′–H-5′, H-2″–H-5″), 2.06 (s, 3H, CH_3_CO–), 1.49 (br s, 18H, CH_3_ in Boc), 2.33–0.99 (m, 25H, CH, CH_2_ in pentacyclic skeleton, 2H, H-2′), 0.93, 0.92, 0.85, 0.84, 0.83, 0.81 (all s, 3H each, H-23–H-27 and H-29), 0.78 (d, *J* = 10.5 Hz, 1H, H-5), 0.73 (d, *J* = 6.5 Hz, 3H, H-30); ^13^C-NMR (125 MHz, CDCl_3_) *δ*: 176.4 (C-28), 171.0 (COCH_3_), 163.7 (C=N), 156.1, 153.0 (CONH-Boc), 82.8 (C in Boc), 80.9 (C-3), 79.2 (C in Boc), 56.4 (C-3′), 55.9 (C-17), 55.9 (C-3″), 55.4 (C-5), 53.1 (C-4′, C-4″, C-5′, C-5″), 50.3 (C-9), 49.6 (C-19), 44.1 (C-18), 42.6 (C-14), 40.8 (C-8), 39.8 (C-22, C-1″), 39.2 (C-1′), 38.8 (C-1), 38.4 (C-4), 37.8 (C-10), 37.4 (C-13), 34.5 (C-7), 33.7 (C-16), 29.8 (C-20), 29.4 (C-15), 28.3, 28.1 (CH_3_ in Boc), 27.9 (C-23), 26.9 (C-2), 26.9 (C-2′), 25.9 (C-2″), 23.7 (C-12), 23.1 (C-21), 23.0 (C-29), 21.3 (COCH_3_), 21.0 (C-11), 18.2 (C-6), 16.5 (C-25), 16.3 (C-24), 16.2 (C-26), 14.6 (C-30), 14.5 (C-27); Anal. Calcd. for C_53_H_92_N_6_O_7_: C, 68.79, H, 10.02. Found: C, 69.04, H, 9.96%. MS: *m*/*z* 947.58 [M + Na]^+^ (calcd. for C_53_H_92_N_6_O_7_, 924.70).

*3β-[2-tert-Butyloxycarbonylguanidine-3-hydroxy-2-(hydroxymethyl)propyl]-3-O-acetyl-lupane-28-oate* (**15a**), White powder, 63% yield; mp 106–108 °C (EtOH); ${\left[\alpha \right]}_{D}^{21}$ −5.6° (*c* 0.48, CHCl_3_); IR (CHCl_3_) *ν*_max_ 1655, 1714 (C=O), 3271, 3437 (NH) cm^−1^; ^1^H-NMR (400 MHz, CDCl_3_) *δ*: 11.47 (s, 1H, NH in Boc), 9.05 (s, 1H, NH–C=N), 4.49–4.45 (m, 1H, H-3), 4.25 (br s, 2H, H-1′), 3.82-3.77, 3.57–3.53 (both m, 2H each, H-3′, H-4′), 2.05 (s, 3H, CH_3_CO–), 1.49, 1.47 (both br s, 9H each, CH_3_ in Boc), 2.30–0.98 (m, 25H, CH, CH_2_ in pentacyclic skeleton), 0.94, 0.90, 0.88, 0.86, 0.85, 0.83 (all s, 3H each, H-23–H-27 and H-29), 0.79 (d, *J* = 9.6 Hz, 1H, H-5), 0.75 (d, *J* = 6.4 Hz, 3H, H-30); ^13^C-NMR (100 MHz, CDCl_3_) *δ*: 176.3 (C-28), 171.1 (COCH_3_), 161.8 (C=N), 155.7, 152.8 (CONH-Boc), 83.8 (C in Boc), 80.9 (C-3), 80.1 (C in Boc), 62.9 (C-1′), 62.8 (C-4′), 62.8 (C-3′), 61.9 (C-2′), 57.3 (C-17), 55.4 (C-5), 50.2 (C-9), 49.0 (C-19), 44.0 (C-18), 42.5 (C-14), 40.7 (C-8), 38.4 (C-22), 38.0 (C-1), 37.8 (C-4), 37.1 (C-13, C-10), 34.3 (C-7), 31.8 (C-16), 29.8 (C-20), 29.7 (C-15), 28.1 (CH_3_ in Boc), 28.0 (CH_3_ in Boc, C-23), 26.9 (C-2), 23.7 (C-12), 23.0 (C-29), 22.7 (C-21), 21.3 (COCH_3_), 20.9 (C-11), 18.2 (C-6), 16.5 (C-25), 16.2 (C-24), 15.9 (C-26), 14.7 (C-30), 14.6 (C-27); Anal. Calcd. for C_47_H_79_N_3_O_10_: C, 66.72, H, 9.41. Found: C, 67.13, H, 9.36%. MS: *m*/*z* 868.45 [M + Na]^+^ (calcd. for C_47_H_79_N_3_O_10_, 845.58).

*3β-[2-tert-Butyloxycarbonylguanidine-3-hydroxy-2-(hydroxymethyl)propyl]-3-O-acetyl-urs-12-en-28-oate* (**18a**), White powder, 75% yield; mp 118–120 °C (EtOH); ${\left[\alpha \right]}_{D}^{21}$ +31.3° (*c* 0.53, CHCl_3_); IR (CHCl_3_) *ν*_max_ 1653, 1729 (C=O), 3271, 3443 (NH) cm^−1^; ^1^H-NMR (500 MHz, CDCl_3_) *δ*: 11.50 (s, 1H, NH in Boc), 9.06 (s, 1H, NH–C=N), 5.26 (br s, 1H, H-12), 4.51–4.48 (m, 1H, H-3), 4.13 (br s, 2H each, H-1′), 3.82–3.76 (m, 2H, H-3′), 3.54–3.52 (m, 2H, H-4′), 2.26 (d, *J* = 11.0 Hz, 1H, H-18), 2.04 (s, 3H, CH_3_CO–), 1.99–0.81 (m, 22H, CH, CH_2_ in pentacyclic skeleton), 1.50, 1.47 (both br s, 9H each, CH_3_ in Boc), 1.07, 0.94, 0.93, 0.86, 0.85, 0.83, 0.75 (all s, 3H each, H-23–H-27, H-29 and H-30); ^13^C-NMR (125 MHz, CDCl_3_) *δ*: 177.4 (C-28), 171.0 (COCH_3_), 161.8 (C=N), 155.6, 152.8 (CONH-Boc), 138.1 (C-13), 125.8 (C-12), 83.7 (C in Boc), 80.9 (C-3), 79.9 (C in Boc), 63.4 (C-1′), 62.9 (C-3′), 62.6 (C-4′), 61.6 (C-2′), 55.3 (C-5), 52.8 (C-18), 48.5 (C-17), 47.5 (C-9), 42.0 (C-14), 39.5 (C-8), 39.0 (C-4), 38.7 (C-19), 38.3 (C-20), 37.7 (C-1), 36.8 (C-22), 36.5 (C-10), 32.9 (C-7), 30.6 (C-21), 28.1 (CH_3_ in Boc), 28.0 (C-15), 28 (C-23), 24.1 (C-16), 23.6 (C-2), 23.5 (C-27), 23.3 (C-11), 21.3 (C-30), 21.0 (COCH_3_), 18.2 (C-6), 17.0 (C-29), 16.9 (C-26), 16.7 (C-24), 15.5 (C-25); Anal. Calcd. for C_47_H_77_N_3_O_10_: C, 66.87, H, 9.19. Found: C, 67.27, H, 9.14%. MS: *m*/*z* 866.43 [M + Na]^+^ (calcd. for C_47_H_77_N_3_O_10_, 843.56).

*3β-[2-tert-Butyloxycarbonylguanidine-3-hydroxy-2-(hydroxymethyl)propyl]-3-O-acetyl-olean-12-en-28-oate* (**20a**), White powder, 79% yield; mp 140–142 °C (EtOH); ${\left[\alpha \right]}_{D}^{19}$ +30.4° (*c* 0.56, CHCl_3_); IR (CHCl_3_) *ν*_max_ 1618, 1727 (C=O), 3434 (NH) cm^−1^; ^1^H-NMR (400 MHz, CDCl_3_) *δ*: 11.49 (s, 1H, NH in Boc), 9.03 (s, 1H, NH–C=N), 5.30 (br s, 1H, H-12), 5.10 (br s, 1H, OH), 4.49–4.45 (m, 1H, H-3), 4.26, 4.15 (both d, *J* = 11.6 Hz, 1H each, H-1′), 3.77, 3.54 (both d, *J* = 12.0 Hz, *J* = 11.6 Hz, 2H each, H-3′, H-4′), 2.85 (d, *J* = 10.0 Hz, 1H, H-18), 2.03 (s, 3H, CH_3_CO–), 1.99–0.80 (m, 22H, CH, CH_2_ in pentacyclic skeleton), 1.51, 1.48 (both br s, 9H each, CH_3_ in Boc), 1.12, 0.91, 0.90, 0.89, 0.85, 0.84, 0.71 (3H each, all s, H-23–H-27, H-29 and H-30); ^13^C-NMR (100 MHz, CDCl_3_) *δ*: 177.4 (C-28), 171.0 (COCH_3_), 161.8 (C=N), 155.6, 152.8 (CONH-Boc), 138.1 (C-13), 125.8 (C-12), 83.7 (C in Boc), 80.9 (C-3), 79.9 (C in Boc), 63.4 (C-1′), 62.9 (C-3′), 62.6 (C-4′), 61.6 (C-2′), 55.3 (C-5), 52.8 (C-18), 48.5 (C-17), 47.5 (C-9), 42.0 (C-14), 39.5 (C-8), 39.0 (C-4), 38.7 (C-19), 38.3 (C-20), 37.7 (C-1), 36.8 (C-22), 36.5 (C-10), 32.9 (C-7), 30.6 (C-21), 28.1 (CH_3_ in Boc), 28.0 (C-15, C-23), 24.1 (C-16), 23.6 (C-2), 23.5 (C-27), 23.3 (C-11), 21.3 (C-30), 21.0 (COCH_3_), 18.2 (C-6), 17.0 (C-29), 16.9 (C-26), 16.7 (C-24), 15.5 (C-25); Anal. Calcd. for C_47_H_77_N_3_O_10_: C, 66.87, H, 9.19. Found: C, 67.24, H, 9.14%. MS: *m*/*z* 866.47 [M + Na]^+^ (calcd. for C_47_H_77_N_3_O_10_, 843.56).

#### 3.1.4. General Procedure for the Synthesis of Compounds **9a**–**13a**, **15b**, **18b** and **20b**

Compounds **9**–**13** and **15a**, **18a**, **20a** (0.2 mmol) in 1 mL of dry CH_2_Cl_2_ were treated with TFA (1 mL) and the mixture was stirred for 4–6 h at room temperature (TLC control, hexane:EtOAc, 1:1, *v*/*v*). The solution was evaporated to dryness to obtain pure compounds **9a**–**13a**, **15b**, **18b** and **20b**.

*N-(4-Butylgyanidine)-3-oxolupane-28-amide trifluoroacetate* (**9a**), White powder, 96% yield; mp 142–144 °C (EtOH); ${\left[\alpha \right]}_{D}^{19}$ −0.6° (*c* 0.34, CHCl_3_); IR (CHCl_3_) *ν*_max_ 1669 (C=O), 3206, 3437 (NH) cm^−1^; ^19^F-NMR (376.50 MHz, CDCl_3_) *δ*: −75.91; ^1^H-NMR (400 MHz, CDCl_3_) *δ*: 11.13 (br s, 1H, NH in Boc), 7.50 (br s, 1H, NH–C=N), 6.82 (br s, 2H, NH_2_), 6.39 (br s, 1H, CONH), 3.26 (m, 4H, H-1′, H-4′), 2.45–1.19 (m, 26H, CH, CH_2_ in pentacyclic skeleton, 4H, H-2′, H-3′), 1.07, 1.01, 0.96, 0.92, 0.90 (all s, 3H each, H-23–H-27), 0.85 (d, *J* = 6.0 Hz, 3H, H-29), 0.75 (d, *J* = 6.0 Hz, 3H, H-30); ^13^C-NMR (100 MHz, CDCl_3_) *δ*: 220.4 (C-3), 178.8 (C-28), 161.4 (q, *J*_C,F_ = 37 Hz), 157.2 (C=N), 117.5 (q, *J*_C,F_ = 288 Hz), 56.4 (C-17), 54.8 (C-5), 49.6 (C-9), 49.2 (C-19), 47.4 (C-4), 44.4 (C-18), 42.7 (C-14), 40.9 (C-1, C-4′), 40.7 (C-8), 38.8 (C-22), 38.1 (C-1′), 38.0 (C-13), 36.8 (C-10), 34.2 (C-2), 33.6 (C-16), 33.3 (C-7), 29.9 (C-20), 29.7 (C-3′), 29.4 (C-15), 27.0 (C-2′), 26.7 (C-23), 25.5 (C-12), 23.0 (C-21), 22.9 (C-29), 20.9 (C-11, C-24), 19.6 (C-6), 15.8 (C-25), 15.6 (C-26), 14.5 (C-30), 14.4 (C-27); Anal. Calcd. for C_37_H_61_F_3_N_4_O_4_: C, 65.08, H, 9.00. Found: C, 65.52, H, 8.94%. MS: *m*/*z* 569.43 [M + H]^+^ (calcd. for C_35_H_60_N_4_O_2_, 568.47).

*3β-N-(2-Ethylgyanidine)-3-O-acetyl-lupane-28-amide trifluoroacetate* (**10a**), White powder, 93% yield; mp 132–134 °C (EtOH); ${\left[\alpha \right]}_{D}^{19}$ −5.6° (*c* 0.31, CHCl_3_); IR (CHCl_3_) *ν*_max_ 1671 (C=O), 3351 (NH) cm^−1^; ^19^F-NMR (470.59 MHz, CDCl_3_) *δ*: −75.80; ^1^H-NMR (500 MHz, CDCl_3_) *δ*: 10.63 (br s, 1H, NH in Boc), 8.05 (br s, 1H, NH–C=N), 7.00 (br s, 2H, NH_2_), 6.79 (br s, 1H, CONH), 4.48–4.45 (m, 1H, H-3), 3.39–3.28 (m, 4H, H-1′, H-2′), 2.09 (s, 3H, CH_3_CO–), 2.31–1.01 (m, 25H, CH, CH_2_ in pentacyclic skeleton), 0.95, 0.92, 0.89, 0.86, 0.85, 0.83 (all s, 3H each, H-23–H-27 and H-29), 0.79 (d, *J* = 11.0 Hz, 1H, H-5), 0.75 (d, *J* = 6.0 Hz, 3H, H-30); ^13^C-NMR (125 MHz, CDCl_3_) *δ*: 179.6 (C-28), 171.6 (COCH_3_), 161.2 (q, *J*_C,F_ = 37 Hz), 157.9 (C=N), 117.2 (q, *J*_C,F_ = 287 Hz), 81.2 (C-3), 56.4 (C-17), 55.4 (C-5), 50.2 (C-9), 49.3 (C-19), 44.3 (C-18), 42.6 (C-14), 40.7 (C-1, C-2′), 40.6 (C-8), 38.6 (C-1′), 38.4 (C-22), 37.8 (C-4, C-13), 37.0 (C-10), 34.2 (C-7), 33.1 (C-16), 29.9 (C-20), 29.3 (C-15), 27.9 (C-23), 26.9 (C-2), 23.6 (C-12), 23.0 (C-21, C-29), 21.3 (COCH_3_), 21.0 (C-11), 18.1 (C-6), 16.4 (C-25), 16.0 (C-24), 15.9 (C-26), 14.5 (C-27, C-30); Anal. Calcd. for C_37_H_61_F_3_N_4_O_5_: C, 63.59, H, 8.80. Found: C, 63.88, H, 8.73%. MS: *m*/*z* 585.62 [M + H]^+^ (calcd. for C_35_H_60_N_4_O_3_, 584.47).

*3β-N-(4-Butylgyanidine)-3-O-acetyl-lupane-28-amide trifluoroacetate* (**11a**), White powder, 95% yield; mp 148–150 °C (EtOH); ${\left[\alpha \right]}_{D}^{17}$ −12° (*c* 0.49, CHCl_3_); IR (CHCl_3_) *ν*_max_ 1672 (C=O), 3207, 3354 (NH) cm^−1^; ^19^F-NMR (470.59 MHz, CDCl_3_) *δ*: −77.19; ^1^H-NMR (500 MHz, MeOD) *δ*: 4.48–4.45 (m, 1H, H-3), 3.28–3.15 (m, 4H, H-1′, H-4′), 2.04 (s, 3H, CH_3_CO–), 2.61–1.04 (m, 25H, CH, CH_2_ in pentacyclic skeleton, 4H, H-2′, H-3′), 1.02, 0.98, 0.92, 0.90, 0.89, 0.88 (3H each, all s, H-23–H-27 and H-29), 0.84 (d, *J* = 11.0 Hz, 1H, H-5), 0.79 (d, *J* = 7.0 Hz, 3H, H-30); ^13^C-NMR (125 MHz, MeOD) *δ*: 179.6 (C-28), 172.9 (COCH_3_), 161.2 (q, *J*_C,F_ = 37 Hz), 158.8 (C=N), 117.2 (q, *J*_C,F_ = 287 Hz), 82.6 (C-3), 57.5 (C-17), 57.0 (C-5), 51.9 (C-9), 51.1 (C-19), 45.5 (C-18), 43.8 (C-14), 42.2 (C-8, C-4′), 40.0 (C-22), 39.8 (C-1), 39.4 (C-1′), 39.0 (C-4), 38.9 (C-13), 38.4 (C-10), 35.8 (C-7), 34.1 (C-16), 31.3 (C-20), 30.7 (C-3′), 28.7 (C-15), 28.5 (C-23), 28.1 (C-2′), 27.4 (C-2), 24.8 (C-12), 24.2 (C-29), 23.7 (C-21), 22.5 (C-11), 21.3 (COCH_3_), 19.4 (C-6), 17.2 (C-25), 17.0 (C-24, C-26), 15.3 (C-30), 15.2 (C-27); Anal. Calcd. for C_39_H_65_F_3_N_4_O_5_: C, 64.44, H, 9.01. Found: C, 64.87, H, 8.94%. MS: *m*/*z* 613.48 [M + H]^+^ (calcd. for C_37_H_64_N_4_O_3_, 612.50).

*3β-N-[2-(N,N*′*-bis-Ethylgyanidine)-aminoethyl]-3-O-acetyl-lupane-28-amide trifluoroacetate* (**12a**), White powder, 96% yield; mp 116–118 °C (EtOH); ${\left[\alpha \right]}_{D}^{17}$ −10.5° (*c* 0.2, C_2_H_5_OH); IR (CHCl_3_) *ν*_max_ 1681 (C=O), 3199, 3362 (NH) cm^−1^; ^19^F-NMR (470.59 MHz, MeOD) *δ*: −76.98; ^1^H-NMR (500 MHz, MeOD) *δ*: 4.48–4.44 (m, 1H, H-3), 3.73–3.70 (m, 4H, H-4′, H-4″), 3.56–3.53 (m, 2H, H-1′), 3.46 (t, *J* = 6.5 Hz, 4H, H-3′, H-3″), 3.29–3.26 (m, 2H, H-2′), 2.04 (s, 3H, CH_3_CO–), 2.52–1.06 (m, 25H, CH, CH_2_ in pentacyclic skeleton), 1.02, 0.97, 0.92, 0.90, 0.88, 0.87 (all s, 3H each, H-23–H-27 and H-29), 0.85 (d, *J* = 11.5 Hz, 1H, H-5), 0.79 (d, *J* = 6.5 Hz, 3H, H-30); ^13^C-NMR (125 MHz, MeOD) *δ*: 181.2 (C-28), 173.0 (COCH_3_), 161.7 (q, *J*_C,F_ = 37 Hz), 159.1 (C=N), 117.5 (q, *J*_C,F_ = 288 Hz), 35.6 (C-4″), 53.4 (C-3″), 82.6 (C-3), 57.6 (C-17), 56.9 (C-5), 53.9 (C-2′), 53.4 (C-3′), 51.8 (C-9), 51.0 (C-19), 45.5 (C-18), 43.8 (C-14), 42.2 (C-8), 39.7 (C-1), 39.7 (C-22), 39.0 (C-13, C-4), 38.4 (C-1′), 37.8 (C-10), 35.6 (C-4′, C-7), 33.8 (C-16), 31.3 (C-20), 30.7 (C-15), 28.6 (C-23), 28.4 (C-2), 24.8 (C-12), 24.1 (C-21), 23.5 (C-29), 22.3 (C-11), 21.3 (COCH_3_), 19.4 (C-6), 17.1 (C-25), 17.0 (C-24), 16.9 (C-26), 15.2 (C-30), 15.1 (C-27); Anal. Calcd. for C_44_H_74_F_6_N_8_O_7_: C, 56.16, H, 7.93. Found: C, 56.63, H, 7.86%. MS: *m*/*z* 713.59 [M + H]^+^ (calcd. for C_40_H_72_N_8_O_3_, 712.57).

*3β-N-{[3-(3-Propylguanidine)piperazinyl]propyl}-3-O-acetyl-lupane-28-amide trifluoroacetate* (**13a**), White powder, 96% yield; mp 92–94 °C (EtOH); ${\left[\alpha \right]}_{D}^{19}$ +8° (*c* 0.54, CHCl_3_); IR (CHCl_3_) *ν*_max_ 1673, 1773 (C=O), 3190, 3367 (NH) cm^−1^; ^19^F-NMR (470.59 MHz, MeOD) *δ*: −77.25; ^1^H-NMR (500 MHz, MeOD) *δ*: 4.48–4.45 (m, 1H, H-3), 3.49–3.40 (m, 4H, H-2″, H-3″), 3.31–3.22 (m, 8H, H-4′, H-4″, H-5′, H-5″), 3.13–3.10 (m, 2H, H-1″), 3.00–2.98 (m, 2H, H-1′), 2.58–0.84 (m, 26H, CH, CH_2_ in pentacyclic skeleton, 4H, H-2′, H-3′), 2.08 (s, 3H, CH_3_CO–), 1.02, 0.99, 0.92, 0.89, 0.86, 0.84 (all s, 3H each, H-23–H-27 and H-29), 0.80 (d, *J* = 7.0 Hz, 3H, H-30); ^13^C-NMR (125 MHz, MeOD) *δ*: 180.2 (C-28), 173.0 (COCH_3_), 161.7 (q, *J*_C,F_ = 37 Hz), 158.9 (C=N), 117.5 (q, *J*_C,F_ = 288 Hz), 82.6 (C-3), 57.6 (C-17), 57.0 (C-5), 56.0 (C-3″), 55.2 (C-3′), 51.9 (C-9), 51.0 (C-19), 50.1 (C-4′, C-4″, C-5′, C-5″), 45.5 (C-18), 43.8 (C-14), 42.2 (C-8), 39.9 (C-1′), 39.8 (C-1″), 39.6 (C-1, C-22), 39.0 (C-4), 38.9 (C-13), 38.4 (C-10), 35.7 (C-7), 34.0 (C-16), 31.3 (C-20), 30.7 (C-15), 28.6 (C-23), 28.5 (C-2″), 25.6 (C-2′), 24.9 (C-2), 24.2 (C-12), 23.6 (C-21, C-29), 22.4 (C-11), 21.3 (COCH_3_), 19.4 (C-6), 17.1 (C-25), 17.0 (C-24), 16.9 (C-26), 15.2 (C-30), 15.1 (C-27); Anal. Calcd. for C_45_H_77_F_3_N_6_O_5_: C, 64.41, H, 9.25. Found: C, 64.88, H, 9.19. MS: *m*/*z* 747.58 [M + Na]^+^ (calcd. for C_43_H_76_N_6_O_3_, 724.60).

*3β-[2-Guanidine-3-hydroxy-2-(hydroxymethyl)propyl]-3-O-acetyl-lupane-28-oate trifluoroacetate* (**15b**), White powder, 95% yield; mp 126–129 °C (EtOH); ${\left[\alpha \right]}_{D}^{19}$ −9.5° (*c* 0.34, CHCl_3_); IR (CHCl_3_) *ν*_max_ 1620, 1698 (C=O), 3437 (NH) cm^−1^; ^19^F-NMR (470.59 MHz, CDCl_3_) *δ*: −75.97; ^1^H-NMR (500 MHz, MeOD) *δ*: 4.50–4.47 (m, 1H, H-3), 4.31 (2H, m, H-1′), 3.75 (m, 4H, H-3′, H-4′), 2.18–0.99 (m, 26H, CH, CH_2_ in pentacyclic skeleton), 2.08 (s, 3H, CH_3_CO–), 0.98, 0.97, 0.96, 0.87, 0.86 (all s, 3H each, H-23–H-27), 0.89 (d, *J* = 6.0 Hz, 3H, H-29), 0.76 (d, *J* = 6.0 Hz, 3H, H-30); ^13^C-NMR (125 MHz, MeOD) *δ*: 177.4 (C-28), 172.3 (COCH_3_), 161.4 (q, *J*_C,F_ = 37 Hz), 157.5 (C=N), 117.5 (q, *J*_C,F_ = 288 Hz), 81.7 (C-3), 63.1 (C-3′, C-4′), 62.0 (C-2′), 60.2 (C-1′), 57.5 (C-17), 55.4 (C-5), 50.2 (C-9), 48.8 (C-19), 44.1 (C-18), 42.6 (C-14), 40.7 (C-8), 38.4 (C-22), 38.2 (C-1), 37.8 (C-4, C-13), 37.0 (C-10), 34.2 (C-7), 31.9 (C-16), 29.7 (C-20), 29.0 (C-15), 27.9 (C-23), 26.8 (C-2), 23.6 (C-12), 22.9 (C-29, C-21), 21.3 (COCH_3_), 20.9 (C-11), 18.1 (C-6), 16.4 (C-25), 16.1 (C-24), 15.8 (C-26), 14.6 (C-27, C-30); Anal. Calcd. for C_39_H_64_F_3_N_3_O_8_: C, 61.64; H, 8.49. Found: C, 62.34; H, 8.43%. MS: *m*/*z* 646.39 [M + H]^+^ (calcd. for C_37_H_63_N_3_O_6_, 645.47).

*3β-[2-Guanidine-3-hydroxy-2-(hydroxymethyl)propyl]-3-O-acetyl-urs-12-en-28-oate trifluoroacetate* (**18b**), White powder, 98% yield; mp 124–126 °C (EtOH); ${\left[\alpha \right]}_{D}^{19}$ +30.0° (*c* 0.72, CH_2_Cl_2_); IR (CHCl_3_) *ν*_max_ 1619, 1682 (C=O), 3438 (NH) cm^−1^; ^19^F-NMR (470.59 MHz, MeOD) *δ*: −77.17; ^1^H-NMR (500 MHz, MeOD) *δ*: 5.29 (br s, 1H, H-12), 4.49–4.46 (m, 1H, H-3), 4.31, 4.17 (both d, *J* = 11.5 Hz, 1H each, H-1′), 3.75 (m, 4H, H-3′, H-4′), 2.24 (d, *J* = 11.0 Hz, 1H, H-18), 2.04 (s, 3H, CH_3_CO–), 2.13–0.87 (m, 22H, CH, CH_2_ in pentacyclic skeleton), 1.15, 1.01, 0.97, 0.98, 0.92, 0.90, 0.81 (3H each, all s, H-23–H-27, H-29 and H-30); ^13^C-NMR (125 MHz, MeOD) *δ*: 179.2 (C-28), 173.0 (COCH_3_), 161.4 (q, *J*_C,F_ = 37 Hz), 159.2 (C=N), 140.1 (C-13), 127.2 (C-12), 117.5 (q, *J*_C,F_ = 288 Hz), 82.5 (C-3), 64.3 (C-3′), 64.2 (C-4′), 62.7 (C-2′), 62.2 (C-1′), 56.8 (C-5), 54.4 (C-18), 49.5 (C-17), 48.6 (C-9), 43.3 (C-14), 40.9 (C-8), 40.4 (C-4, C-19), 39.6 (C-20), 38.8 (C-1), 38.1 (C-22), 38.0 (C-10), 34.2 (C-7), 31.7 (C-21), 29.2 (C-15), 28.7 (C-23), 25.5 (C-16), 24.6 (C-2), 24.5 (C-27), 24.4 (C-11), 21.6 (C-30), 21.3 (COCH_3_), 19.4 (C-6), 17.9 (C-29), 17.8 (C-26), 17.3 (C-24), 16.2 (C-25); Anal. Calcd. for C_39_H_62_F_3_N_3_O_8_: C, 61.80, H, 8.25. Found: C, 62.33, H, 8.19%. MS: *m*/*z* 644.47 [M + H]^+^ (calcd. for C_37_H_61_N_3_O_6_, 643.46).

*3β-[2-Guanidine-3-hydroxy-2-(hydroxymethyl)propyl]-3-O-acetyl-olean-12-en-28-oate trifluoroacetate* (**20b**), White powder, 97% yield; mp 130–132 °C (EtOH); ${\left[\alpha \right]}_{D}^{17}$ +26.6° (*c* 0.53, C_2_H_5_OH); IR (CHCl_3_) *ν*_max_ 1619, 1682 (C=O), 3438 (NH) cm^−1^; ^19^F-NMR (470.59 MHz, CDCl_3_) *δ*: −77.24; ^1^H-NMR (500 MHz, MeOD) *δ*: 5.31 (br s, 1H, H-12), 4.49–4.46 (m, 1H, H-3), 4.33, 4.20 (d, *J* = 11.5 Hz, 1H each, H-1′), 3.75 (m, 4H, H-3′, H-4′), 2.89 (d, 1H, *J* = 9.5 Hz, H-18), 2.09–0.88 (m, 22H, CH, CH_2_ in pentacyclic skeleton), 2.04 (s, 3H, CH_3_CO–), 1.19, 1.00, 0.97, 0.95, 0.90, 0.89, 0.79 (all s, 3H each, H-23–H-27, H-29 and H-30); ^13^C-NMR (125 MHz, MeOD) *δ*: 178.9 (C-28), 173.0 (COCH_3_), 161.4 (q, *J*_C,F_ = 37 Hz), 159.2 (C=N), 145.0 (C-13), 124.1 (C-12), 117.5 (q, *J*_C,F_ = 288 Hz), 82.6 (C-3), 64.3 (C-4′), 64.2 (C-3′), 62.7 (C-2′), 62.3 (C-1′), 56.9 (C-5), 48.5 (C-9, C-17), 47.1 (C-19), 43.0 (C-14), 42.9 (C-18), 40.8 (C-8), 39.5 (C-1), 38.9 (C-4), 38.3 (C-10), 34.9 (C-22), 33.9 (C-30), 33.7 (C-7), 33.6 (C-21), 31.7 (C-20), 28.9 (C-15), 28.7 (C-23), 26.6 (C-27), 24.7 (C-11, C-29), 24.3 (C-2), 24.2 (C-16), 21.3 (COCH_3_), 19.5 (C-6), 17.9 (C-26), 17.3 (C-24), 16.1 (C-25); Anal. Calcd. for C_39_H_62_F_3_N_3_O_8_: C, 61.80, F, 7.52, H, 8.25. Found: C, 62.12, H, 8.21%. MS: *m*/*z* 644.57 [M + H]^+^ (calcd. for C_37_H_61_N_3_O_6_, 643.46).

#### 3.1.5. General Procedure for the Synthesis of Compounds **9b**–**12b**, **15c**, **18c** and **20c**

The compounds **9a**–**12a**, **15b**, **18b**, **20b** (0.2 g) were dissolved in 2 mL MeOH and 5M HCl was added dropwise until the precipiate formed. The solution was evaporated to dryness and this procedure was repeated three times. The precipitate which formed was filtered off and washed with water to pH = 7. The salts **9b**–**12b** and **15c**, **18c**, **20c** were obtained as white solids with a quantitative yield.

*N-(4-Butylgyanidine)-3-oxolupane-28-amide dihydrochloride* (**9b**), White powder, 87% yield; mp 176–178 °C (EtOH); ${\left[\alpha \right]}_{D}^{21}$ −11° (*c* 0.29, C_2_H_5_OH); IR (CHCl_3_) *ν*_max_ 1721 (C=O), 3342 (NH) cm^−1^; ^1^H-NMR (500 MHz, *d*_6_-DMSO) *δ*: 7.71 (br s, 1H, NH), 7.59 (br s, 1H, CONH), 3.10–3.01 (m, 4H, H-1′, H-4′), 2.61–1.04 (m, 26H, CH, CH_2_ in pentacyclic skeleton, 4H, H-2′, H-3′), 0.99, 0.93, 0.91, 0.87, 0.86 (3H each, all s, H-23–H-27), 0.81 (d, *J* = 6.5 Hz, 3H, H-29), 0.71 (d, *J* = 7.0 Hz, 3H, H-30); ^13^C-NMR (125 MHz, *d*_6_-DMSO) *δ*: 217.1 (C-3), 176.1 (C-28), 157.4 (C=N), 55.7 (C-17), 54.3 (C-5), 49.6 (C-9), 49.5 (C-19), 47.0 (C-4), 43.8 (C-18), 42.6 (C-14), 40.9 (C-4′), 40.3 (C-8), 39.7 (C-1), 38.1 (C-22, C-1′), 37.0 (C-13), 36.8 (C-10), 34.1 (C-2), 33.8 (C-16), 32.7 (C-7), 30.0 (C-20), 29.4 (C-15), 27.2 (C-3′), 27.0 (C-2′), 26.9 (C-23), 26.4 (C-12), 23.6 (C-29), 23.1 (C-21), 21.7 (C-11), 21.2 (C-24), 19.7 (C-6), 16.2 (C-25), 16.1 (C-26), 15.0 (C-30), 14.6 (C-27); Anal. Calcd. for C_35_H_62_Cl_2_N_4_O_2_: C, 65.50, Cl, 11.05, H, 9.74. Found: C, 65.97, Cl, 11.78, H, 9.68%. MS: *m*/*z* 569.49 [M + H]^+^ (calcd. for C_35_H_60_N_4_O_2_, 568.47).

*3β-N-(2-Ethylgyanidine)-3-O-acetyl-lupane-28-amide hydrochloride* (**10b**), White powder, 82% yield; mp 192–194 °C (EtOH); ${\left[\alpha \right]}_{D}^{17}$ −16° (*c* 0.23, C_2_H_5_OH); IR (CHCl_3_) *ν*_max_ 1652, 1716 (C=O), 3155, 3327 (NH) cm^−1^; ^1^H-NMR (500 MHz, MeOD) *δ*: 4.48–4.45 (m, 1H, H-3), 3.43–3.16 (m, 4H, H-1′, H-2′), 2.04 (s, 3H, CH_3_CO–), 2.60–0.81 (m, 25H, CH, CH_2_ in pentacyclic skeleton), 1.02, 0.98, 0.92, 0.90, 0.88, 0.87 (all s, 3H each, H-23–H-27 and H-29), 0.80 (d, *J* = 6.5 Hz, 3H, H-30); ^13^C-NMR (125 MHz, MeOD) *δ*: 180.8 (C-28), 173.0 (COCH_3_), 159.0 (C=N), 82.6 (C-3), 57.6 (C-17), 57.0 (C-5), 51.1 (C-9, C-19), 45.5 (C-18), 42.7 (C-14, C-1′), 42.2 (C-8), 39.8 (C-1), 39.7 (C-2′), 39.4 (C-22), 39.0 (C-4), 38.9 (C-13), 38.4 (C-10), 35.7 (C-7), 34.0 (C-16), 31.4 (C-20), 30.7 (C-15), 28.6 (C-23), 28.5 (C-2), 24.8 (C-12), 24.1 (C-21), 23.6 (C-29), 22.4 (C-11), 21.3 (COCH_3_), 19.4 (C-6), 17.1 (C-25), 16.9 (C-24, C-26), 15.2 (C-30), 15.1 (C-27); Anal. Calcd. for C_35_H_61_ClN_4_O_3_: C, 67.66, Cl, 5.71, H, 9.90. Found: C, 67.99, Cl, 5.40, H, 9.84%. MS: *m*/*z* 585.54 [M + H]^+^ (calcd. for C_35_H_60_N_4_O_3_, 584.47).

*3β-N-(4-Butylgyanidine)-3-O-acetyl-lupane-28-amide hydrochloride* (**11b**), White powder, 79% yield; mp 156–158 °C (EtOH); ${\left[\alpha \right]}_{D}^{22}$ −14.5° (*c* 0.53, C_2_H_5_OH); IR (CHCl_3_) *ν*_max_ 1645, 1716 (C=O), 3168, 3338 (NH) cm^−1^; ^1^H-NMR (500 MHz, MeOD) *δ*: 7.45 (br s, 1H, NH), 4.48–4.45 (m, 1H, H-3), 3.23–3.18 (m, 4H, H-1′, H-4′), 2.04 (s, 3H, CH_3_CO–), 2.61–0.84 (m, 26H, CH, CH_2_ in pentacyclic skeleton, 4H, H-2′, H-3′), 1.01, 0.98, 0.92, 0.89, 0.88, 0.86 (all s, 3H each, H-23–H-27 and H-29), 0.79 (d, *J* = 7.0 Hz, 3H, H-30); ^13^C-NMR (125 MHz, MeOD) *δ*: 179.6 (C-28), 172.9 (COCH_3_), 158.8 (C=N), 82.6 (C-3), 57.5 (C-17), 57.0 (C-5), 51.9 (C-9), 51.1 (C-19), 45.5 (C-18), 43.8 (C-14), 42.4 (C-8), 42.2 (C-4′), 40.0 (C-22, C-1′), 39.8 (C-1), 39.0 (C-4), 38.9 (C-13), 38.4 (C-10), 35.8 (C-7), 34.1 (C-16), 31.4 (C-20), 30.8 (C-3′), 28.6 (C-15), 28.2 (C-23, C-2′), 27.4 (C-2), 24.8 (C-12), 24.4 (C-21), 24.2 (C-29), 22.5 (C-11), 21.3 (COCH_3_), 19.4 (C-6), 17.1 (C-25), 17 (C-24), 16.9 (C-26), 15.2 (C-30), 15.1 (C-27); Anal. Calcd. for C_37_H_65_ClN_4_O_3_: C, 68.43, Cl, 5.46, H, 10.09. Found: C, 68.88; Cl, 5.12; H, 10.02%. MS: *m*/*z* 614.51 [M + 2H]^+^ (calcd. for C_37_H_64_N_4_O_3_, 612.50).

*3β-N-[2-(N,N*′*-bis-Ethylgyanidine)-aminoethyl]-3-O-acetyl-lupane-28-amide dihydrochloride* (**12b**), White powder, 88% yield; mp 198–200 °C (EtOH); ${\left[\alpha \right]}_{D}^{17}$ −16° (*c* 0.29, C_2_H_5_OH); IR (CHCl_3_) *ν*_max_ 1637, 1672, 1734 (C=O), 3162, 3271, 3324 (NH) cm^−1^; ^1^H-NMR (500 MHz, MeOD) *δ*: 4.48–4.45 (m, 1H, H-3), 3.90–3.83 (m, 4H, H-4′, H-4″), 3.66–3.59 (m, 6H, H-2′, H-3′, H-3″), 3.38–3.33 (m, 2H, H-1′), 2.04 (s, 3H, CH_3_CO–), 2.51–0.85 (m, 26H, CH, CH_2_ in pentacyclic skeleton), 1.03, 0.99, 0.93, 0.90, 0.88, 0.87 (all s, 3H each, H-23–H-27 and H-29), 0.80 (d, *J* = 6.5 Hz, 3H, H-30); ^13^C-NMR (125 MHz, MeOD) *δ*: 181.0 (C-28), 172.9 (COCH_3_), 158.9 (C=N), 82.5 (C-3), 57.7 (C-17), 56.9 (C-5), 53.7 (C-3′, C-3″), 53.6 (C-2′), 51.8 (C-9), 51.0 (C-19), 45.5 (C-18), 43.8 (C-14), 42.2 (C-8), 39.7 (C-22), 39.0 (C-1, C-13), 38.4 (C-4), 37.7 (C-10, C-1′), 35.7 (C-7), 35.0 (C-4′, C-4″), 33.9 (C-16), 31.3 (C-20), 30.8 (C-15), 28.6 (C-23), 28.4 (C-2), 24.8 (C-12), 24.2 (C-21), 23.6 (C-29), 22.4 (C-11), 21.3 (COCH_3_), 19.4 (C-6), 17.1 (C-25, C-24), 16.9 (C-26), 15.2 (C-30), 15.1 (C-27); Anal. Calcd. for C_40_H_74_Cl_2_N_8_O_3_: C, 61.13, Cl, 9.02, H, 9.49. Found: C, 61.55, Cl, 11.40, H, 9.42%. MS: *m*/*z* 713.51 [M + H]^+^ (calcd. for C_40_H_72_N_8_O_3_, 712.57).

*3β-[2-Guanidine-3-hydroxy-2-(hydroxymethyl)propyl]-3-O-acetyl-lupane-28-oate hydrochloride* (**15c**), White powder, 68% yield; mp 136–138 °C (EtOH); ${\left[\alpha \right]}_{D}^{22}$ −14.5° (*c* 0.53, C_2_H_5_OH); IR (CHCl_3_) *ν*_max_ 1673, 1733 (C=O), 3325 (NH) cm^−1^; ^1^H-NMR (500 MHz, MeOD) *δ*: 4.48–4.45 (m, 1H, H-3), 4.31 (2H, m, H-1′), 3.75 (m, 4H, H-3′, H-4′), 2.04 (s, 3H, CH_3_CO–), 2.29–0.85 (m, 26H, CH, CH_2_ in pentacyclic skeleton), 1.03, 1.00, 0.98, 0.91, 0.88, 0.87 (all s, 3H each, H-23–H-27 and H-29), 0.81 (d, *J* = 6.5 Hz, 3H, H-30); ^13^C-NMR (125 MHz, MeOD) *δ*: 177.1 (C-28, COCH_3_), 159.1 (C=N), 82.6 (C-3), 64.1 (C-3′), 64.1 (C-4′), 62.8 (C-2′), 61.8 (C-1′), 58.7 (C-17), 56.9 (C-5), 51.8 (C-9), 50.4 (C-19), 45.8 (C-18), 43.9 (C-14), 42.1 (C-8), 39.7 (C-22), 39.6 (C-1), 39.0 (C-4), 38.4 (C-10, C-13), 35.6 (C-7), 33.2 (C-16), 31.2 (C-20), 30.9 (C-15), 28.6 (C-23), 28.4 (C-2), 24.8 (C-12), 23.9 (C-21), 23.5 (C-29), 22.3 (C-11), 21.3 (COCH_3_), 19.4 (C-6), 17.1 (C-25), 16.9 (C-24), 16.8 (C-26), 15.2 (C-30), 15.1 (C-27); Anal. Calcd. for C_37_H_64_ClN_3_O_6_: C, 65.13, Cl, 5.20, H, 9.45. Found: C, 65.6 4, Cl, 4.50; H, 9.39%. MS: *m*/*z* 647.47 [M + 2H]^+^ (calcd. for C_37_H_63_N_3_O_6_, 645.47).

*3β-[2-Guanidine-3-hydroxy-2-(hydroxymethyl)propyl]-3-O-acetyl-urs-12-en-28-oate hydrochloride* (**18c**), White powder, 62% yield; mp 142–144 °C (EtOH); ${\left[\alpha \right]}_{D}^{19}$ +37° (*c* 0.51, C_2_H_5_OH); IR (CHCl_3_) *ν*_max_ 1675, 1733 (C=O), 3186, 3335 (NH) cm^−1^; ^1^H-NMR (500 MHz, MeOD) *δ*: 7.07 (s, 1H, NH), 5.30 (br s, 1H, H-12), 4.50–4.47 (m, 1H, H-3), 4.29, 4.23 (both d, *J* = 11.5 Hz, 1H each, H-1′), 3.75 (m, 4H, H-3′, H-4′), 2.25 (d, *J* = 11.5 Hz, 1H, H-18), 2.05 (s, 3H, CH_3_CO–), 2.14–0.88 (m, 22H, CH, CH_2_ in pentacyclic skeleton), 1.16, 1.02, 1.00, 0.92, 0.91, 0.90, 0.81 (all s, 3H each, H-23–H-27, H-29 and H-30); ^13^C-NMR (125 MHz, MeOD) *δ*: 178.6 (C-28), 173.0 (COCH_3_), 159.2 (C=N), 139.6 (C-13), 127.3 (C-12), 82.6 (C-3), 64.2 (C-3′, C-4′), 62.9 (C-2′), 62.3 (C-1′), 56.8 (C-5), 54.5 (C-18), 49.3 (C-17), 49.0 (C-9), 43.4 (C-14), 41.0 (C-8), 40.5 (C-4, C-19), 39.6 (C-20), 38.9 (C-1), 38.2 (C-22), 38.0 (C-10), 34.2 (C-7), 31.8 (C-21), 29.3 (C-15), 28.8 (C-23), 25.5 (C-16), 24.7 (C-2), 24.5 (C-27), 24.4 (C-11), 21.7 (C-30), 21.3 (COCH_3_), 19.5 (C-6), 17.9 (C-29), 17.8 (C-26), 17.4 (C-24), 16.2 (C-25); Anal. Calcd. for C_37_H_62_ClN_3_O_6_: C, 65.32, Cl, 5.21, H, 9.19. Found: C, 65.74, Cl, 4.51, H, 9.14%. MS: *m*/*z* 644.34 [M + H]^+^ (calcd. for C_37_H_61_N_3_O_6_, 643.46).

*3β-[2-Guanidine-3-hydroxy-2-(hydroxymethyl)propyl]-3-O-acetyl-olean-12-en-28-oate hydrochloride* (**20c**), White powder, 53% yield; mp 124–128 °C (EtOH); ${\left[\alpha \right]}_{D}^{19}$ +34° (*c* 0.34, C_2_H_5_OH); IR (CHCl_3_) *ν*_max_ 1662, 1733 (C=O), 3344 (NH) cm^−1^; ^1^H-NMR (500 MHz, MeOD) *δ*: 5.32 (br s, 1H, H-12), 4.49–4.46 (m, 1H, H-3), 4.33, 4.20 (both d, *J* = 11.5 Hz, 1H each, H-1′), 3.76 (m, 4H, H-3′, H-4′), 2.89 (d, *J* = 9.5 Hz, 1H, H-18), 2.13–0.90 (m, 22H, CH, CH_2_ in pentacyclic skeleton), 2.05 (s, 3H, CH_3_CO–), 1.20, 1.00, 0.98, 0.96, 0.94, 0.91, 0.88 (all s, 3H each, H-23–H-27, H-29 and H-30); ^13^C-NMR (125 MHz, MeOD) *δ*: 178.8 (C-28), 172.9 (COCH_3_), 159.1 (C=N), 145.0 (C-13), 124.1 (C-12), 82.6 (C-3), 64.2 (C-4′), 64.1 (C-3′), 62.8 (C-2′), 62.3 (C-1′), 56.8 (C-5), 49.0 (C-9), 48.5 (C-17), 47.1 (C-19), 43.0 (C-14), 42.9 (C-18), 40.7 (C-8), 39.5 (C-1), 38.9 (C-4), 38.2 (C-10), 34.9 (C-22), 33.9 (C-30), 33.7 (C-7, C-21), 31.7 (C-20), 28.9 (C-15), 28.7 (C-23), 26.6 (C-27), 24.7 (C-11, C-29), 24.3 (C-2), 24.2 (C-16), 21.3 (COCH_3_), 19.5 (C-6), 17.9 (C-26), 17.3 (C-24), 15.6 (C-25); Anal. Calcd. for C_37_H_62_ClN_3_O_6_: C, 65.32, Cl, 5.21, H, 9.19. Found: C, 65.74, Cl, 4.80, H, 9.12%. MS: *m*/*z* 644.44 [M + H]^+^ (calcd. for C_37_H_61_N_3_O_6_, 643.46).

*3β-N-(4-Butylgyanidine)-3-hydroxy-lupane-28-amide* (**14**), To a solution of the compound **11b** (0.33 g, 0.5 mmol) in MeOH (4 mL) and THF (4 mL) was added 4 N NaOH (4 mL). The reaction mixture was stirred at room temperature for 24 h (monitoring by TLC) and then neutralized with 20% HCl. The solution was dried under vacuum and reconstituted with CH_2_Cl_2_. The organic layer was washed with brine and dried over anhydrous MgSO_4_ and concentrated under reduced pressure to obtain pure compound 14. White powder, 79% yield; mp 260–262 °C (EtOH); ${\left[\alpha \right]}_{D}^{19}$ −10° (*c* 0.24, DMSO); IR (CHCl_3_) *ν*_max_ 1731 (C=O), 3366 (NH) cm^−1^; ^1^H-NMR (500 MHz, DMSO-d_5_) *δ*: 7.73 (br s, 1H, NH), 7.58 (br s, 1H, CONH), 4.29 (br s, 1H, OH), 3.09–2.98 (m, 5H, H-3, H-1′, H-4′), 2.19–1.02 (m, 26H, CH, CH_2_ in pentacyclic skeleton, 4H, H-2′, H-3′), 0.89, 0.87, 0.84, 0.78, 0.66 (all s, 3H each, H-23–H-27), 0.81 (d, *J* = 6.5 Hz, 3H, H-29), 0.72 (d, *J* = 6.5 Hz, 3H, H-30); ^13^C-NMR (125 MHz, DMSO-d_5_) *δ*: 176.1 (C-28), 157.4 (C=N), 77.2 (C-3), 55.7 (C-17), 55.4 (C-5), 50.3 (C-9), 49.6 (C-19), 43.8 (C-18), 42.5 (C-14), 40.9 (C-8), 40.8 (C-4′), 39.0 (C-22), 38.8 (C-1), 38.6 (C-4), 38.1 (C-1′), 37.2 (C-13), 36.9 (C-10), 34.6 (C-7), 32.8 (C-16), 30.0 (C-20), 29.4 (C-15), 28.6 (C-23), 27.4 (C-2), 27.2 (C-3′), 27.0 (C-2′), 26.4 (C-12), 23.6 (C-29), 23.2 (C-21), 21.1 (C-11), 18.5 (C-6), 16.4 (C-25), 16.3 (C-24, C-26), 15.0 (C-30), 14.7 (C-27); Anal. Calcd. for C_35_H_62_N_4_O_2_: C, 73.63, H, 10.95. Found: C, 73.74, H, 10.88%. MS: *m*/*z* 593.31 [M + Na]^+^ (calcd. for C_35_H_62_N_4_O_2_, 570.49).

### 3.2. Biology

#### 3.2.1. Cell Culturing

Cells (Jurkat, K562, U937, HeLa, HEK293 and normal Fibroblasts) were purchased from Russian Cell Culture Collection (Institute of Cytology of the Russian Academy of Sciences, Saint Petersburg, Russia) and cultured according to standard mammalian tissue culture protocols and sterile technique. Human cell lines HEK293 and HeLa were obtained from the HPA Culture Collections (Salisbury, UK). All cell lines used in the study were tested and shown to be free of mycoplasma and viral contamination.

HEK293, HeLa cell lines and fibroblasts were cultured as monolayers and maintained in Dulbecco’s modified Eagle’s medium (DMEM, Gibco BRL, Waltham, MA, USA) supplemented with 10% foetal bovine serum and 1% penicillin-streptomycin solution at 37 °C in a humidified incubator under a 5% CO_2_ atmosphere.

Cells were maintained in RPMI 1640 (Jurkat, K562, U937) (Gibco, Thermo Fisher Scientific, Waltham, MA, USA) supplemented with 4 mM glutamine, 10% FBS (Sigma, Burlington, MA, USA) and 100 units/mL penicillin-streptomycin (Sigma). All types of cells were grown in an atmosphere of 5% CO_2_ at 37 °C. The cells were subcultures at 2–3 days intervals. Adherent cells (HEK293, HeLa, fibroblasts) were suspended using trypsin/EDTA and counted after they have reached 80% confluency. Cells were then seeded in 24 well plates at 5 × 104 cells per well and incubated overnight. Jurkat, K562, U937 cells were subcultured at 2 day intervals with a seeding density of 1 × 105 cells per 24 well plates in RPMI with 10% FBS.

#### 3.2.2. Cytotoxicity Assay

Viability (Live/dead) assessment was performed by staining cells with 7-AAD (7-Aminoactinomycin D) (Biolegend, San Diego, CA, USA). Cells were treated of test compounds with six different concentrations (1, 5, 10, 15, 30 and 60 μM). After treatment, cells were harvested, washed 1–2 times with phosphate-buffered saline (PBS) and centrifuged at 400× *g* for 5 min. Cell pellets were resuspended in 200 μL of flow cytometry staining buffer (PBS without Ca^2+^ and Mg^2+^, 2.5% FBS) and stained with 5 μL of 7-AAD staining solutionfor 15 min at room temperature in the dark. Samples were acquired on NovoCyteTM 2000 FlowCytometry System (ACEA, San Diego, CA, USA) equipped with 488 nm argon laser. Detection of 7-AAD emission was collected through a 675/30 nm filter in FL4 channel.

#### 3.2.3. Viability and Apoptosis

Apoptosis was determined by flow cytometric analysis of Annexin V and 7-aminoactinomycin D staining. Briefly, 200 μL of Guava Nexin reagent (Millipore, Bedford, MA, USA) was added to 5 × 105 cells in 200 μL, and the cells were incubated with the reagent for 20 min at room temperature in the dark. The plates were treated with compounds **15**, **15c**, **18c**, **20c** and dihydrobetulinic acid at IC_50_ concentration (4, 8 and 59 μM) for 24 h and 48 h. At the end of incubation, the cells were analyzed on NovoCyteTM 2000 FlowCytometry System (ACEA). Different states of cell death were defined as follows: normal cells are localized in the lower-left quadrant (Annexin V^−^/PI^−^); early apoptotic cells are in the lower-right quadrant (Annexin V^+^/PI^−^); late apoptotic cells and necrotic cells are in the upper-right quadrant (Annexin V^+^/PI^+^); and necrotic cells are in the upper-left quadrant (Annexin V^−^/PI^+^).

#### 3.2.4. Cell Cycle Analysis

Cell cycle was analyzed using the method of propidium iodide staining. Briefly, cells were plated in 24-well round bottom plates at density 10 × 105 cells per well, centrifuged at 450× *g* for 5 min, and fixed with ice-cold 70% ethanol for 24 h at 0 °C. Cells were then washed with PBS and incubated with 250 μL of Guava Cell Cycle Reagent (Millipore, Burlington, MA, USA) for 30 min at room temperature in the dark. Samples were analyzed on NovoCyte^TM^ 2000 FlowCytometry System (ACEA, San Diego, CA, USA).

## 4. Conclusions

Novel betulinic, ursolic, and oleanolic acid derivatives, containing a guanidine moiety have been designed and synthesized in an attempt to develop potent antitumor agents. These compounds and their precursors, monoamine, diamine and triamine derivatives, were tested for cytotoxic activity on various human tumor cell lines. Guanidine-functionalized triterpenoids demonstrated higher cytotoxicity in Jurkat cells, compared with original triterpenoic acids. Most of the tested guanidine derivatives showed higher IC_50_ values than amines, but were less toxic to human fibroblasts. The lead molecules—dihydrobetulinic acid amine **15**, its guanidine derivative **15c**, and guanidinium salts of ursolic and oleanolic acids **18c** and **20c** were selected for extended biological testing by using flow cytometry analysis. Our results showed that the antitumor activity of compounds **15**, **15c**, and **18c** is caused by apoptotic processes and induction of cell cycle arrest in the S-phase. Nevertheless, addition information concerning the molecular mechanisms and targets of these triterpene acid derivatives is needed.

## Figures and Tables

**Table 1:** Cytotoxicity of dihydrobetulinic-, ursolic- and oleanolic acids and compounds **4**–**6**, **8a**, **9b**–**12b**, **12a**, **13a**, **14**, **15**, **15c**, **18b**,**c**, and **20b**,**c** against Jurkat, K562, Hek293, HeLa, U937, and normal fibroblast cells.

Compound	IC_50_ (µmol/L) ^a^					
	Jurkat	K562	U937	HEK293	HeLa	Fibroblasts
**Dihydro BA**	59 ± 0.31	44 ± 0.24	39 ± 0.38	128 ± 0.32	132 ± 0.47	517 ± 0.26
**UA**	23 ± 0.34	68 ± 0.11	17 ± 0.12	96 ± 0.22	88 ± 0.35	324 ± 0.16
**OA**	271 ± 0.19	235 ± 0.24	186 ± 0.18	247 ± 0.27	258 ± 0.31	694 ± 0.17
**4**	1.4 ± 0.41	1.3 ± 0.49	1.7 ± 0.36	2.4 ± 0.35	2.8 ± 0.28	3.4 ± 0.19
**5**	2.3 ± 0.11	2.8 ± 0.23	1.9 ± 0.34	4.8 ± 0.25	3.9 ± 0.41	5.3 ± 0.27
**6**	7.7 ± 0.31	1.8 ± 0.12	4.5 ± 0.43	6.7 ± 0.21	7.1 ± 0.18	8.3 ± 0.29
**8a**	4.5 ± 0.29	3.0 ± 0.19	8.2 ± 0.13	5.9 ± 0.14	6.1 ± 0.38	7.3 ± 0.43
**9b**	21 ± 0.24	57 ± 0.36	14 ± 0.16	92 ± 0.34	81 ± 0.19	179 ± 0.43
**10b**	67 ± 0.21	98 ± 0.35	82 ± 0.14	128 ± 0.35	152 ± 0.49	354 ± 0.46
**11b**	16 ± 0.18	55 ± 0.38	63 ± 0.29	98 ± 0.27	106 ± 0.51	117 ± 0.38
**12a**	22 ± 0.21	89 ± 0.18	28 ± 0.25	112 ± 0.19	101 ± 0.22	149 ± 0.43
**12b**	14 ± 0.37	52 ± 0.26	13 ± 0.06	68 ± 0.29	72 ± 0.34	106 ± 0.23
**13a**	24 ± 0.27	11 ± 0.33	18 ± 0.21	74 ± 0.15	79 ± 0.29	104 ± 0.26
**14**	17 ± 0.34	36 ± 0.42	20 ± 0.12	59 ± 0.21	62 ± 0.24	96 ± 0.17
**15**	3.3 ± 0.11	2.1 ± 0.13	6.6 ± 0.23	17 ± 0.25	19 ± 0.49	31 ± 0.29
**15c**	3.1 ± 0.41	2.3 ± 0.34	15 ± 0.23	16 ± 0.27	14 ± 0.31	27 ± 0.14
**18b**	6.8 ± 0.32	40 ± 0.29	2.1 ± 0.41	13 ± 0.19	11 ± 0.27	47 ± 0.15
**18c**	3.8 ± 0.23	11 ± 0.18	5.3 ± 0.29	14 ± 0.34	12 ± 0.29	51 ± 0.26
**20b**	6.7 ± 0.15	20 ± 0.14	6.1 ± 0.43	12 ± 0.23	13 ± 0.41	49 ± 0.25
**20c**	7.6 ± 0.33	13 ± 0.45	6.8 ± 0.11	19 ± 0.17	17 ± 0.32	54 ± 0.12

^a^ IC_50_ (µM) is the half maximal inhibitory concentration for viable cells. Each IC_50_ (mean ± SE) has been derived from at least three experiments in duplicate.

## Supplementary Materials

The following are available online: ^1^H-NMR and ^13^C-NMR spectra of all new compounds.
