# Supplementary material for: Synthesis and Evaluation of Anticancer Activities of Novel C-28 Guanidine-Functionalized Triterpene Acid Derivatives
Source: Molecules. 2018 Nov 16;23(11):3000. doi: 10.3390/molecules23113000 (PMC6278366; doi:10.3390/molecules23113000)

Supporting Information For

Synthesis and evaluation of anticancer activities of novel C-28 guanidine-functionalized triterpene acid derivatives.

Anna Spivak *, Rezeda Khalitova, Darya Nedopekina, Lilya Dzhemileva *, Milyausha Yunusbaeva, Victor Odinokov, Vladimir D'yakonov and Usein Dzhemilev.

Institute of Petrochemistry and Catalysis, Russian Academy of Sciences, 141 prosp. Oktyabrya, 450075 Ufa, Russian Federation

E-mail:* spivak.ink@gmail.com

Index **Page Number**

^{1}H, ^{13}C NMR of Compound **4** 3

^{1}H, ^{13}C NMR of Compound **5** 5

^{1}H, ^{13}C NMR of Compound **6** 7

^{1}H, ^{13}C NMR of Compound **7** 9

^{1}H, ^{13}C NMR of Compound **8a** 11

^{1}H, ^{13}C NMR of Compound **15** 13

^{1}H, ^{13}C NMR of Compound **18** 15

^{1}H, ^{13}C NMR of Compound **19** 17

^{1}H, ^{13}C NMR of Compound **20** 19

^{1}H, ^{13}C NMR of Compound **21** 21

^{1}H, ^{13}C NMR of Compound **9** 23

^{1}H, ^{13}C NMR of Compound **10** 25

^{1}H, ^{13}C NMR of Compound **11** 27

^{1}H, ^{13}C NMR of Compound **12** 29

^{1}H, ^{13}C NMR of Compound **13** 31

^{1}H, ^{13}C NMR of Compound **15a** 33

^{1}H, ^{13}C NMR of Compound **18a** 35

^{1}H, ^{13}C NMR of Compound **20a** 37

^{1}H, ^{13}C, ^{19}F NMR of Compound **9a** 39

^{1}H, ^{13}C, ^{19}F NMR of Compound **10a** 42

^{1}H, ^{13}C, ^{19}F NMR of Compound **11a** 45

^{1}H, ^{13}C, ^{19}F NMR of Compound **12a** 48

^{1}H, ^{13}C, ^{19}F NMR of Compound **13a** 51

^{1}H, ^{13}C, ^{19}F NMR of Compound **15b** 54

^{1}H, ^{13}C, ^{19}F NMR of Compound **18b** 57

^{1}H, ^{13}C, ^{19}F NMR of Compound **20b** 60

^{1}H, ^{13}C NMR of Compound **9b** 63

^{1}H, ^{13}C NMR of Compound **10b** 65

^{1}H, ^{13}C NMR of Compound **11b** 67

^{1}H, ^{13}C NMR of Compound **12b** 69

^{1}H, ^{13}C NMR of Compound **15c** 71

^{1}H, ^{13}C NMR of Compound **18c** 73

^{1}H, ^{13}C NMR of Compound **20c** 75

^{1}H, ^{13}C NMR of Compound **14** 77

N-(4-aminobuthyl)-3-oxolupane-28-amide (4). ^{1}H NMR spectra ($CDCl_3$)

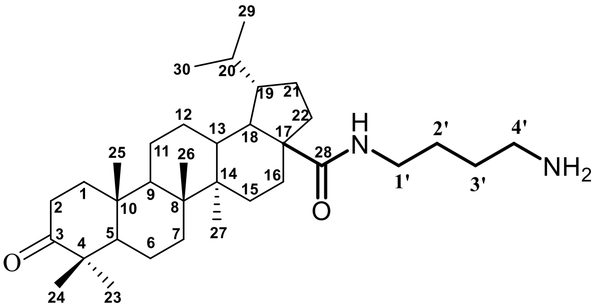

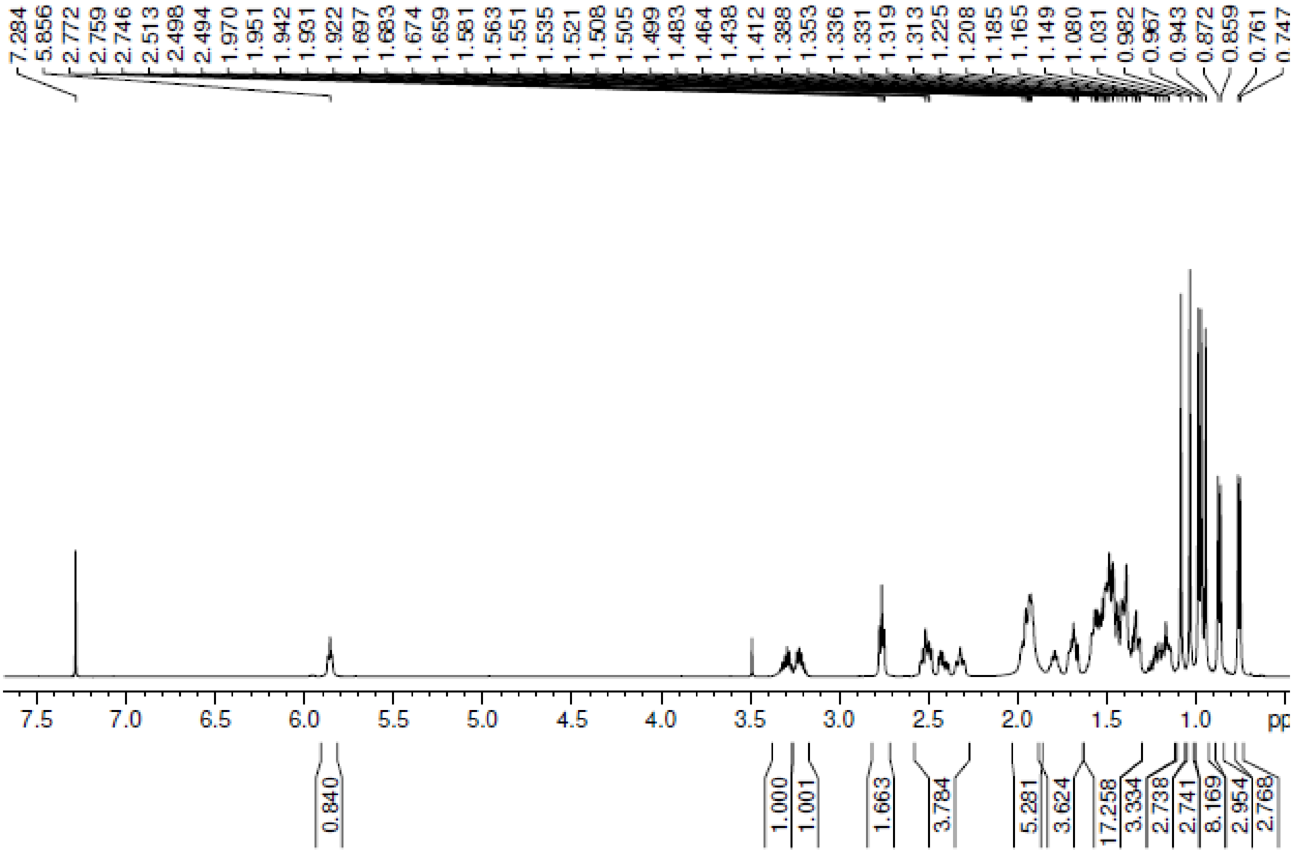

N-(4-aminobuthyl)-3-oxolupane-28-amide (4). ^{13}C NMR spectra ($CDCl_3$)

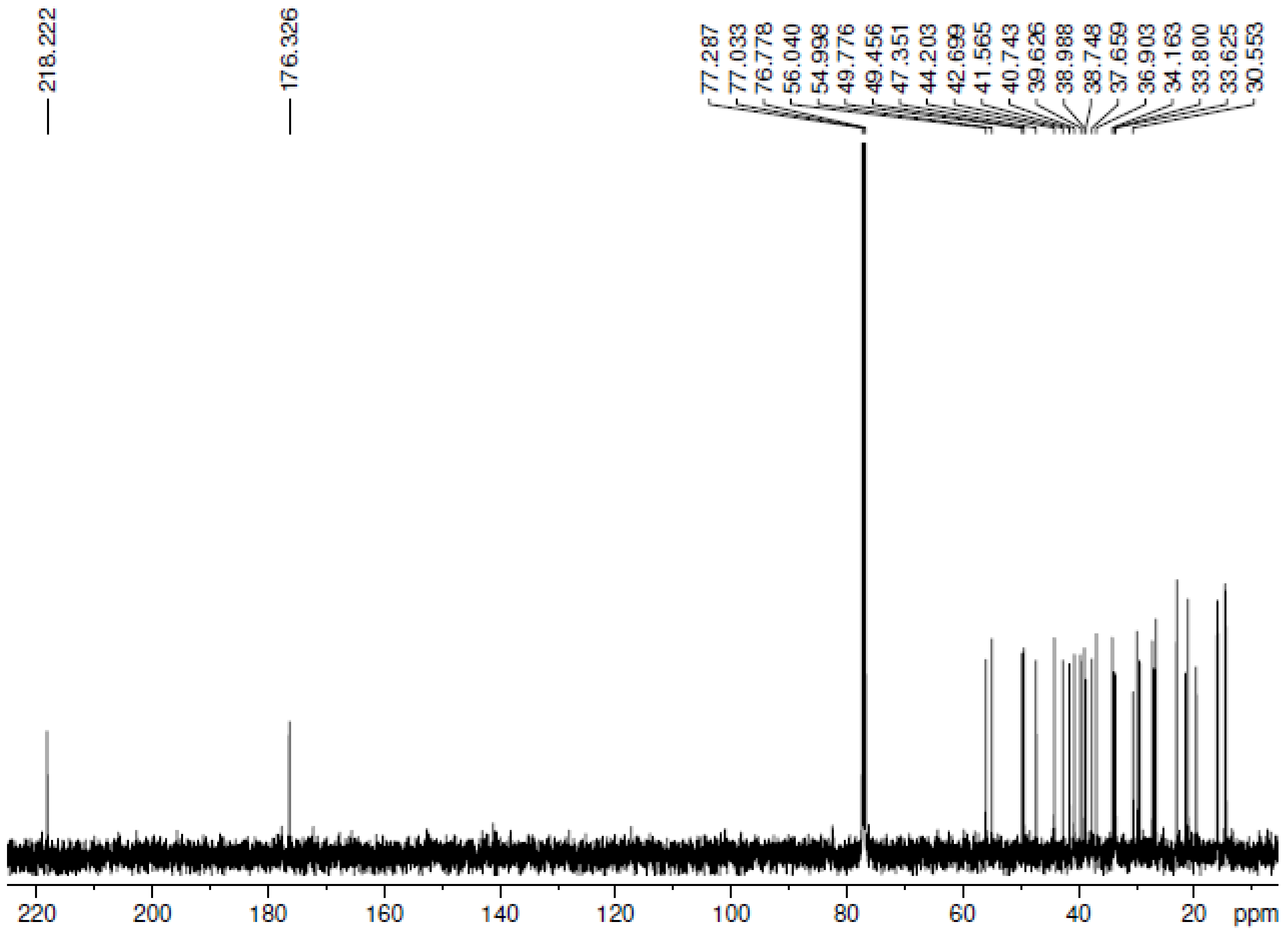

3β-N-(2-aminoethyl)-3-O-acetyl-lupane-28-amide (5). ^{1}H NMR spectra ($CDCl_3$)

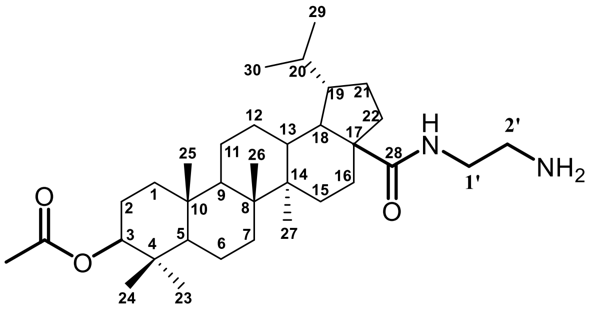

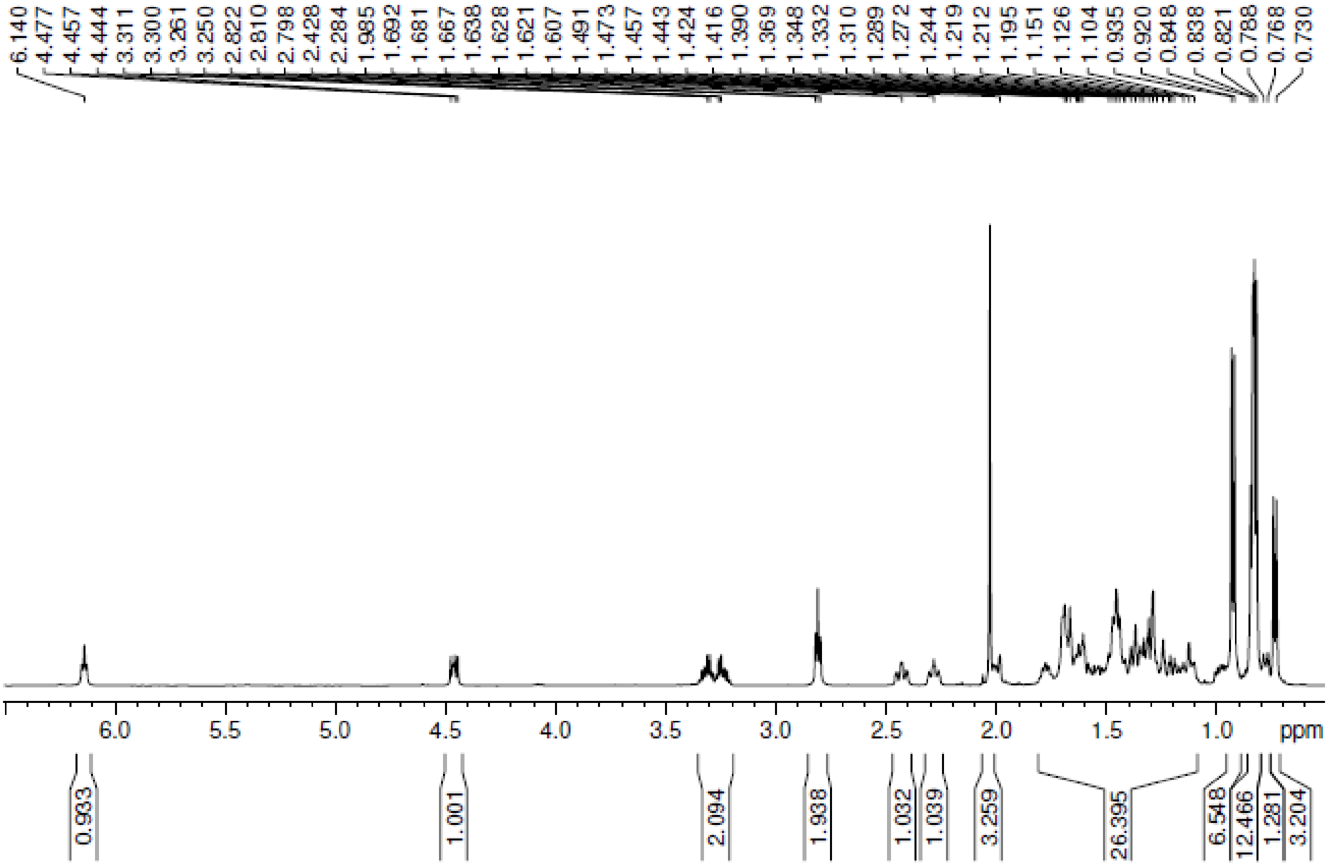

3β-N-(2-aminoethyl)-3-O-acetyl-lupane-28-amide (5). ^{13}C NMR spectra ($CDCl_3$)

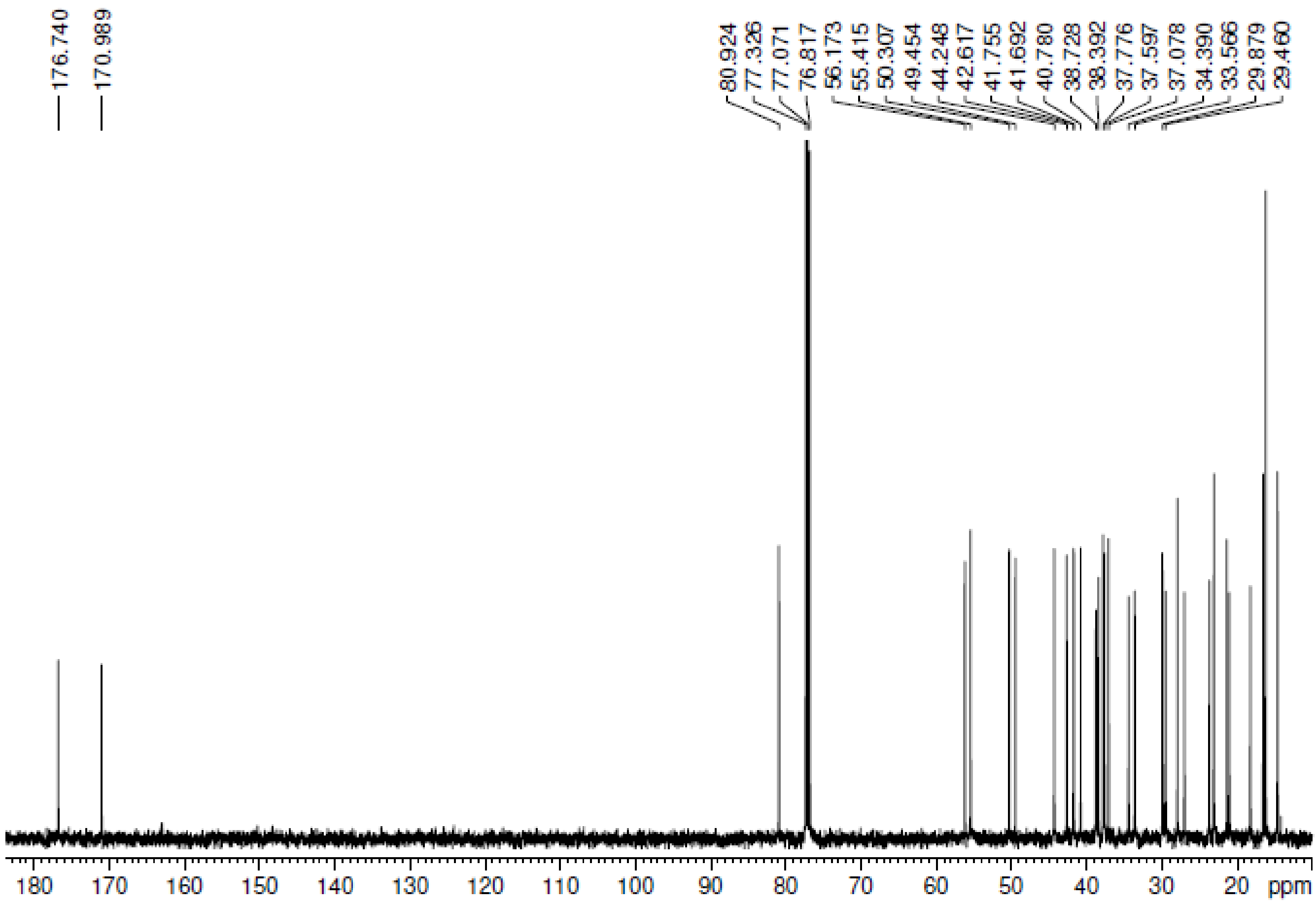

3β-N-(4-aminobuthyl)-3-O-acetyl-lupane-28-amide (6) ^{1}H NMR spectra ($CDCl_3$)

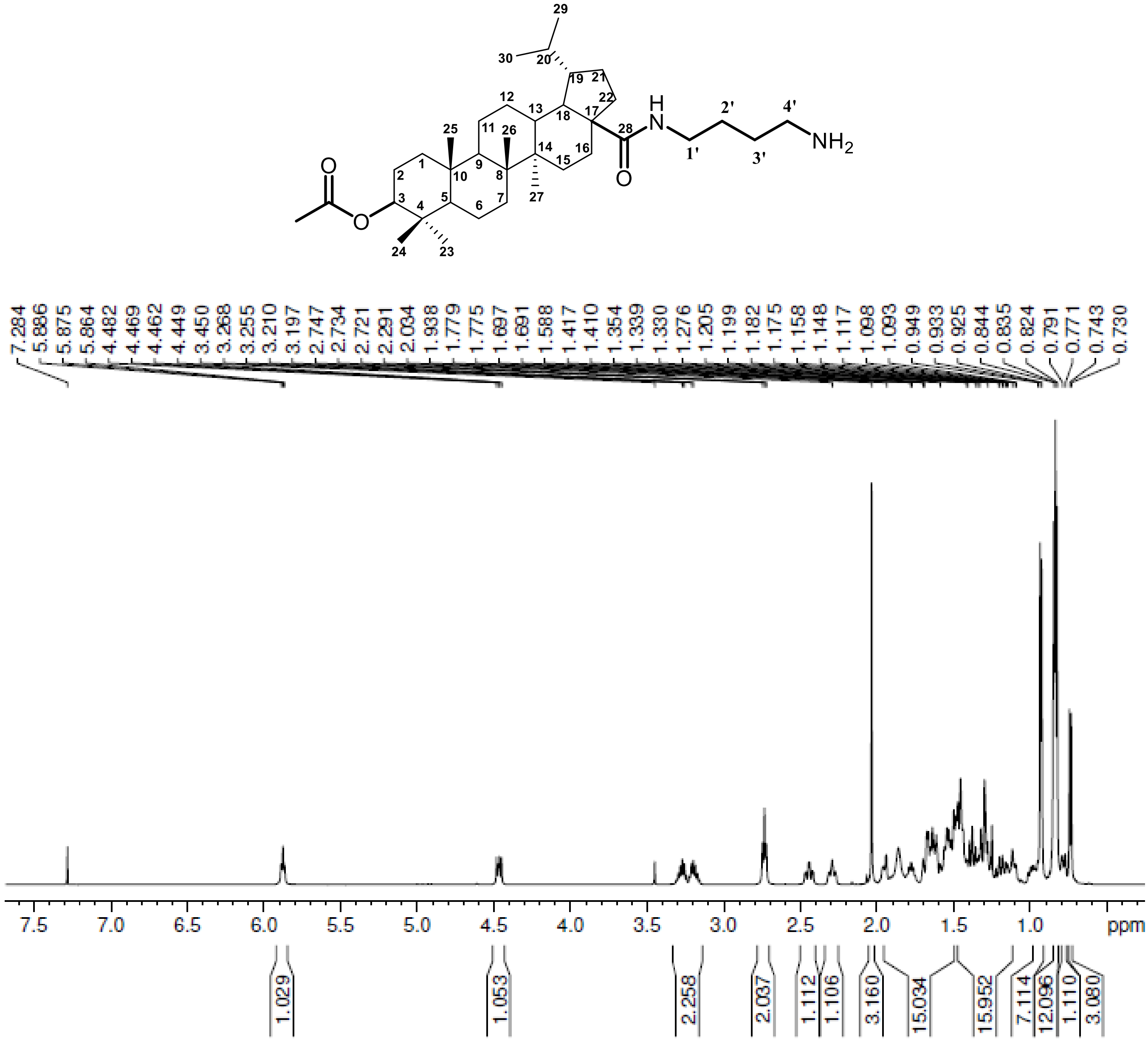

3β-N-(4-aminobuthyl)-3-O-acetyl-lupane-28-amide (6) ^{13}C NMR spectra ($CDCl_3$)

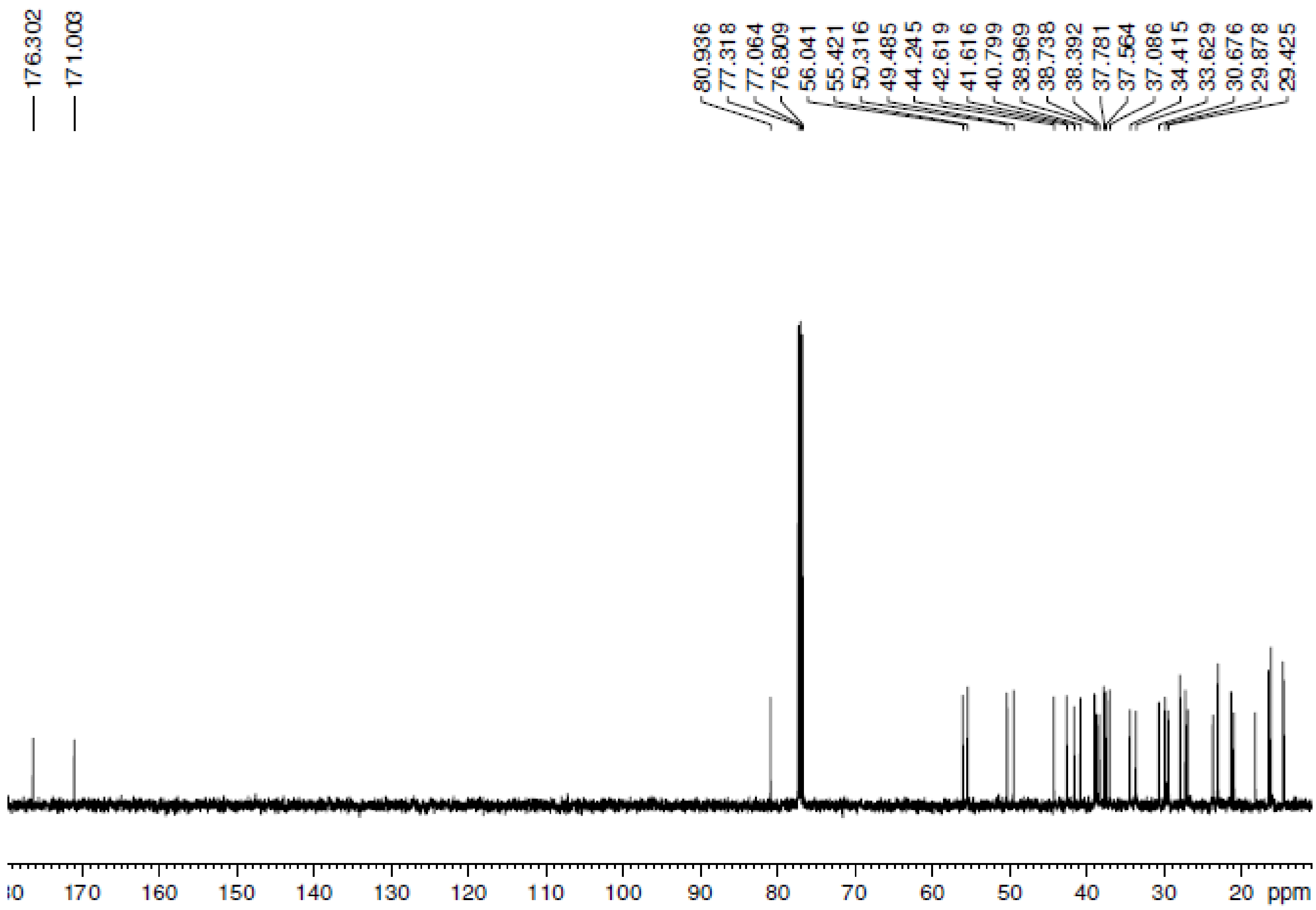

3β-N-[2-(N,N'-bis-aminoethyl)-aminoethyl]-3-O-acetyl-lupane-28-amide (7).

^{1}H NMR spectra (MeOD)

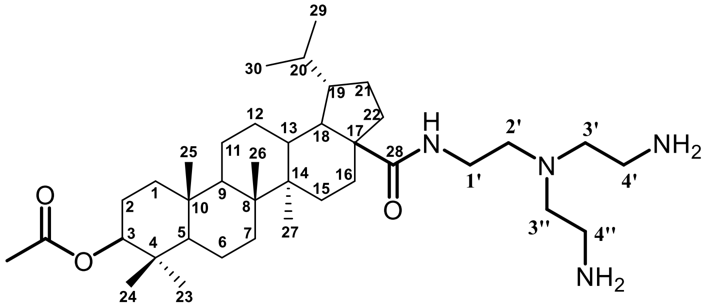

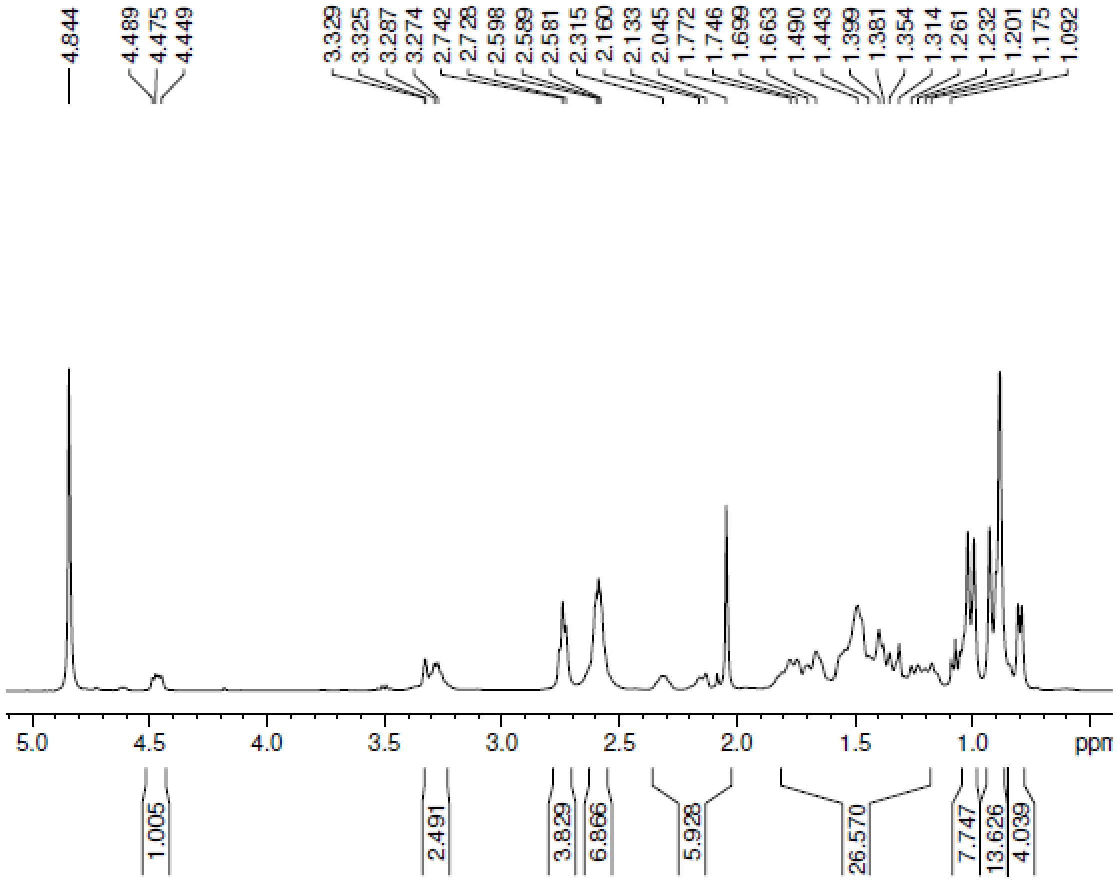

3β-N-[2-(N,N’-bis-aminoethyl)-aminoethyl]-3-O-acetyl-lupane-28-amide (7).

^{13}C NMR spectra (MeOD)

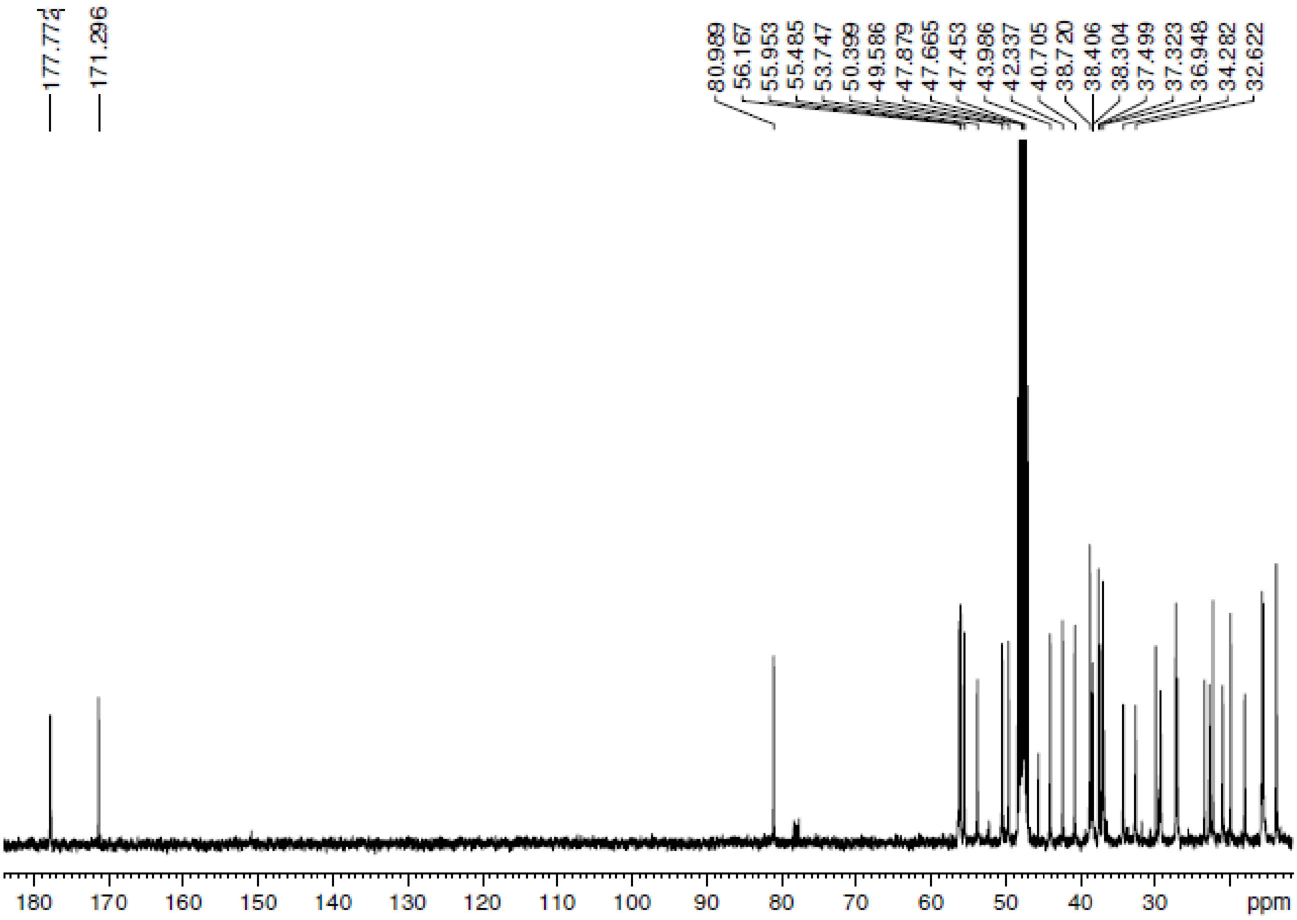

3β-N-{[3-(3-aminopropyl)piperazinyl]propyl}-3-O-acetyl-lupane-28-amide (8a).

^{1}H NMR spectra ($CDCl_3$)

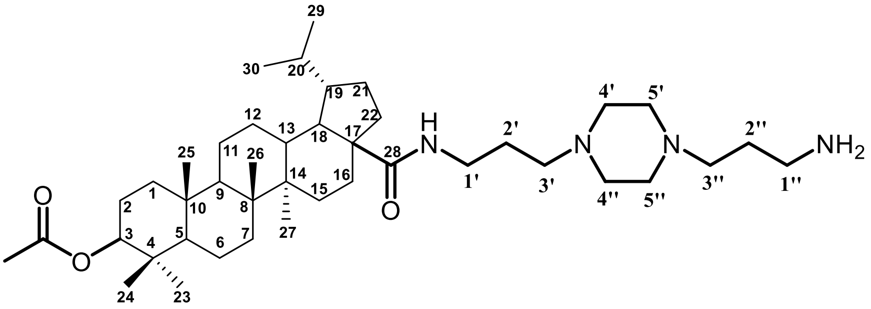

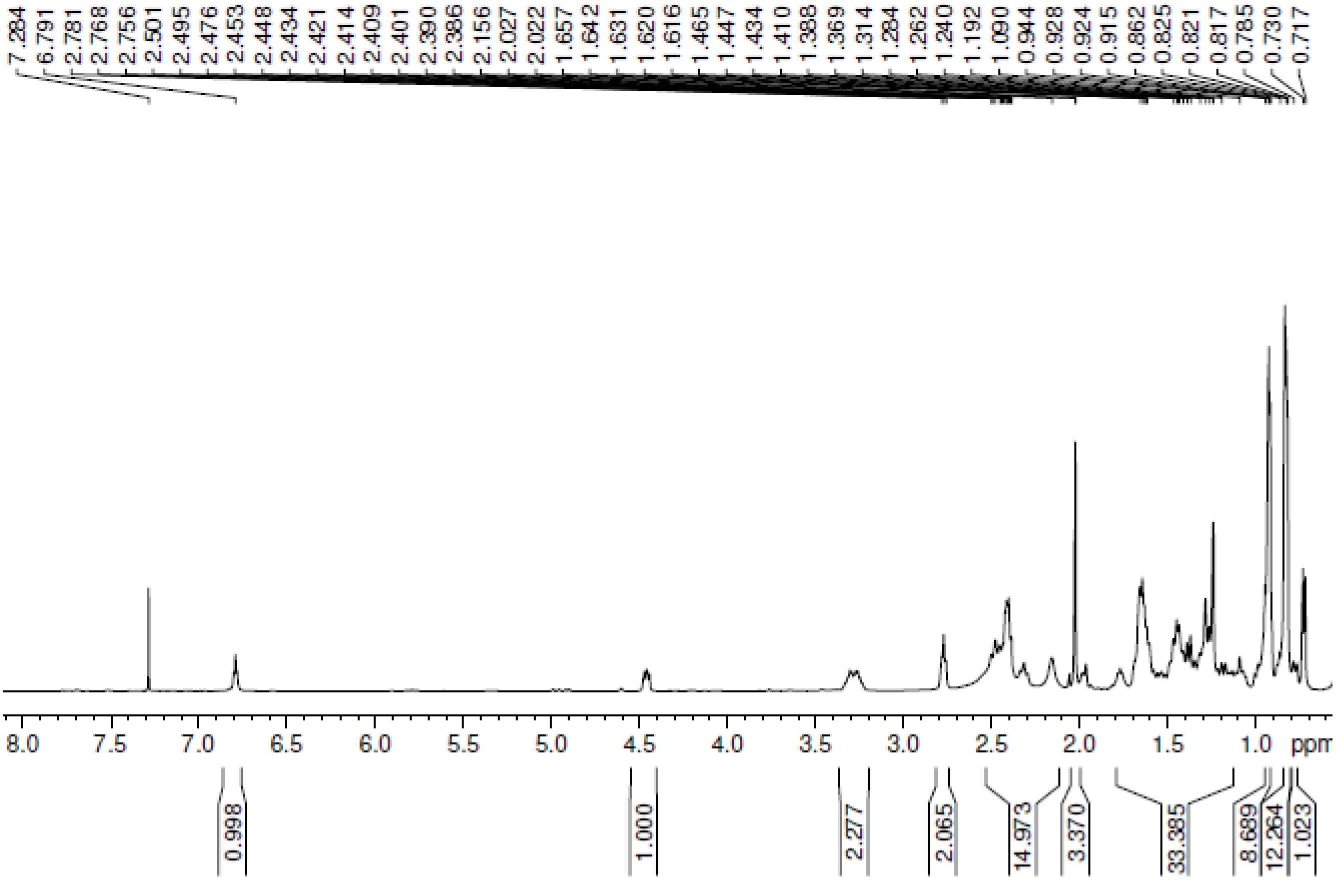

3β-N-{[3-(3-aminopropyl)piperazinyl]propyl}-3-O-acetyl-lupane-28-amide (8a).

^{13}C NMR spectra ($CDCl_3$)

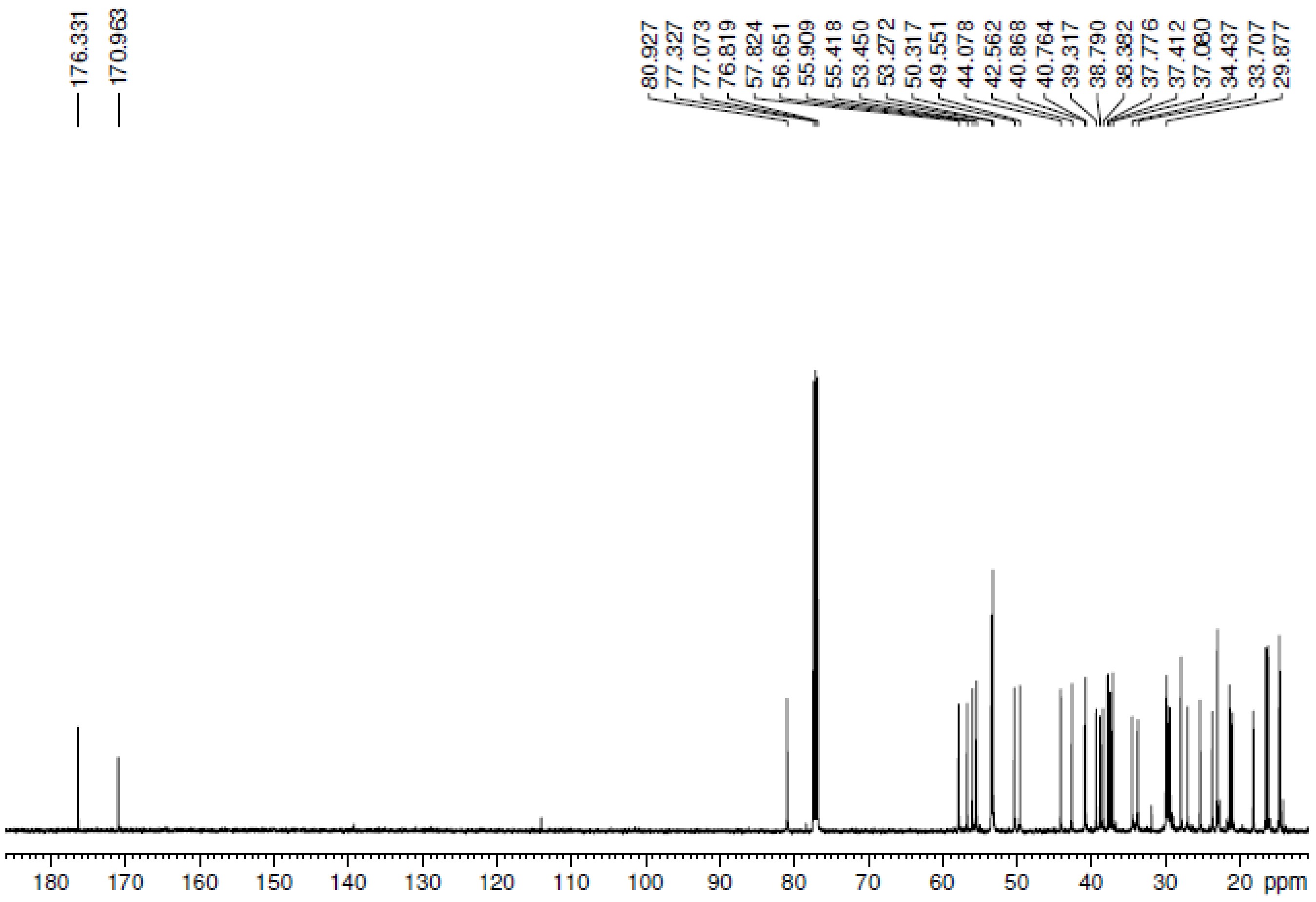

3β-[2-amino-3-hydroxy-2-(hydroxymethyl)propyl]-3-O-acetyl -lupane-28-oate (15).

^{1}H NMR spectra ($CDCl_3$)

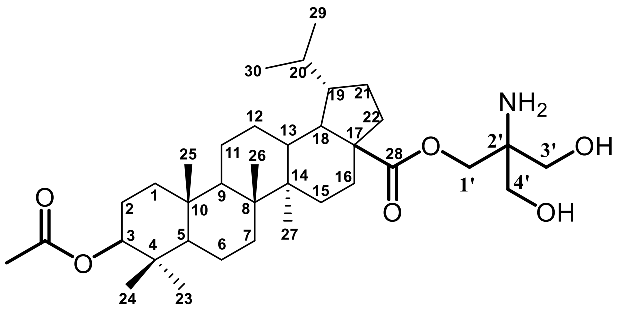

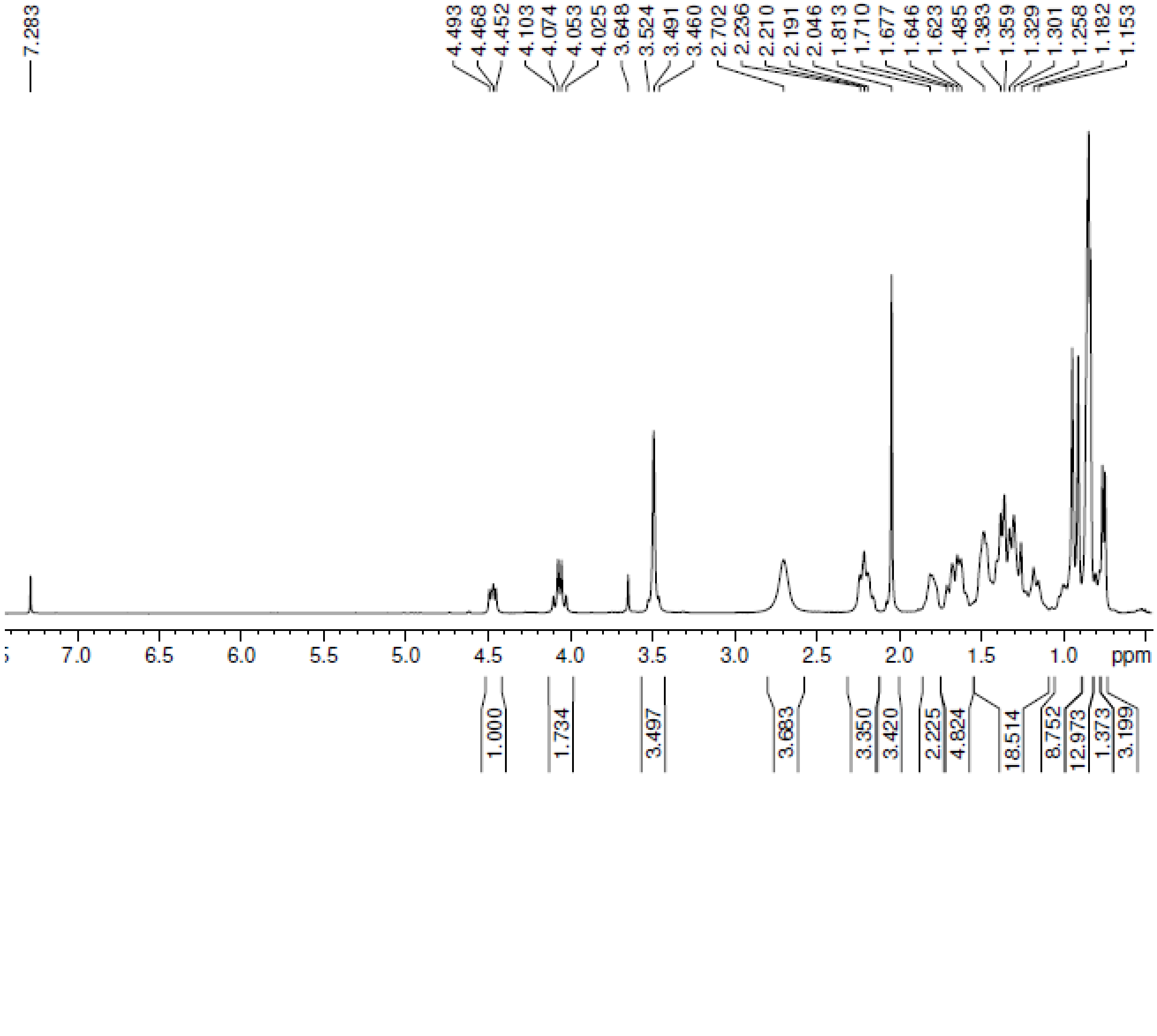

3β-[2-amino-3-hydroxy-2-(hydroxymethyl)propyl]-3-O-acetyl -lupane-28-oate (15).

^{13}C NMR spectra ($CDCl_3$)

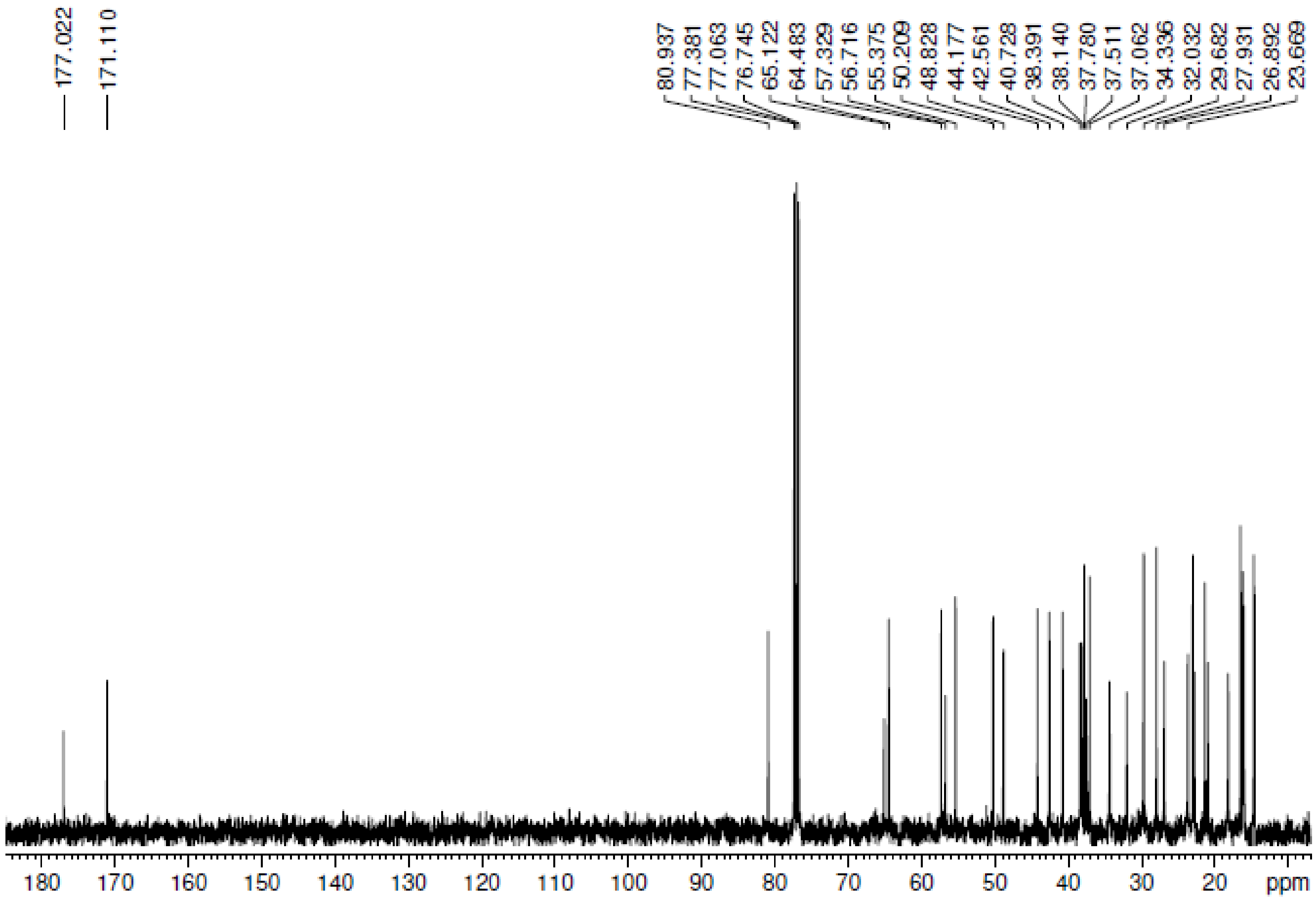

3β-[2-amino-3-hydroxy-2-(hydroxymethyl)propyl]-3-O-acetylurs-12-en-28-oate (18).

^{1}H NMR spectra ($CDCl_3$)

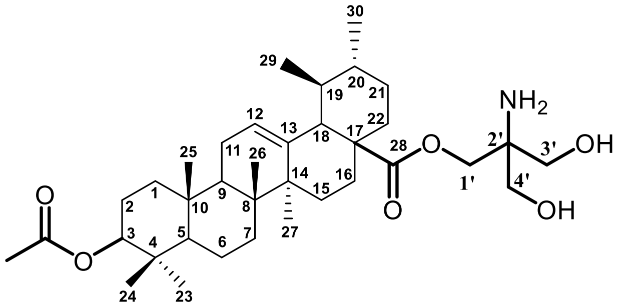

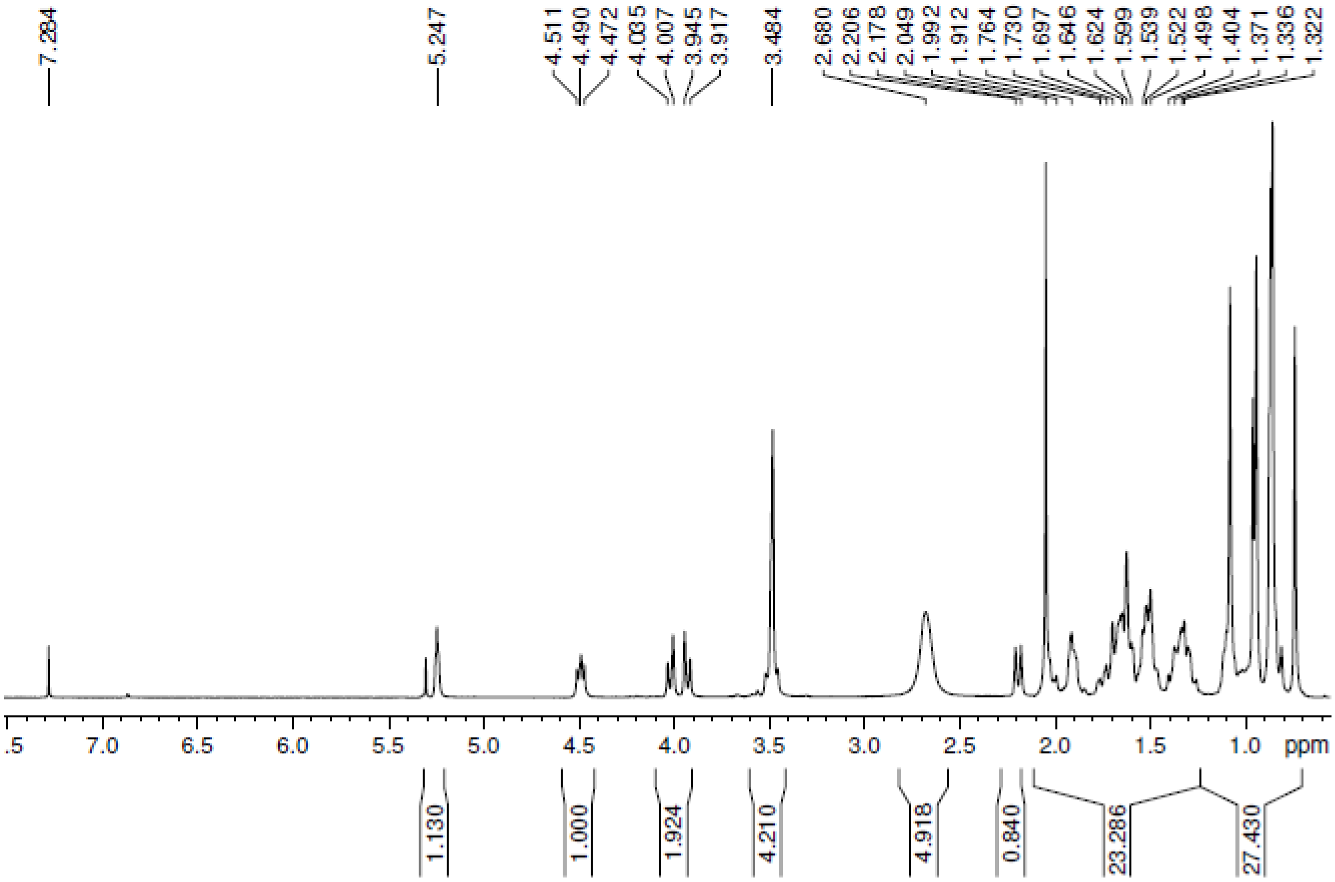

3β-[2-amino-3-hydroxy-2-(hydroxymethyl)propyl]-3-O-acetylurs-12-en-28-oate (18).

^{13}C NMR spectra ($CDCl_3$)

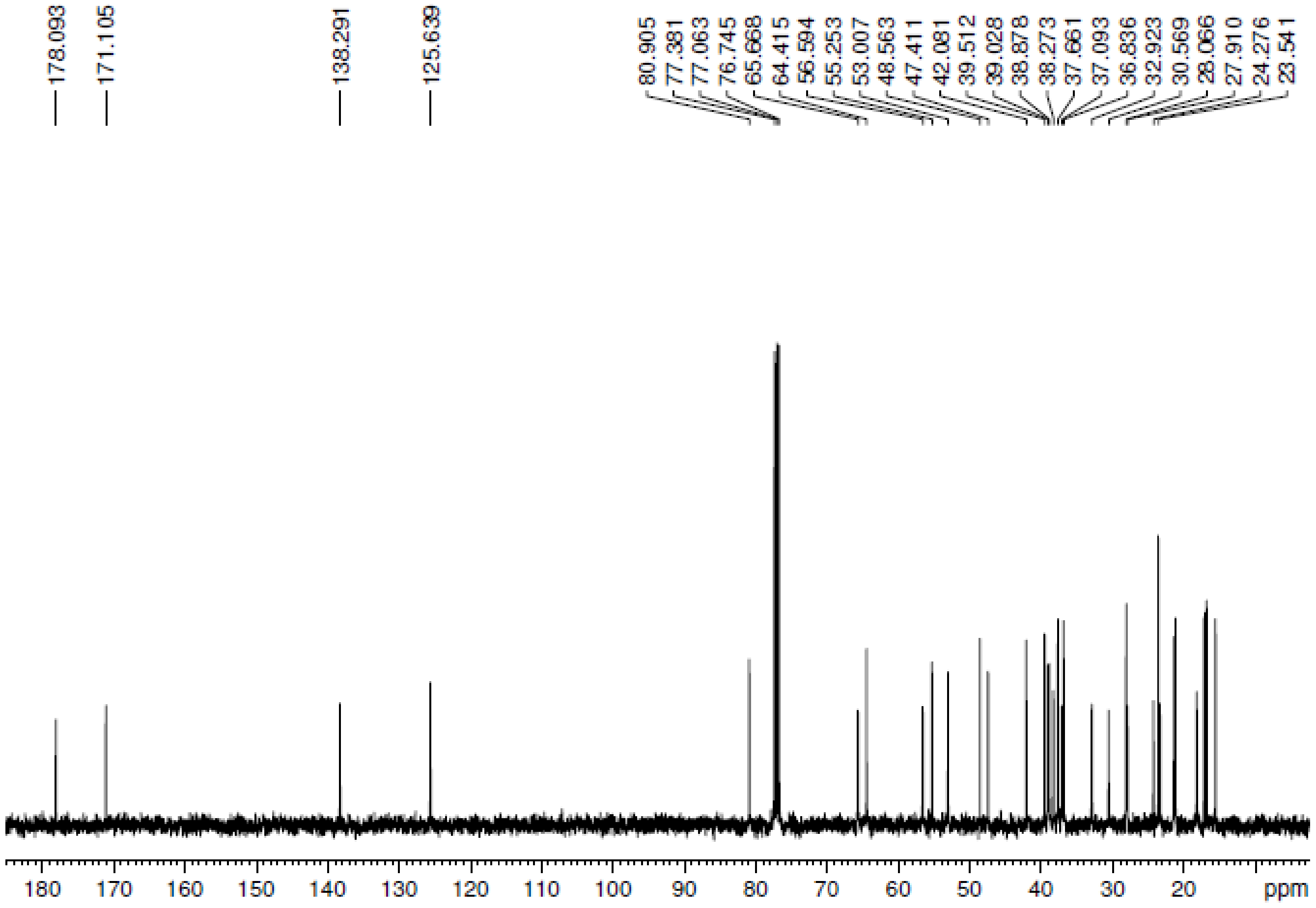

3β-N-[(1',1',1'-tris-hidroxymethyl)methyl]-3-O-acetyl-ursolamide (19)

^{1}H NMR spectra (MeOD)

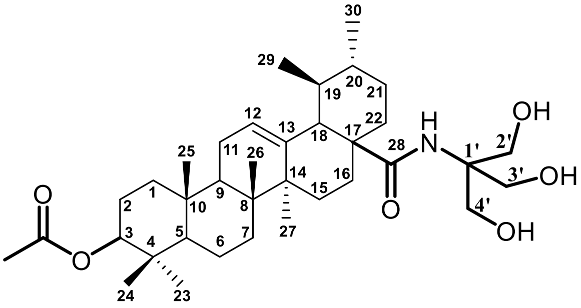

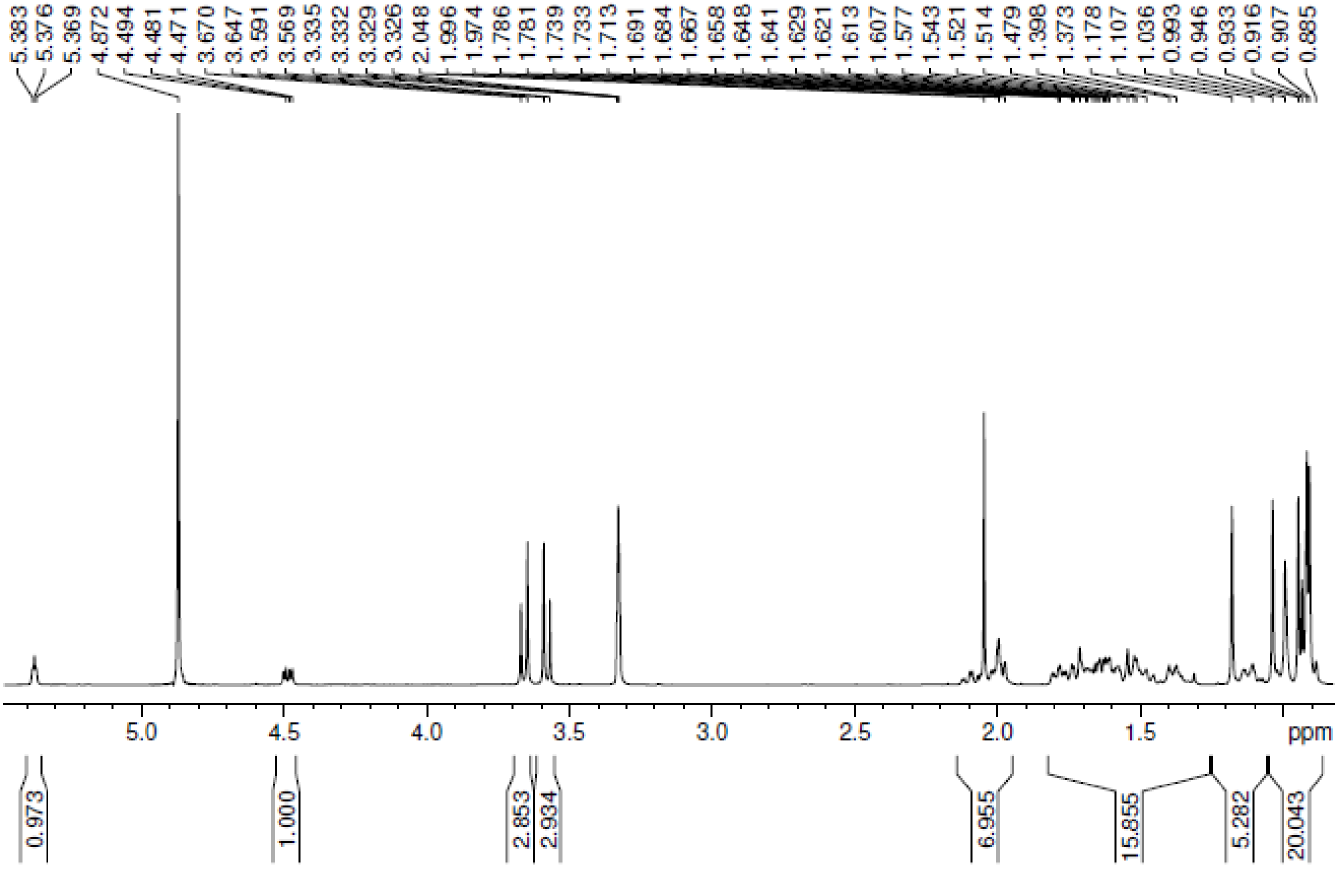

3β-N-[(1',1',1'-tris-hidroxymethyl)methyl]-3-O-acetyl-ursolamide (19)

^{13}C NMR spectra (d5-DMSO)

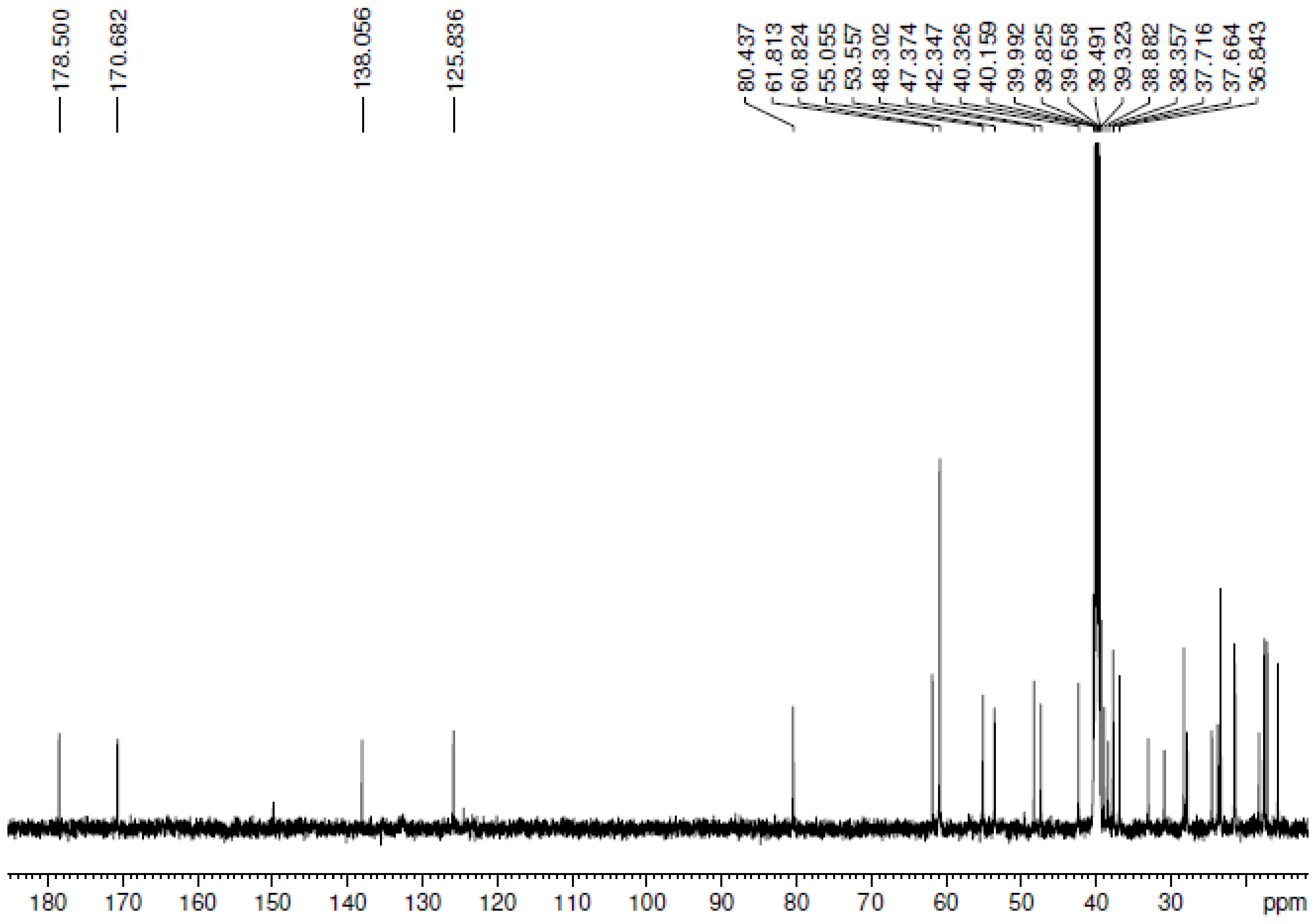

3β-[2-amino-3-hydroxy-2(hydroxymethyl)propyl]-3-O-acetylolean-12-en-28-oate (20).

^{1}H NMR spectra ($CDCl_3$)

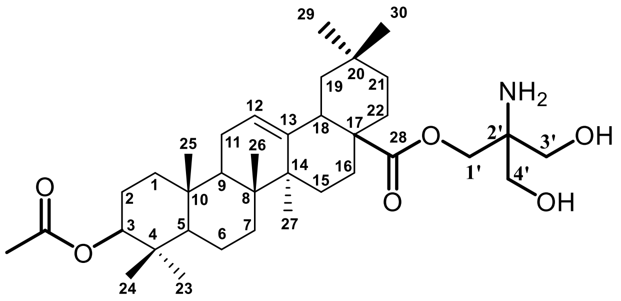

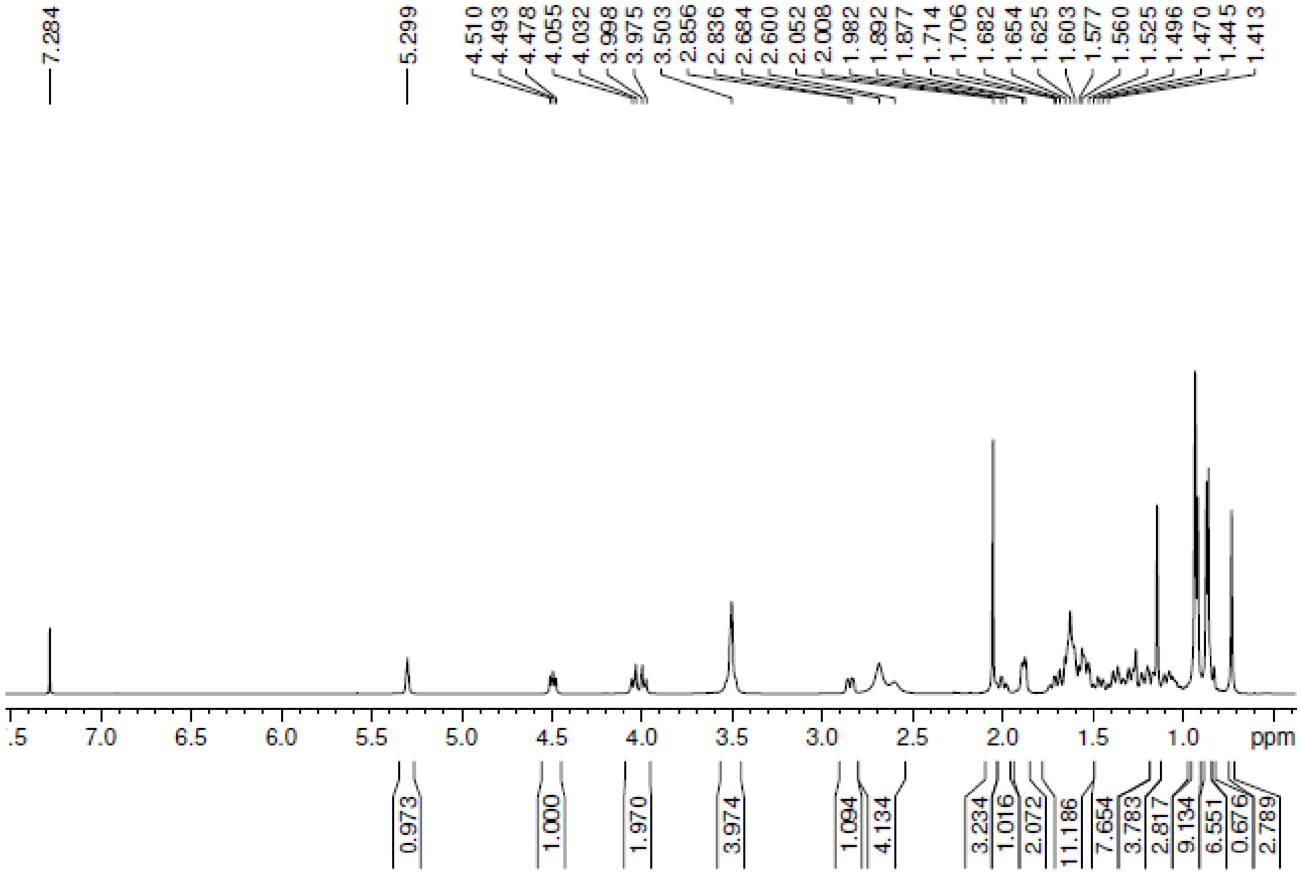

3β-[2-amino-3-hydroxy-2(hydroxymethyl)propyl]-3-O-acetylolean-12-en-28-oate (20).

^{13}C NMR spectra ($CDCl_3$)

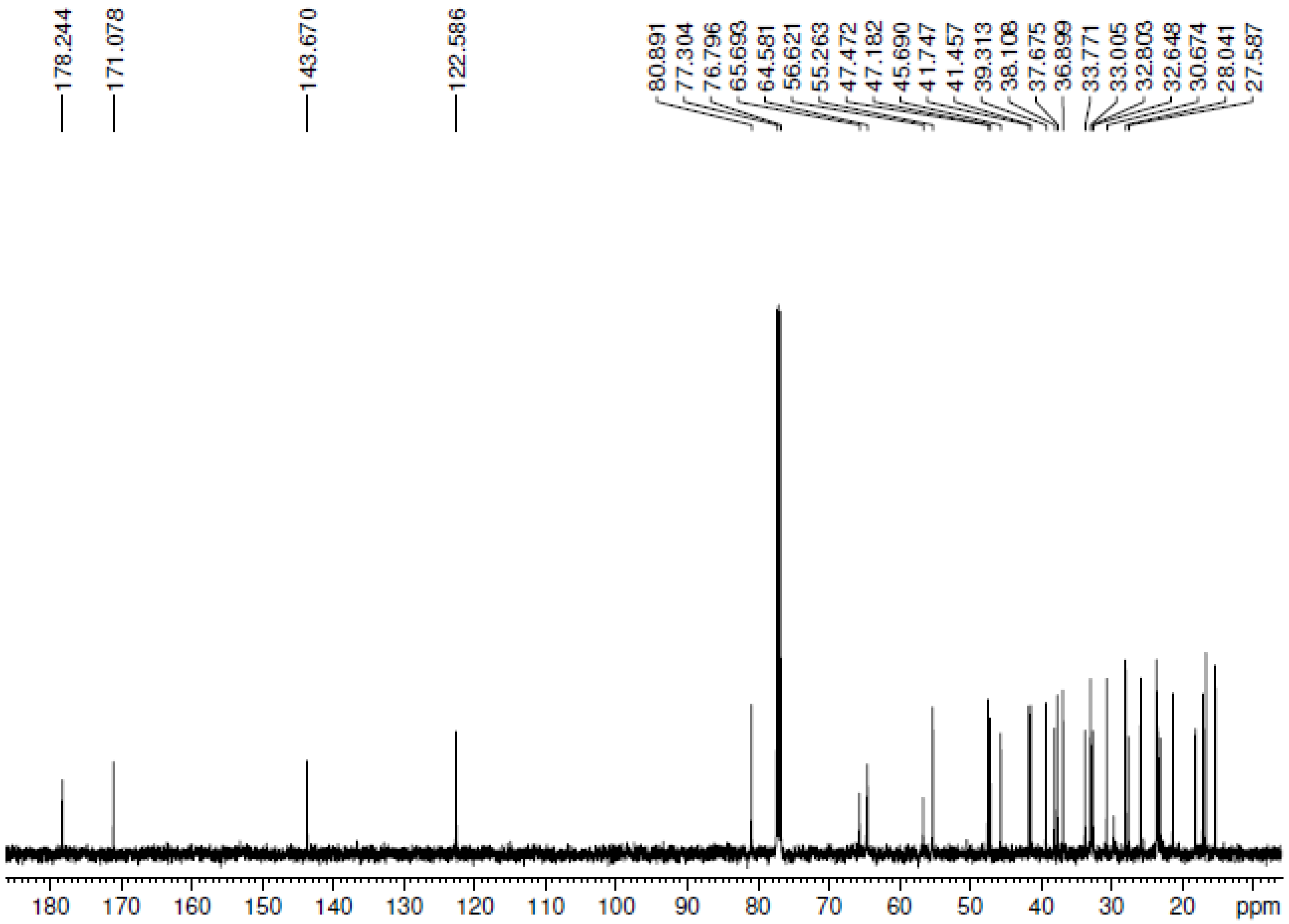

3β-N-[(1',1',1'-tris-hidroxymethyl)methyl]-3-O-acetyl-oleanolamide (21)

^{1}H NMR spectra (d$_6$-DMSO)

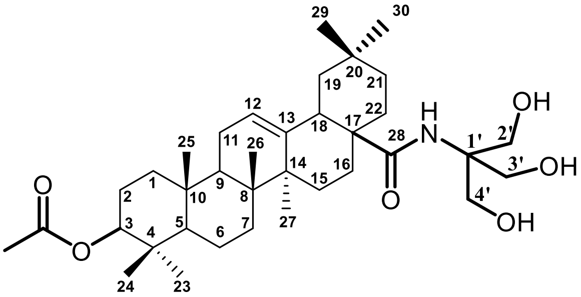

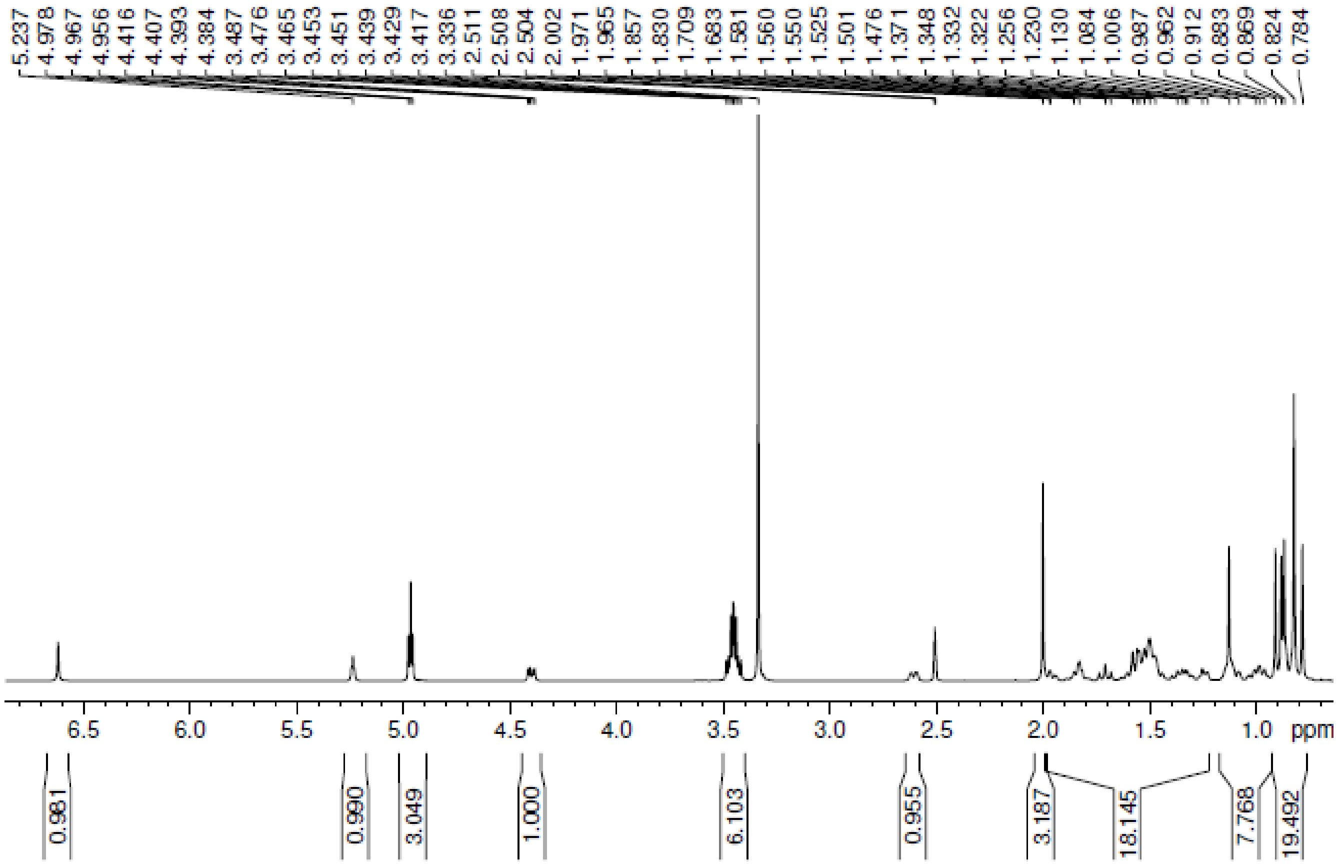

3β-N-[(1',1',1'-tris-hidroxymethyl)methyl]-3-O-acetyl-oleanolamide (21)

^{13}C NMR spectra (d_6-DMSO)

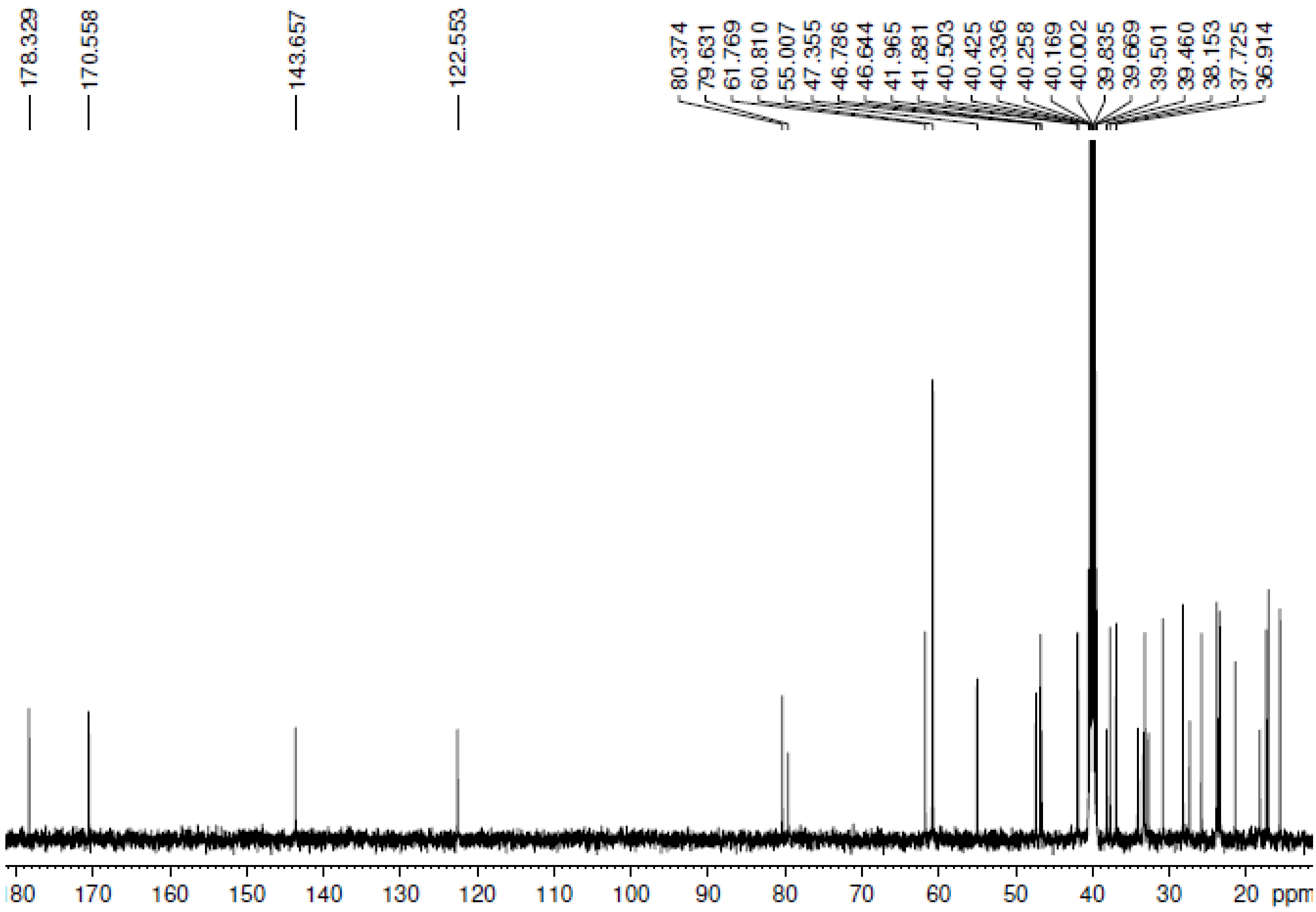

N-[4-tert-butyloxycarbonyl buthylguanidine]-3-oxo-lupane-28-amide (9)

^{1}H NMR spectra ($CDCl_3$)

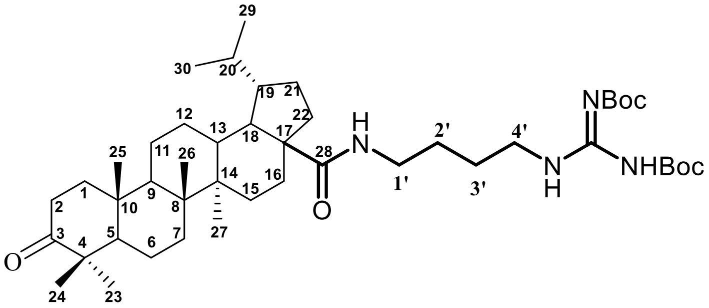

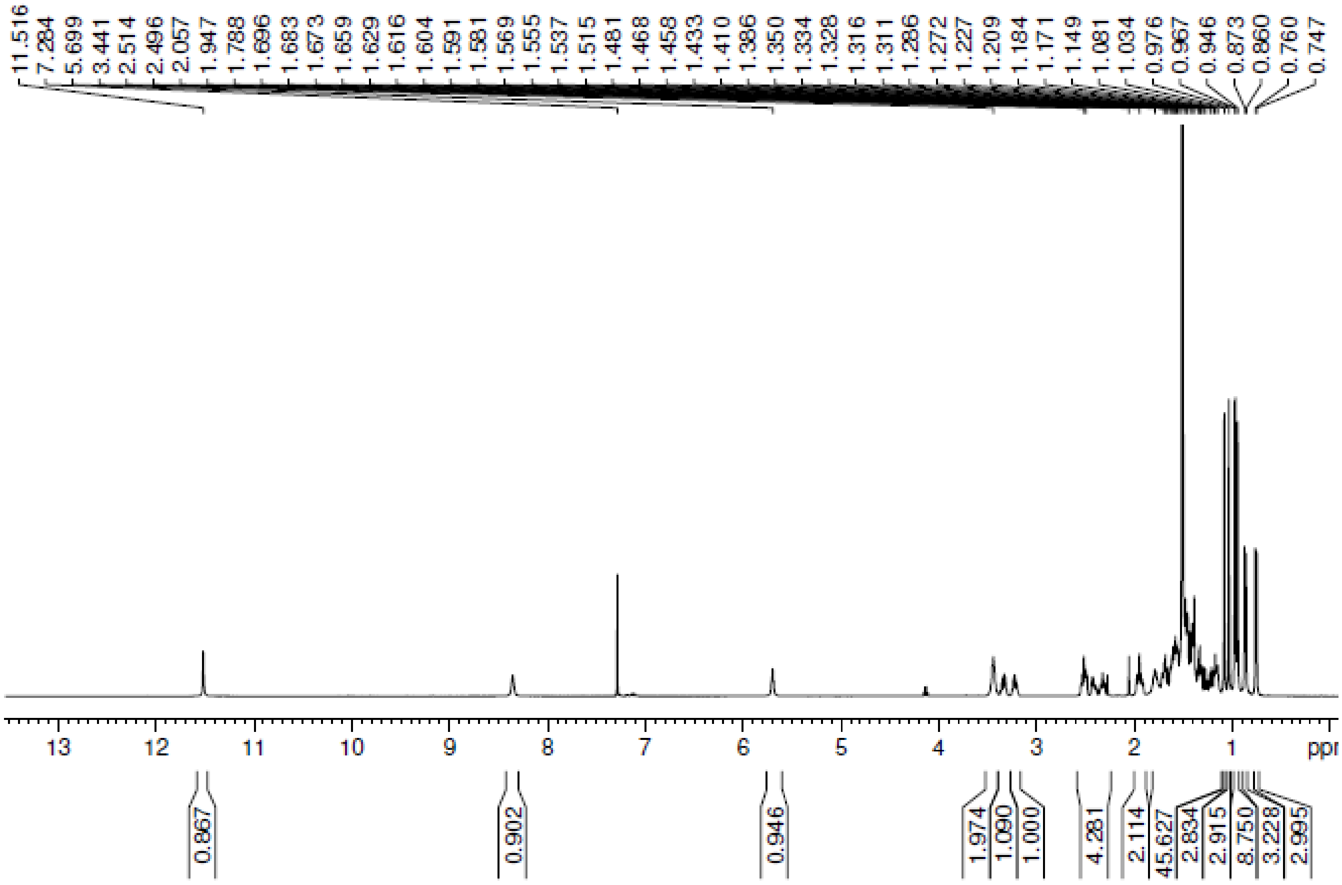

N-[4-tert-butyloxycarbonyl buthylguanidine]-3-oxo-lupane-28-amide (9)

^{13}C NMR spectra ($CDCl_3$)

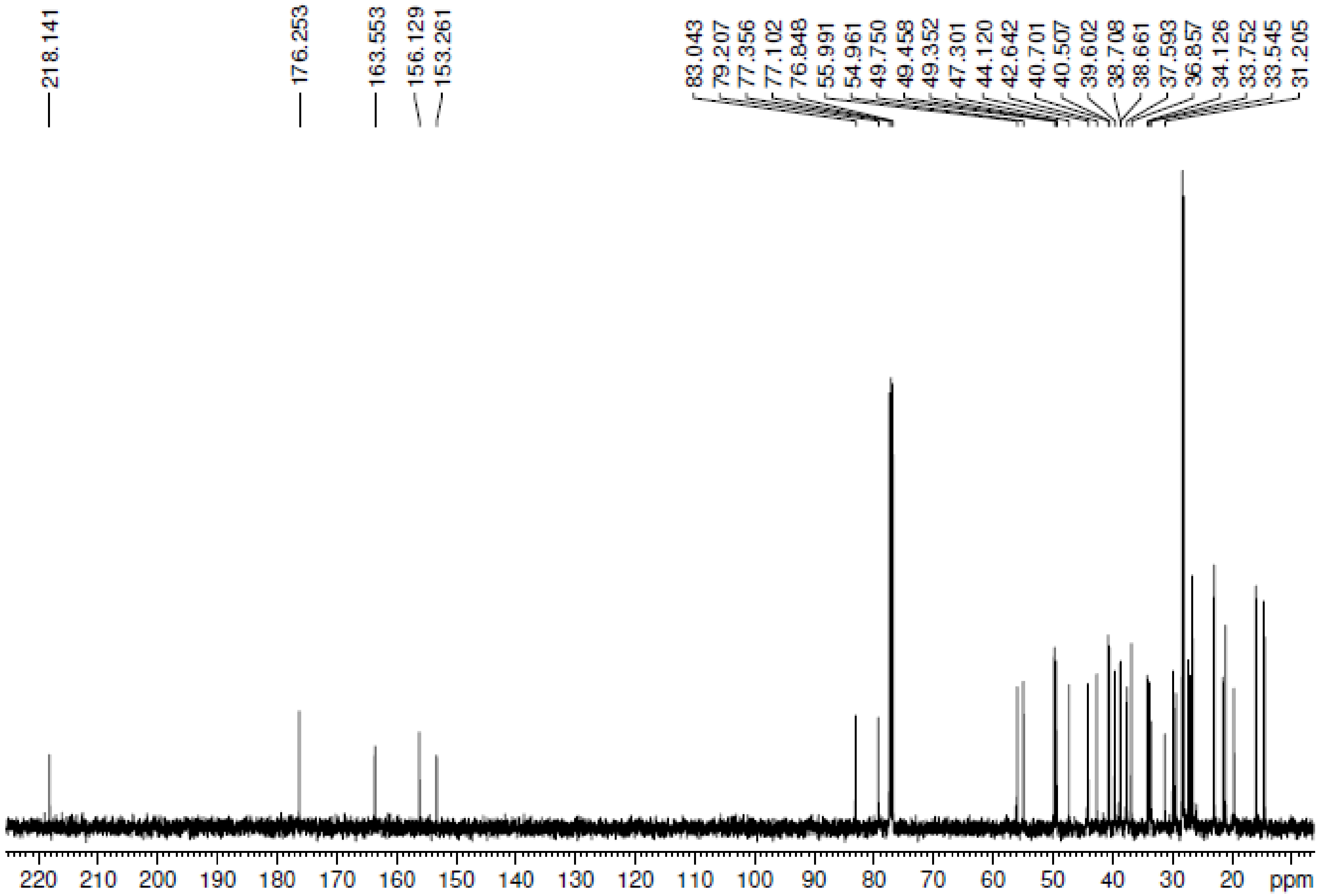

3β-N-(2-tert-butyloxycarbonyl ethylguanidine)-3-O-acetyl-lupane-28-amide (10).

^{1}H NMR spectra ($CDCl_3$)

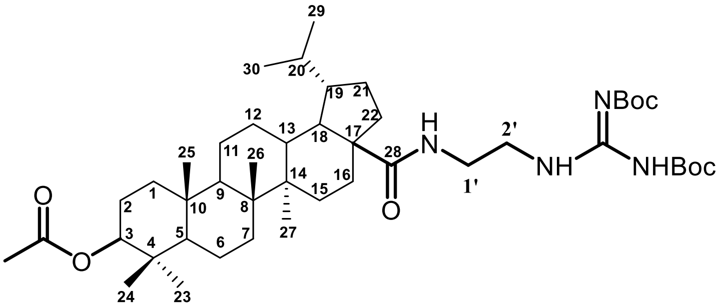

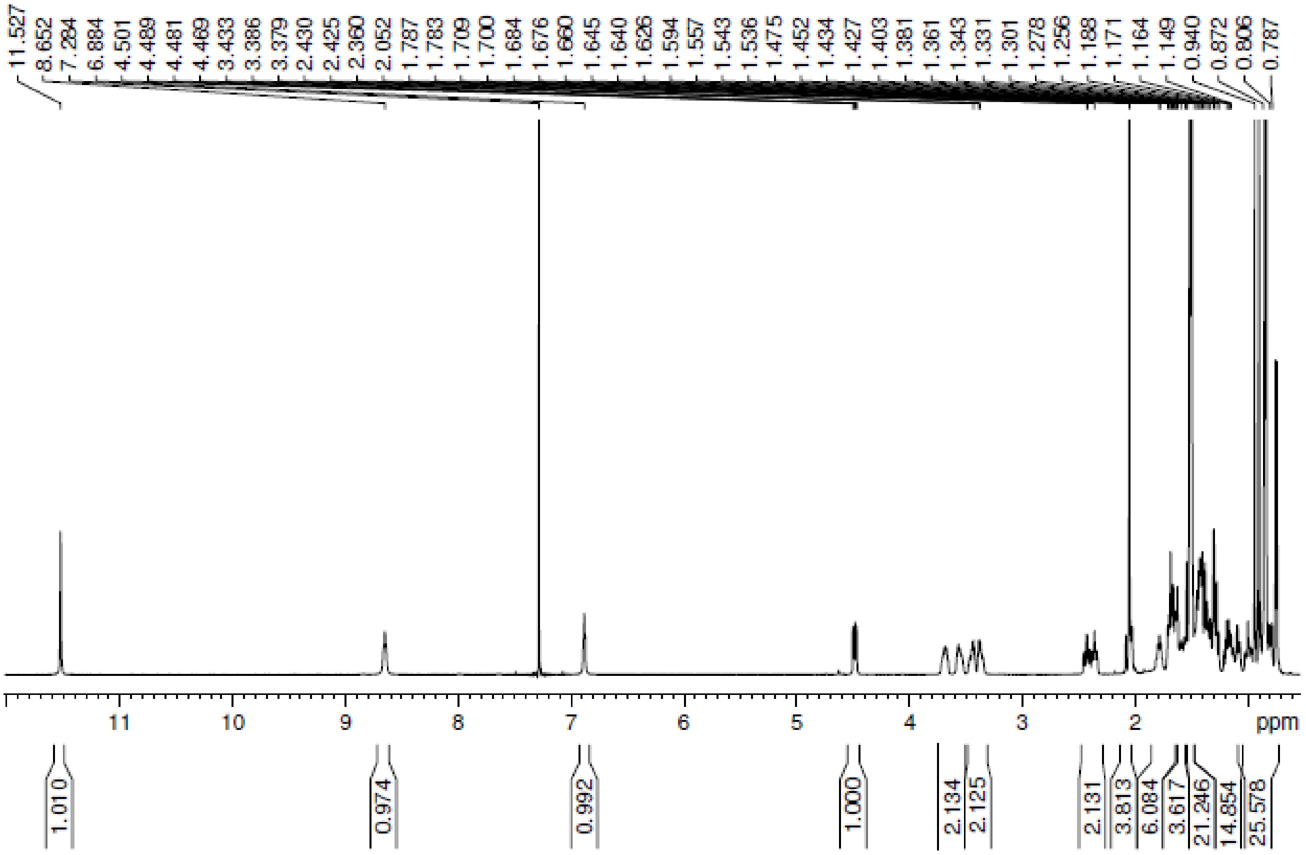

3β-N-(2-tert-butyloxycarbonyl ethylguanidine)-3-O-acetyl-lupane-28-amide (10).

^{13}C NMR spectra ($CDCl_3$)

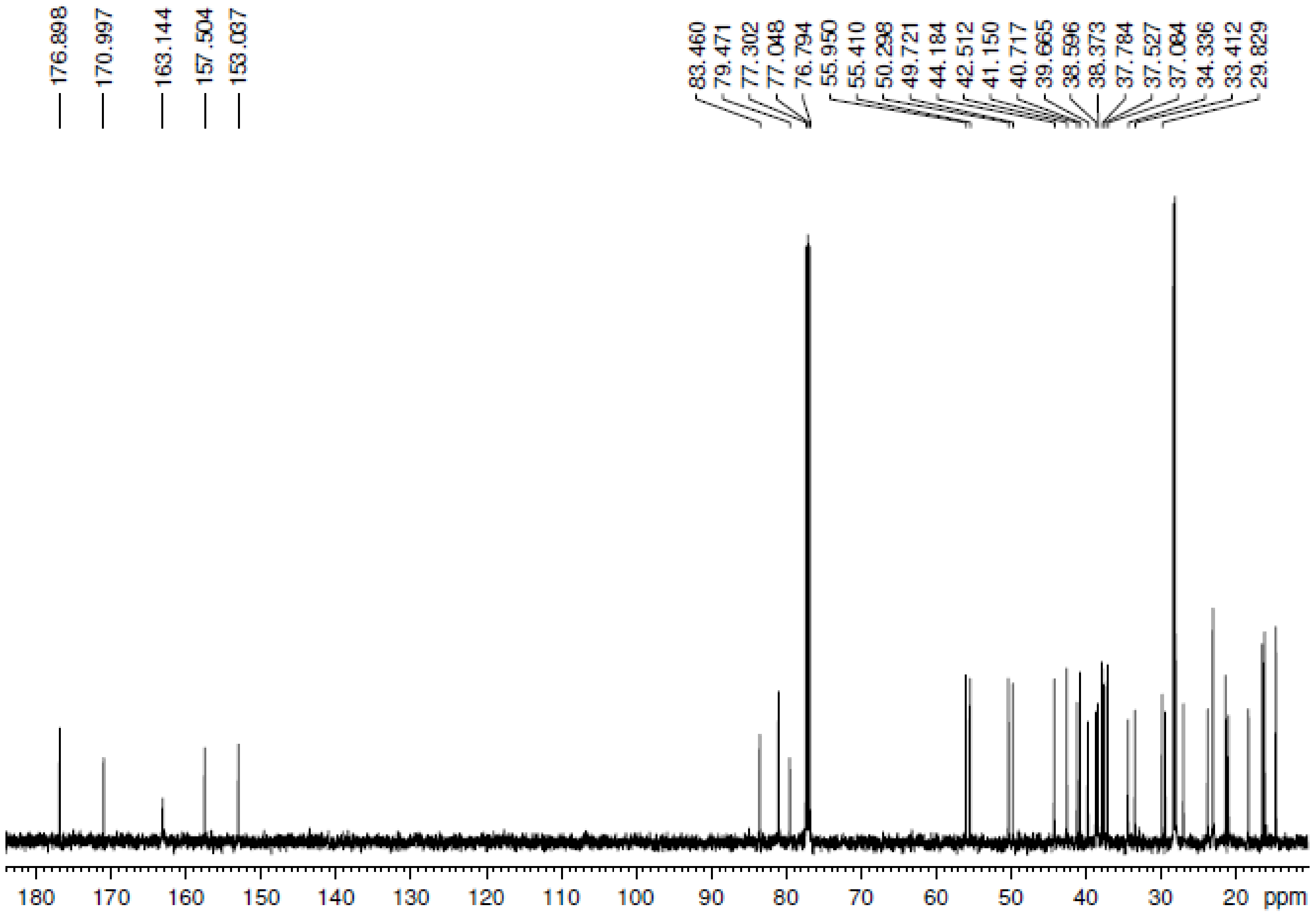

3β-N-[4-tert-butyloxycarbonyl buthylguanidine]-3-O-acetyl -lupane-28-amide (11).

^{1}H NMR spectra ($CDCl_3$)

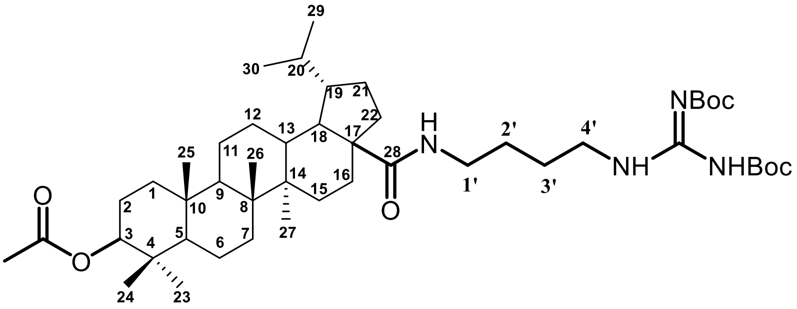

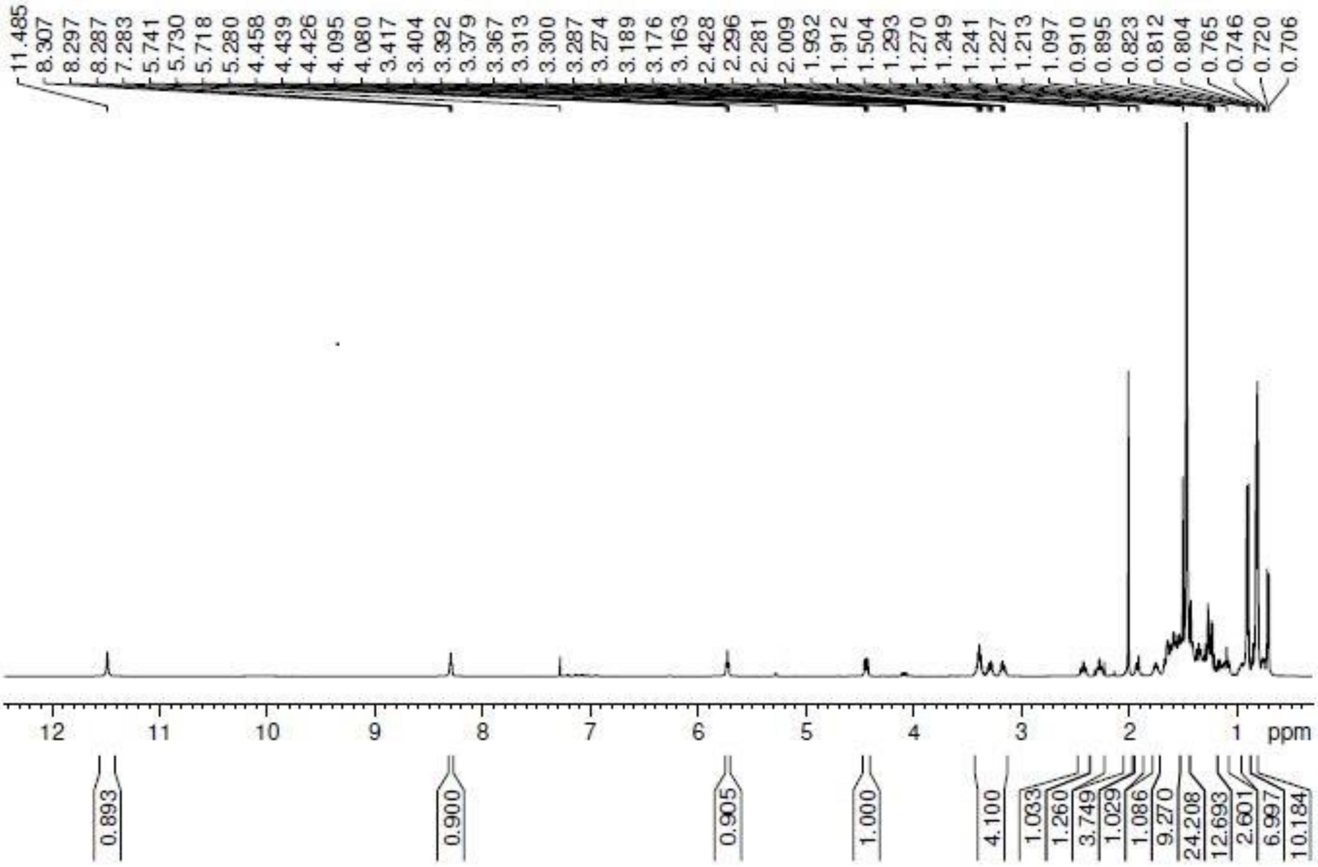

3β-N-[4-tert-butyloxycarbonyl buthylguanidine]-3-O-acetyl -lupane-28-amide (11).

^{13}C NMR spectra ($CDCl_3$)

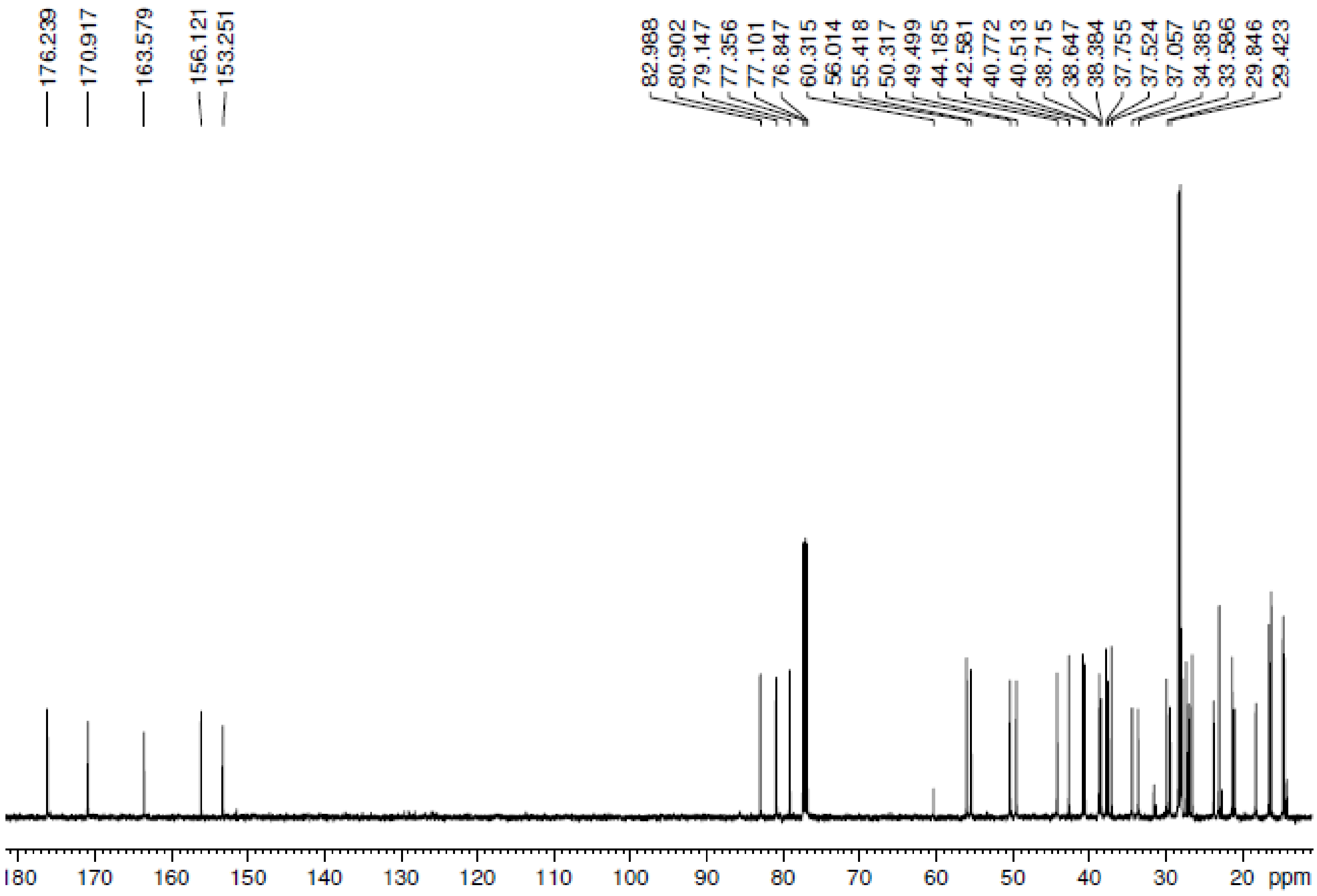

3β-N-[2-(N,N’-bis-tert-butyloxycarbonyl ethylgyanidine)-aminoethyl]-3-O-acetyl-lupane-28-amide (12). ^{1}H NMR spectra ($CDCl_3$)

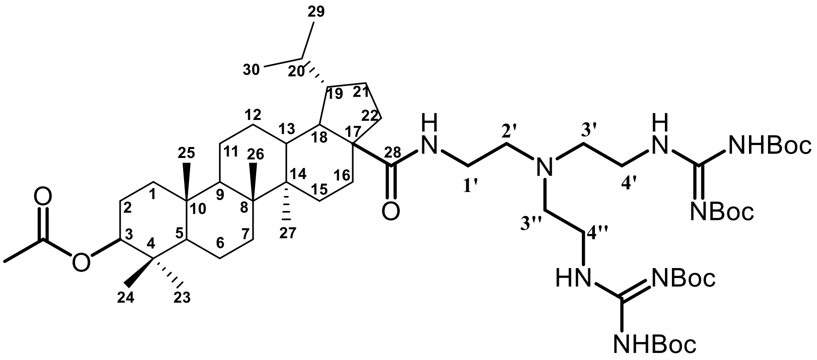

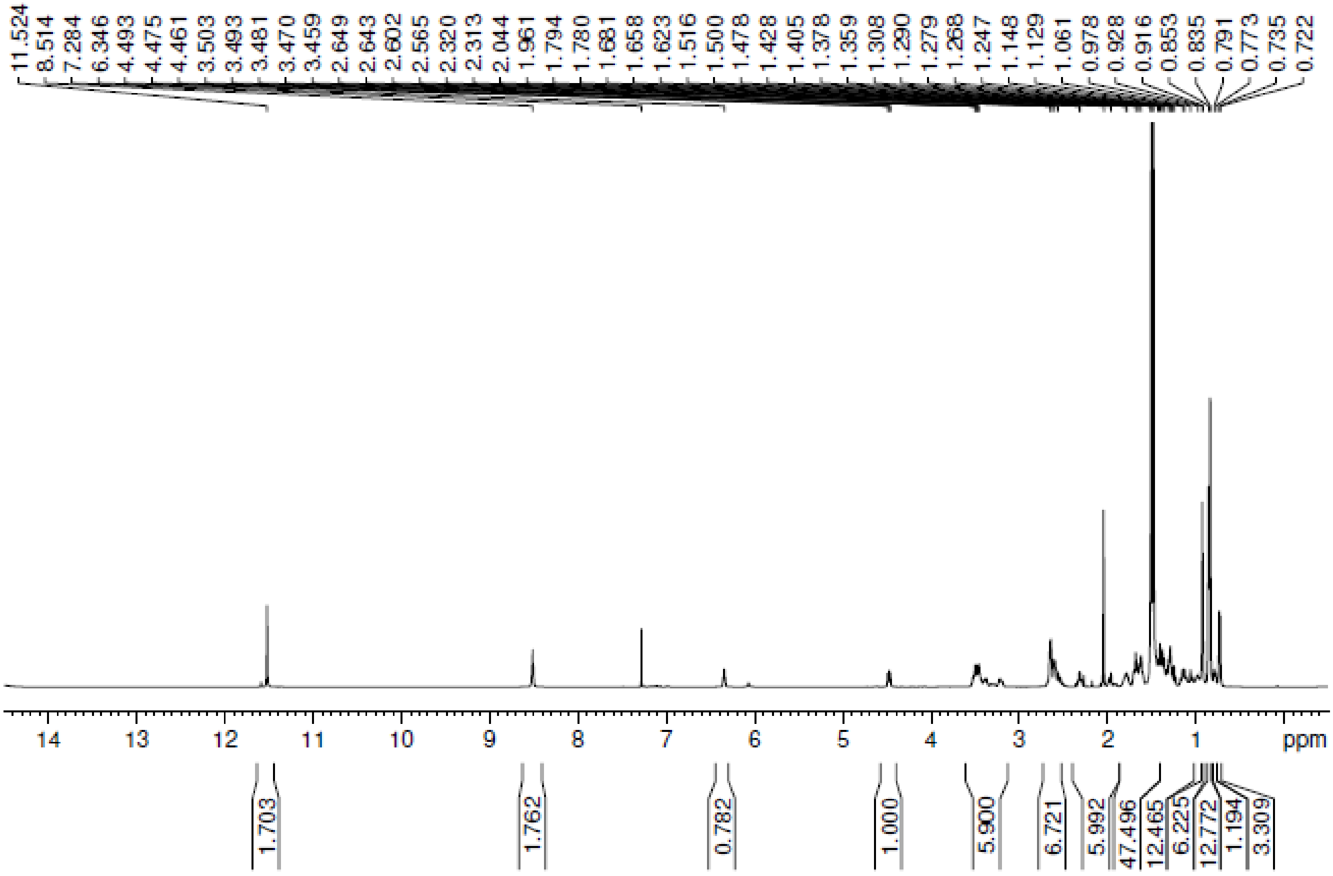

3β-N-[2-(N,N'-bis-tert-butyloxycarbonyl ethylgyanidine)-aminoethyl]-3-O-acetyl-lupane-28-amide (12). ^{13}C NMR spectra ($CDCl_3$)

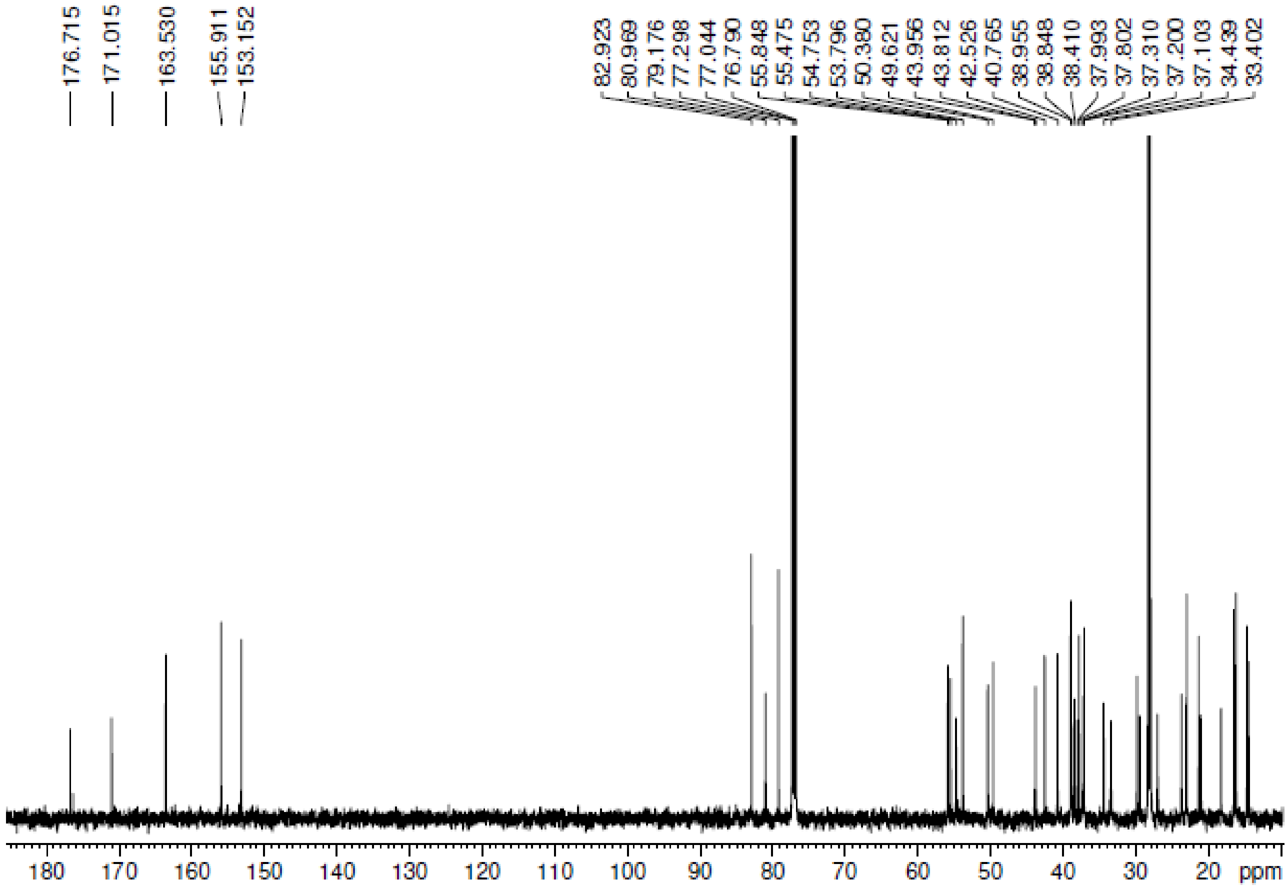

3β-N-{[3-(3-tert-butyloxycarbonyl propylgyanidine)piperazinyl]propyl}-3-O-acetyl-lupane-28-amide (13). ^{1}H NMR spectra ($CDCl_3$)

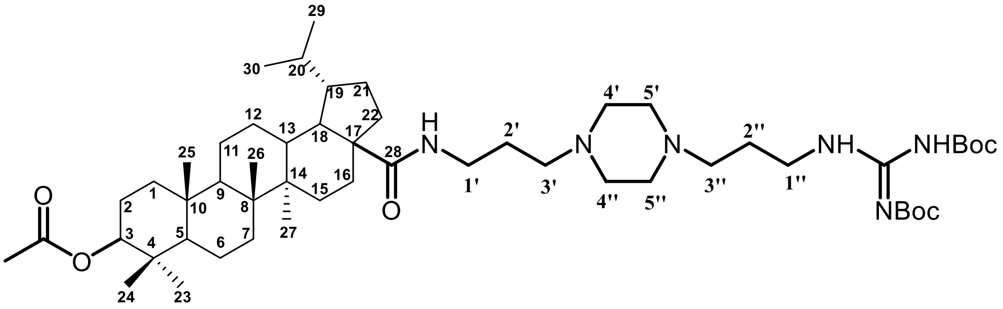

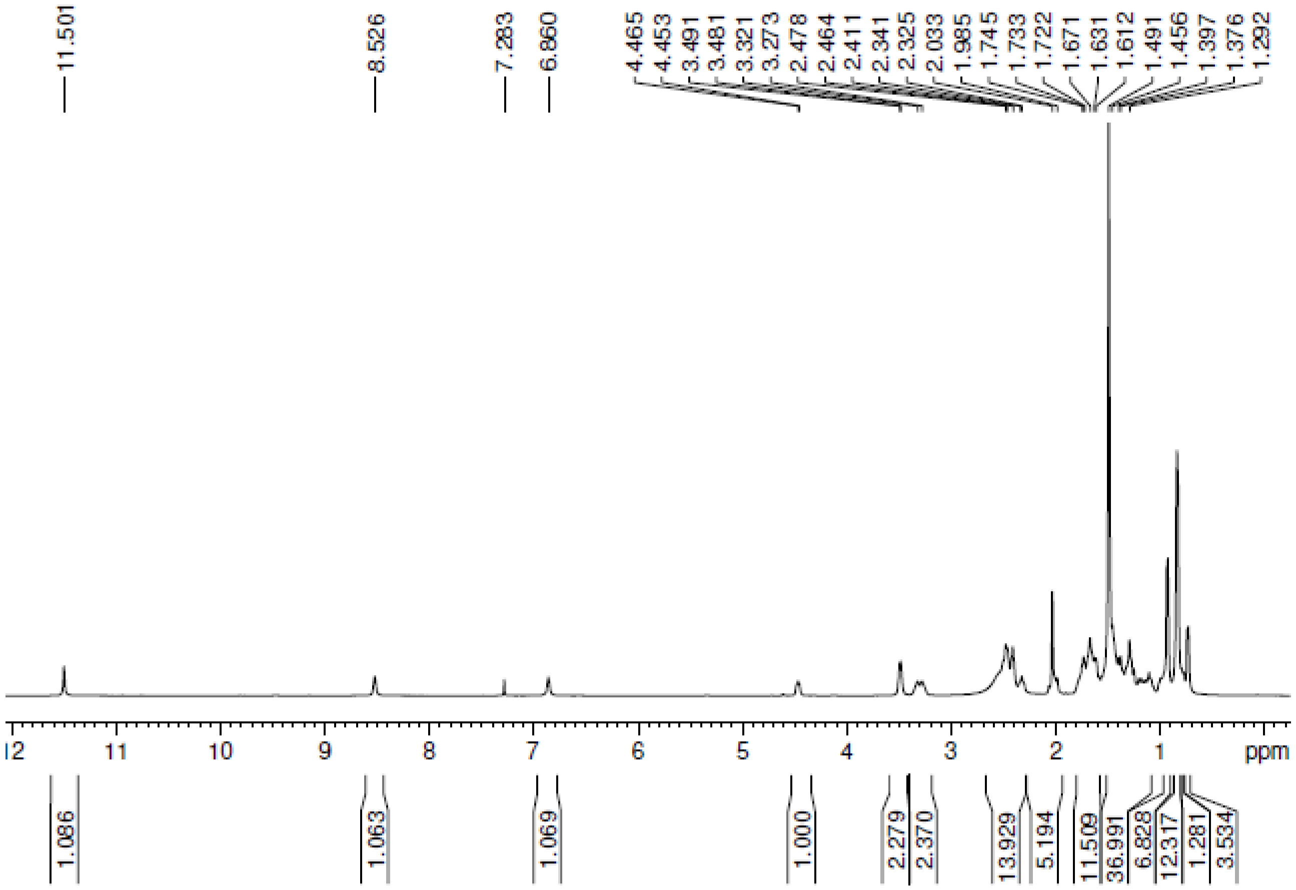

3β-N-{[3-(3- tert-butyloxycarbonyl propylgyanidine)piperazinyl]propyl}-3-O-acetyl-lupane-28-amide (13). ^{13}C NMR spectra ($CDCl_3$)

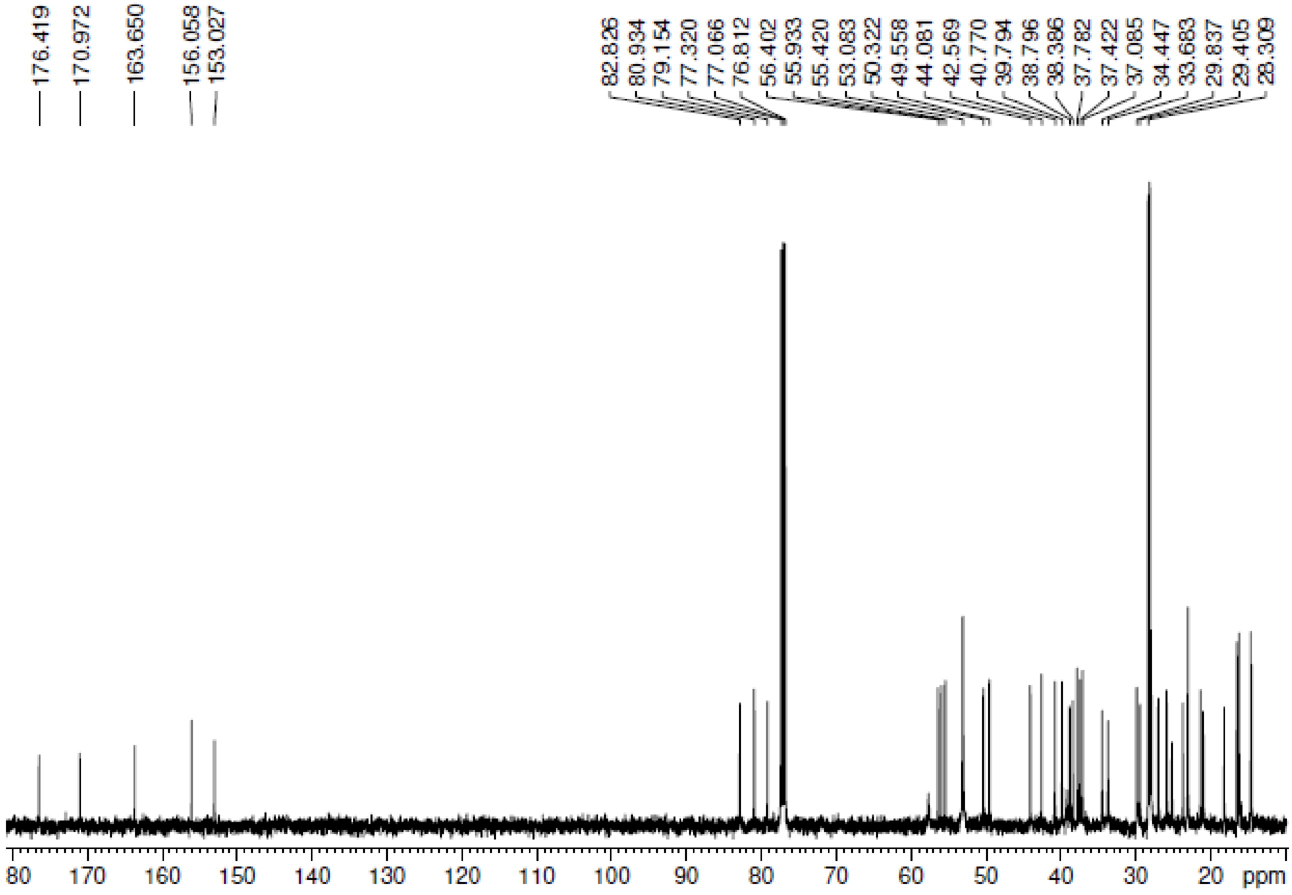

3β-[2-tert-butyloxycarbonylguanidine-3-hydroxy-2-(hydroxymethyl)propyl]-3-O-acetyl-lupane-28-oate (15a). ^{1}H NMR spectra ($CDCl_3$)

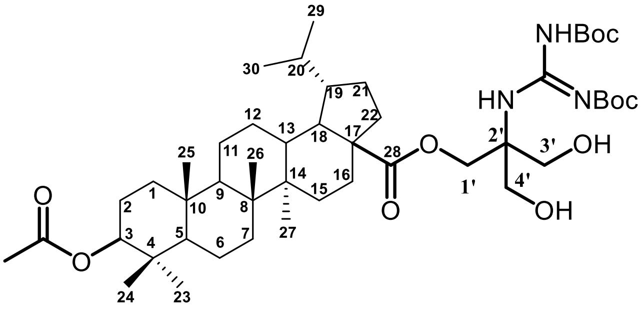

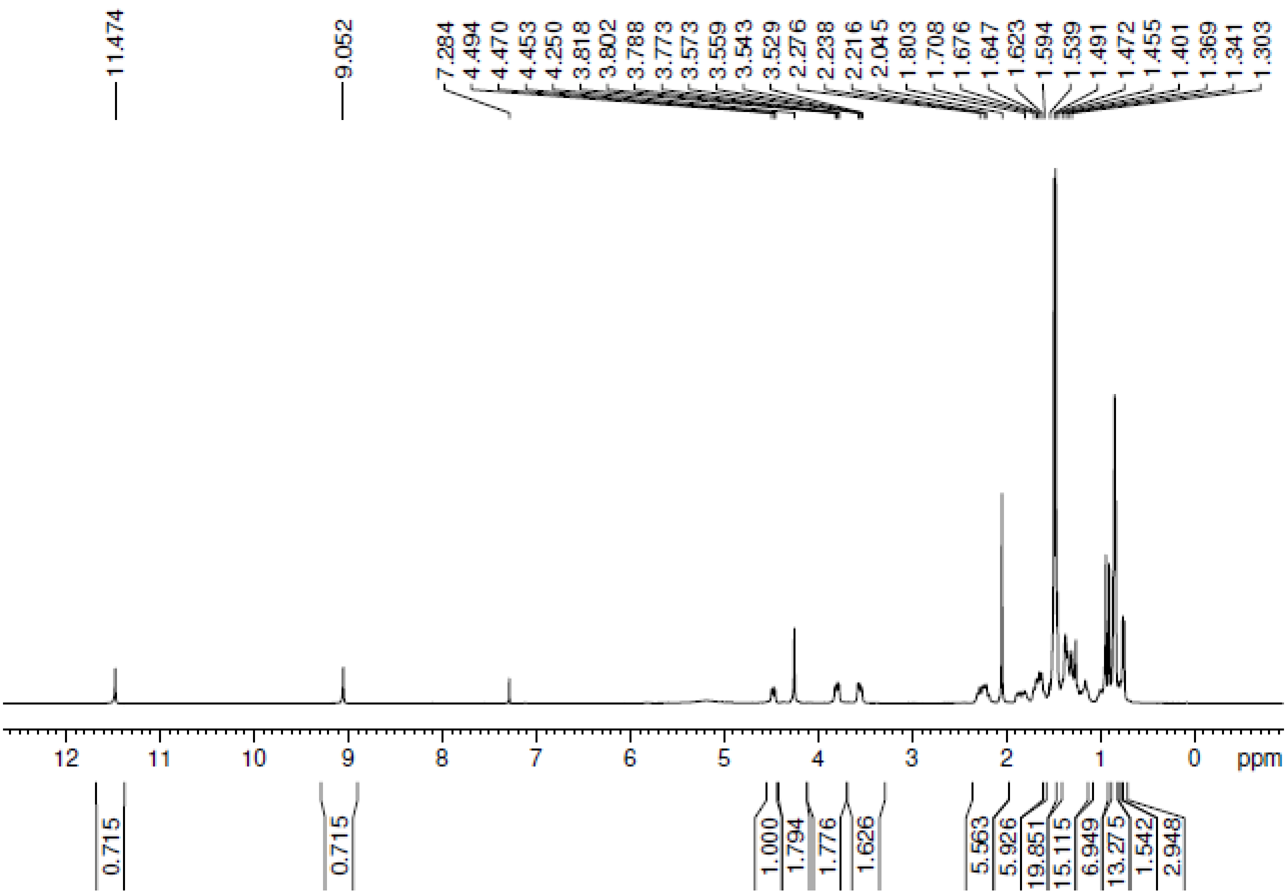

3β-[2-tert-butyloxycarbonylguanidine-3-hydroxy-2-(hydroxymethyl)propyl]-3-O-acetyl -lupane-28-oate (15a). ^{13}C NMR spectra ($CDCl_3$)

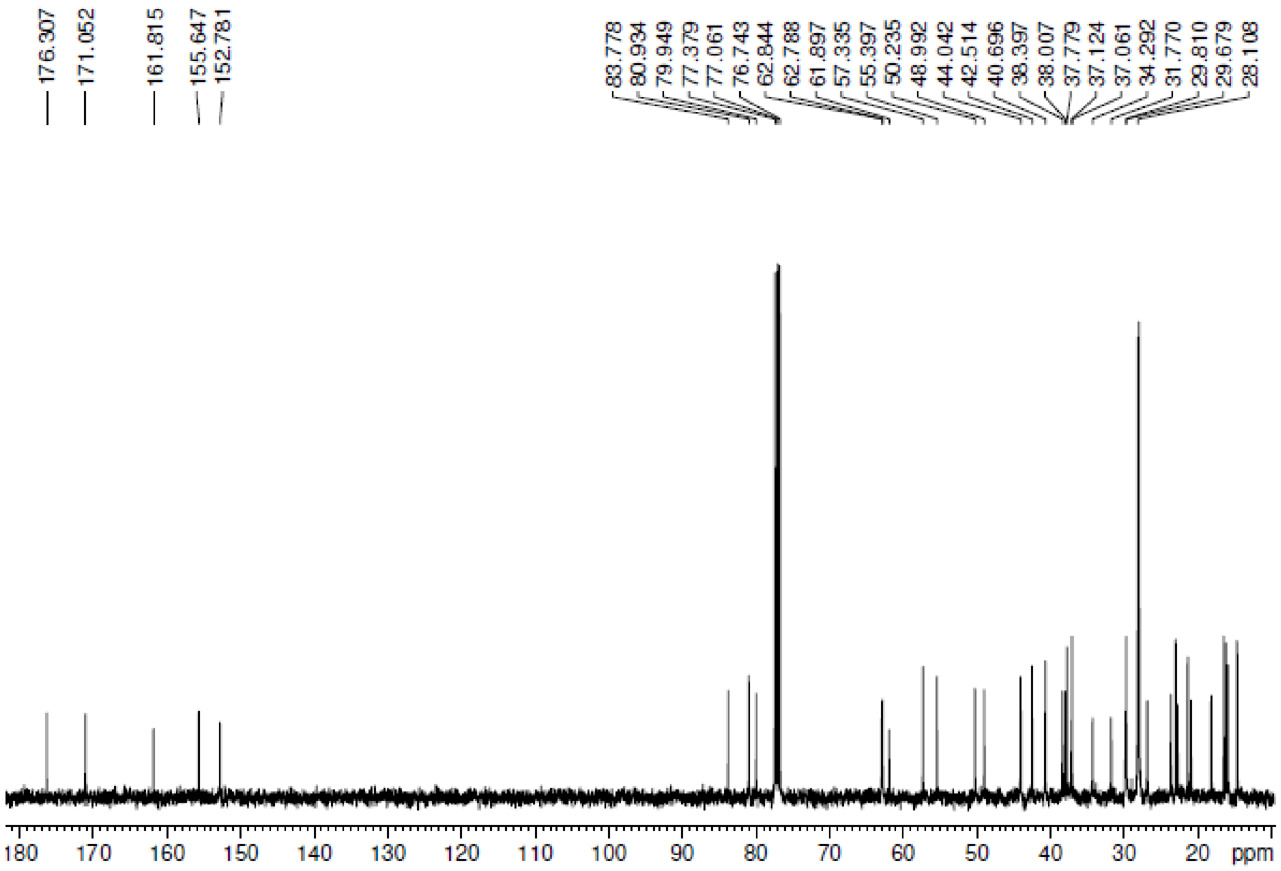

3β-[2-tert-butyloxycarbonylguanidine-3-hydroxy-2-(hydroxymethyl)propyl]-3-O-acetyl-urs-12-en-28-oate (18a). ^{1}H NMR spectra ($CDCl_3$)

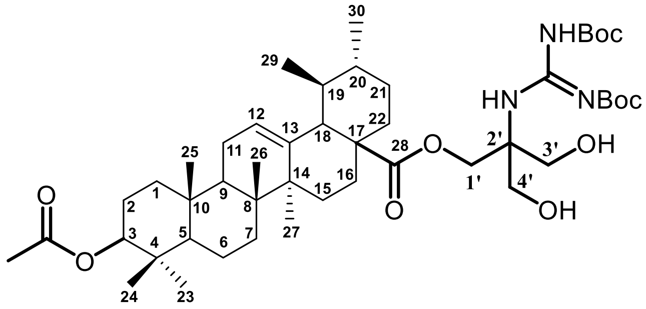

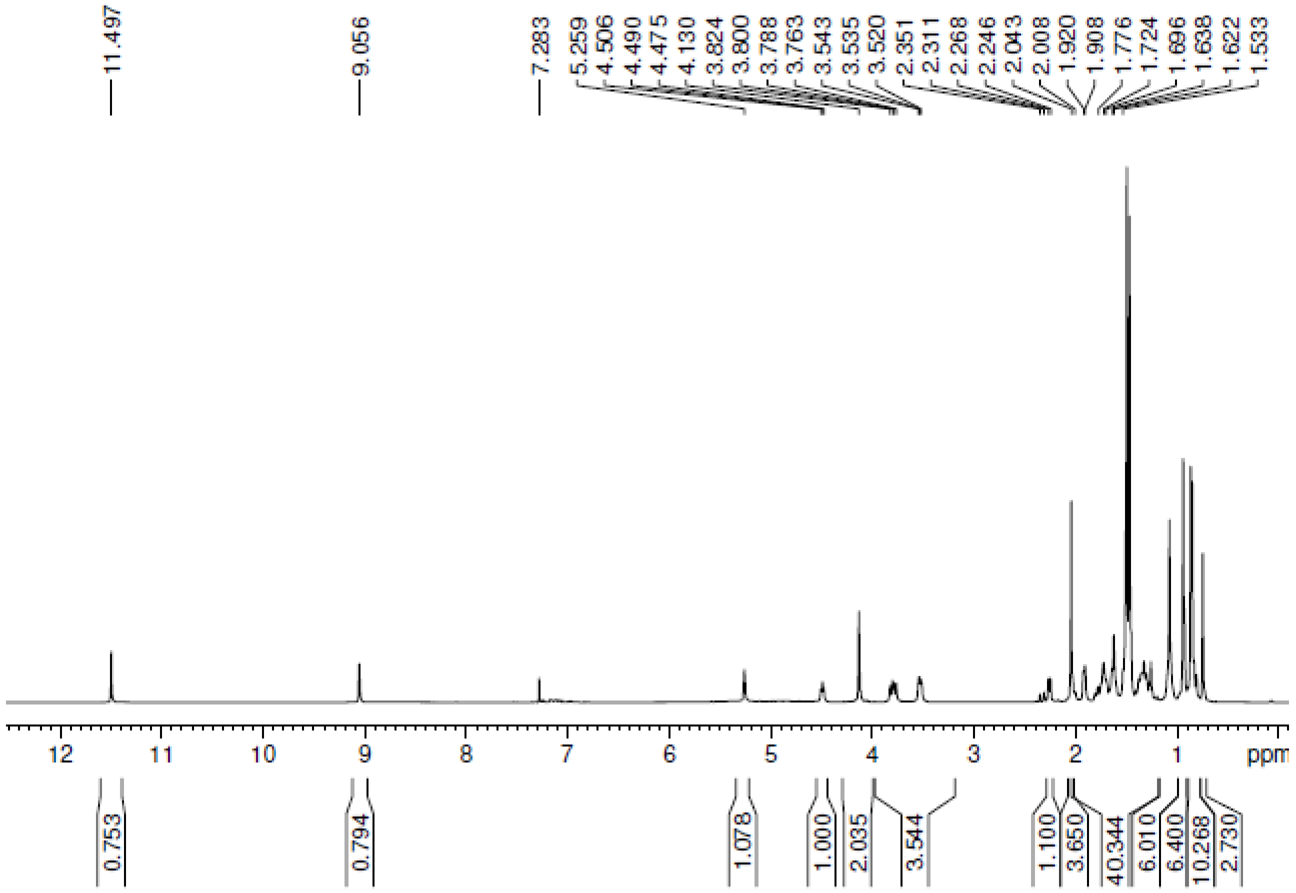

3β-[2-tert-butyloxycarbonylguanidine-3-hydroxy-2-(hydroxymethyl)propyl]-3-O-acetyl-urs-12-en-28-oate (18a). ^{13}C NMR spectra ($CDCl_3$)

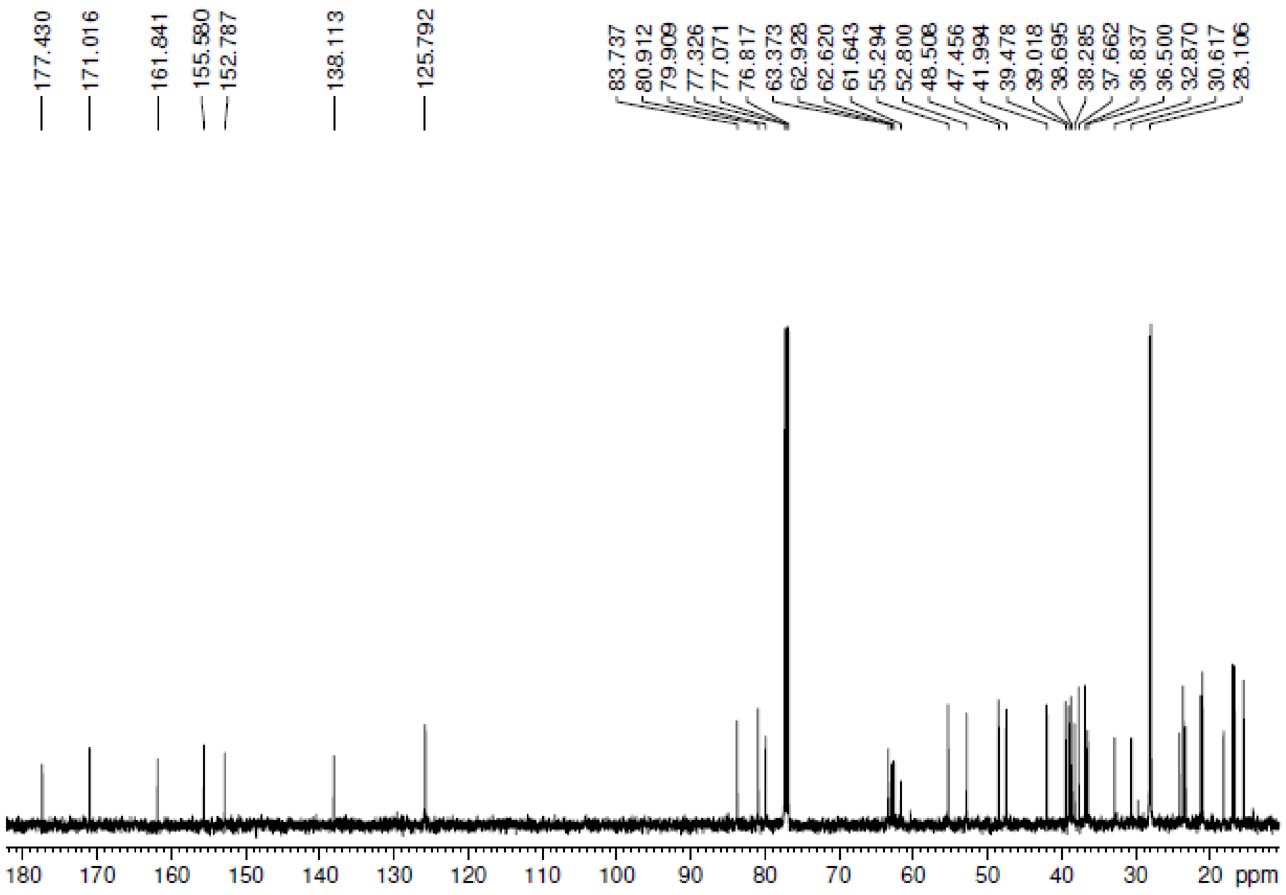

3β-[2-tert-butyloxycarbonylguanidine-3-hydroxy-2-(hydroxymethyl)propyl]-3-O-acetyl-olean-12-en-28-oate (20a). ^{1}H NMR spectra ($CDCl_3$)

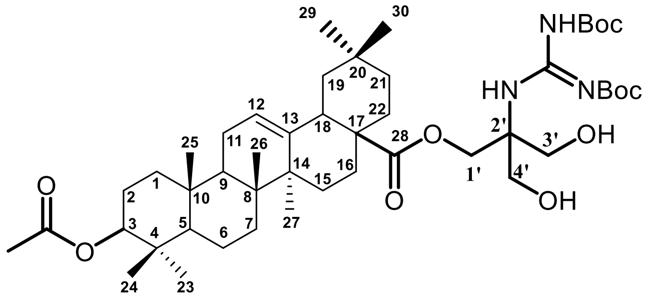

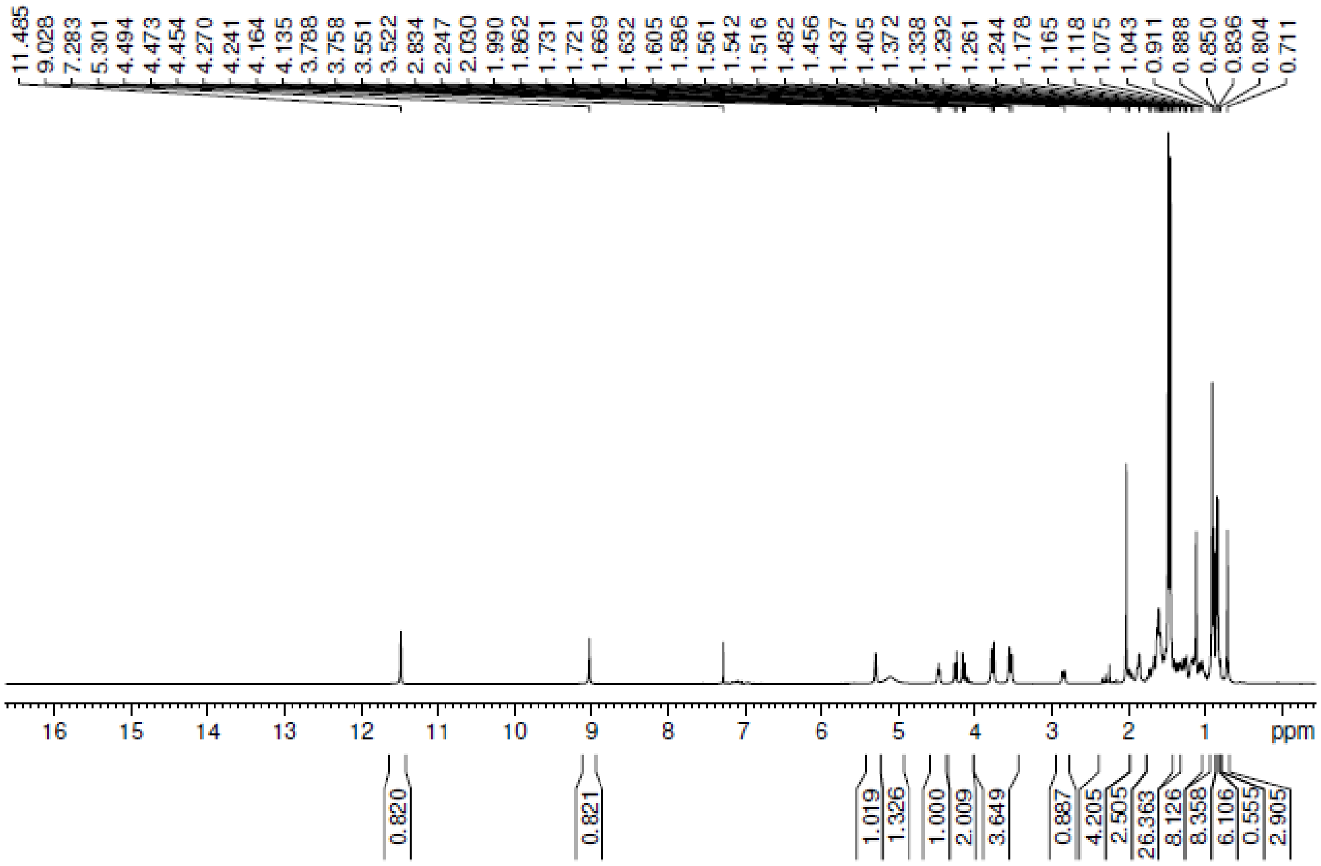

3β-[2-tert-butyloxycarbonylguanidine-3-hydroxy-2-(hydroxymethyl)propyl]-3-O-acetyl - olean-12-en-28-oate (20a). ^{13}C NMR spectra ($CDCl_3$)

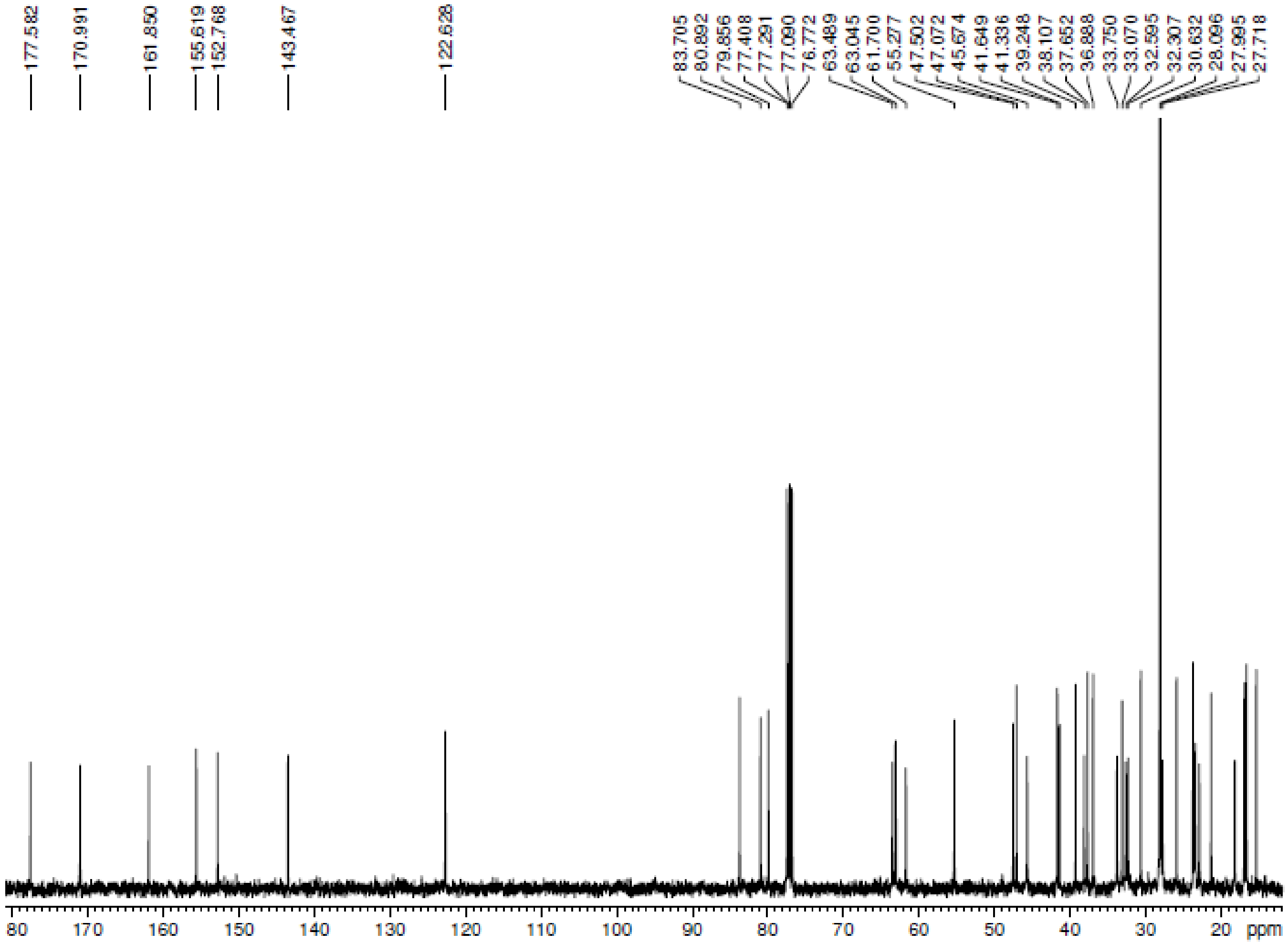

N-(4-buthylgyanidine)-3-oxolupane-28-amide trifluoroacetate (9a). ^{1}H NMR spectra ($CDCl_3$)

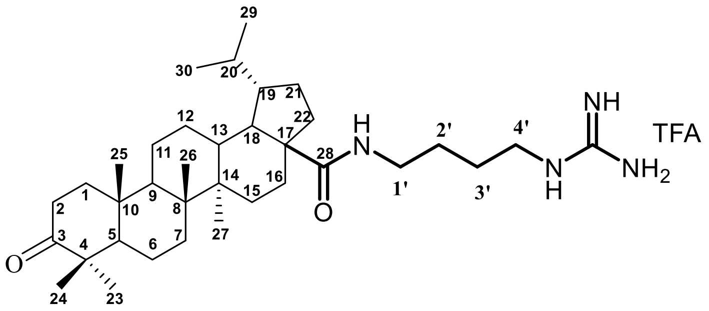

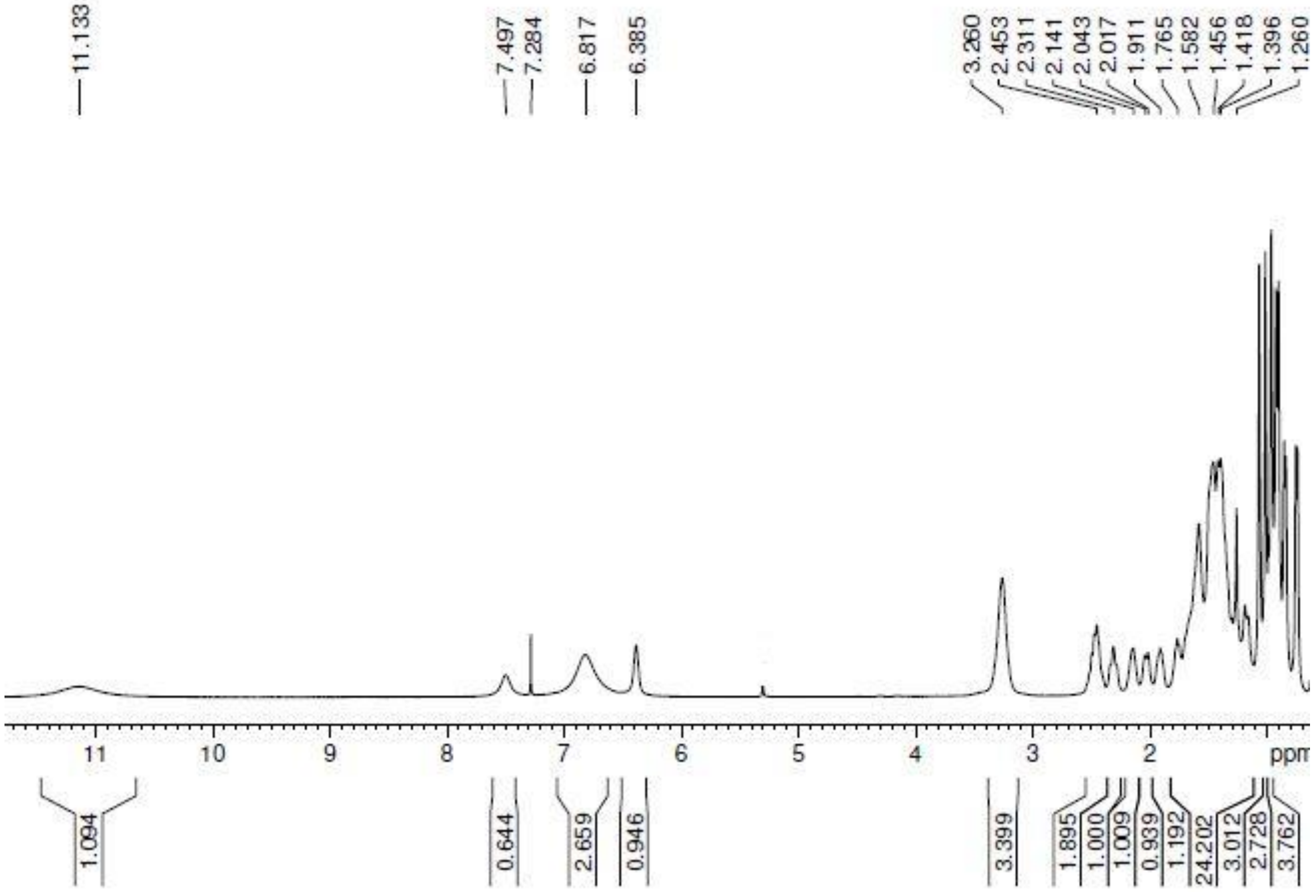

N-(4-buthylgyanidine)-3-oxolupane-28-amide trifluoroacetate (9a). ^{13}C NMR spectra ($CDCl_3$)

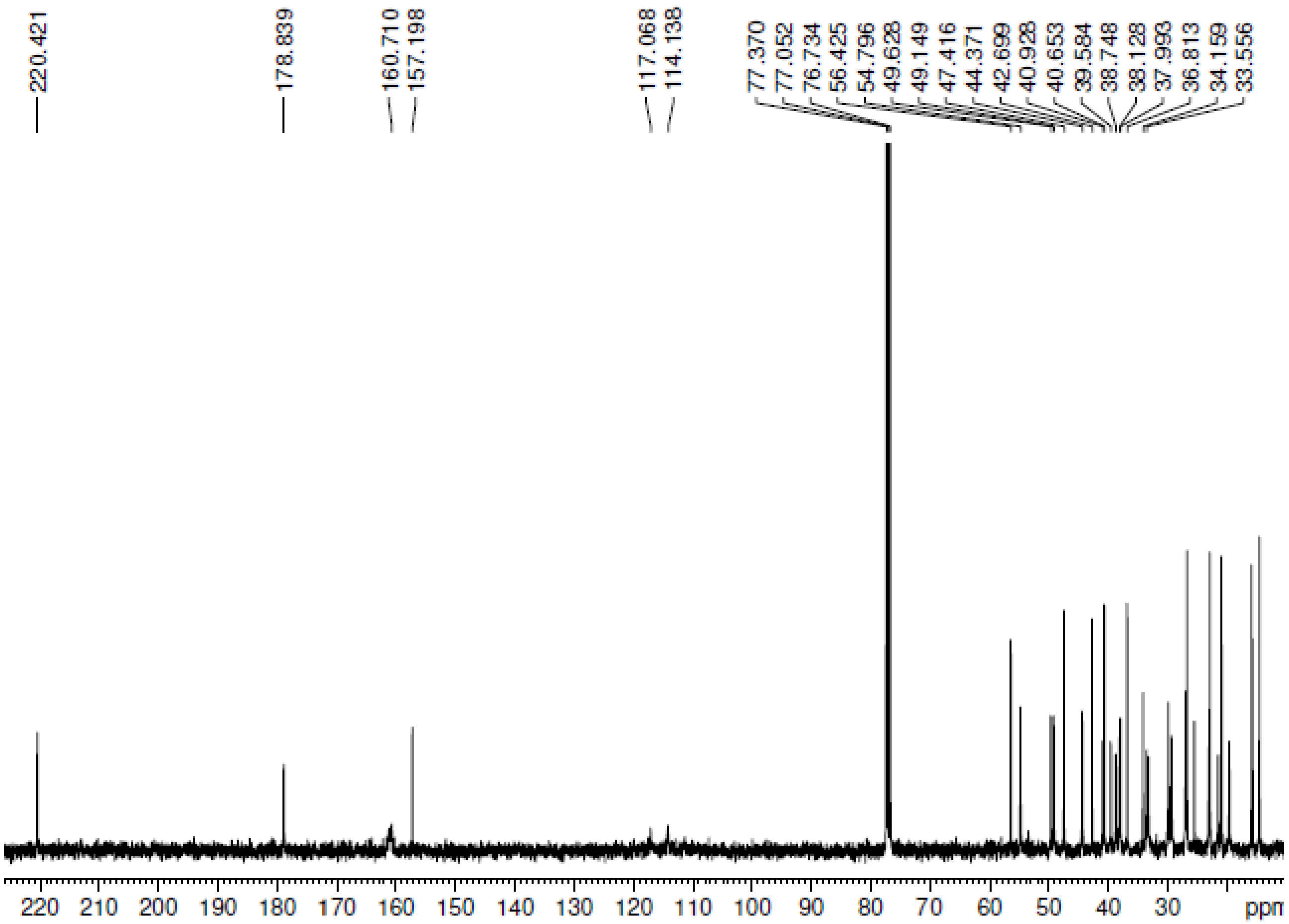

N-(4-buthylgyanidine)-3-oxolupane-28-amide trifluoroacetate (9a). ^{19}F NMR spectra ($CDCl_3$)

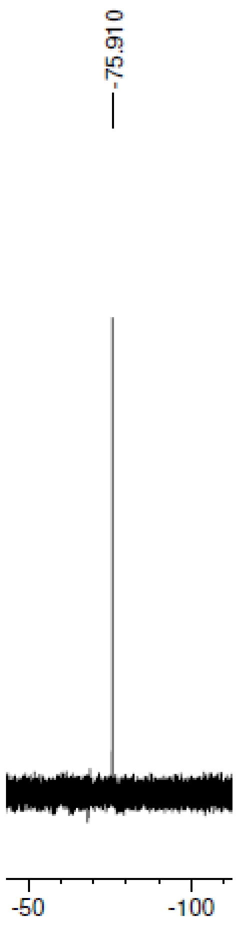

3β-N-(2-ethylgyanidine)-3-O-acetyl-lupane-28-amide trifluoroacetate (10a). ^{1}H NMR spectra (CDCl$_3$)

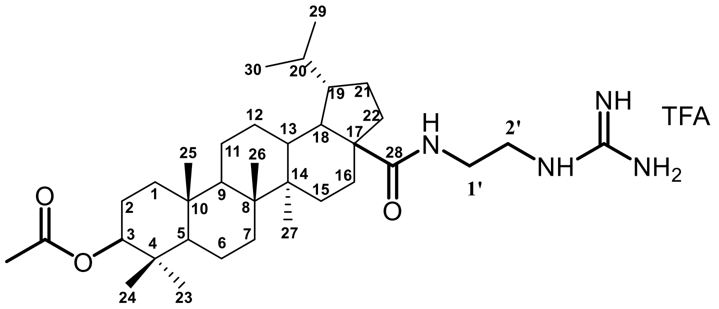

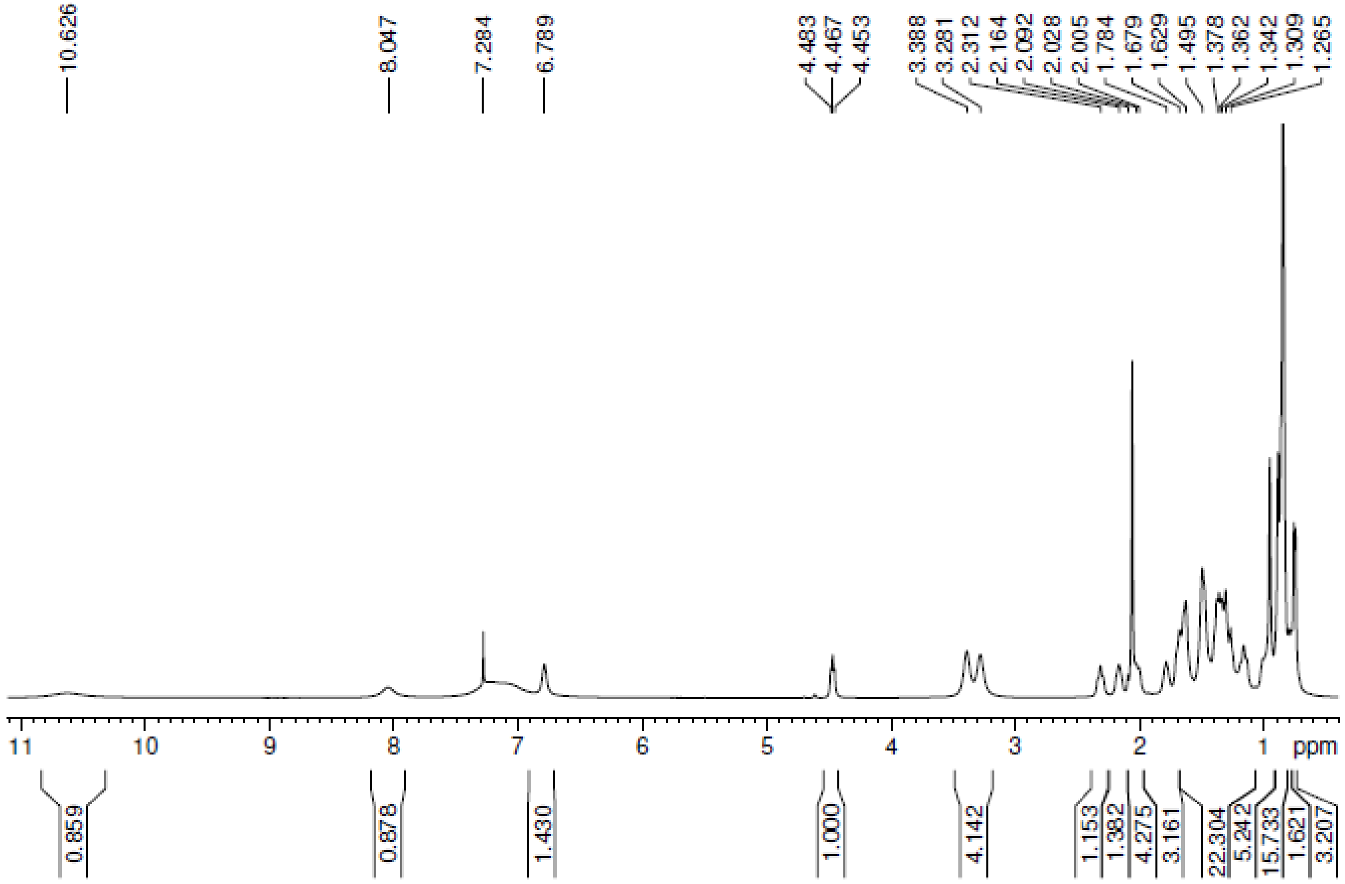

3β-N-(2-ethylgyanidine)-3- O-acetyl-lupane-28-amide trifluoroacetate (10a). ^{13}C NMR spectra ($CDCl_3$)

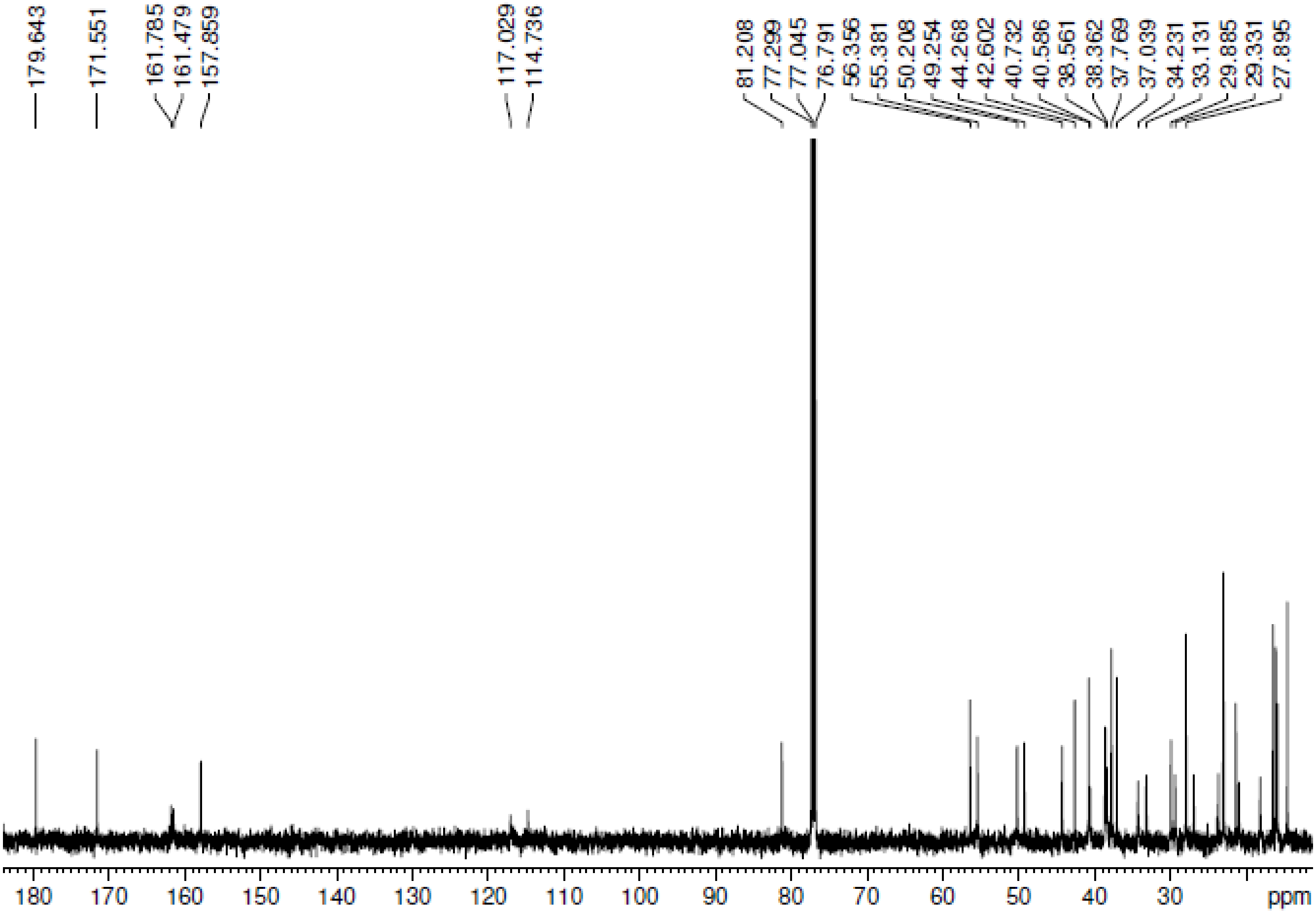

3β-N-(2-ethylgyanidine)-3- O-acetyl-lupane-28-amide trifluoroacetate (10a). ^{13}F NMR spectra (CDCl$_3$)

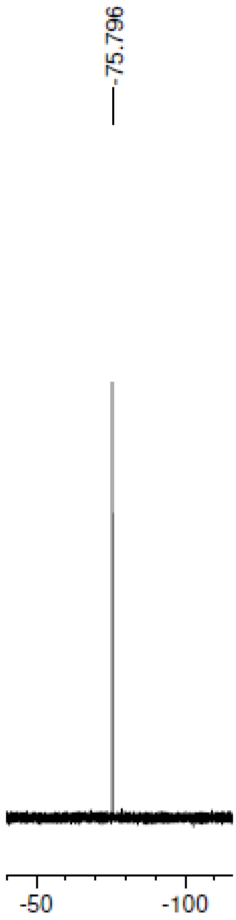

3β-N-(4-buthylgyanidine)-3-O-acetyl-lupane-28-amide trifluoroacetate (11a). ^{1}H NMR spectra (MeOD)

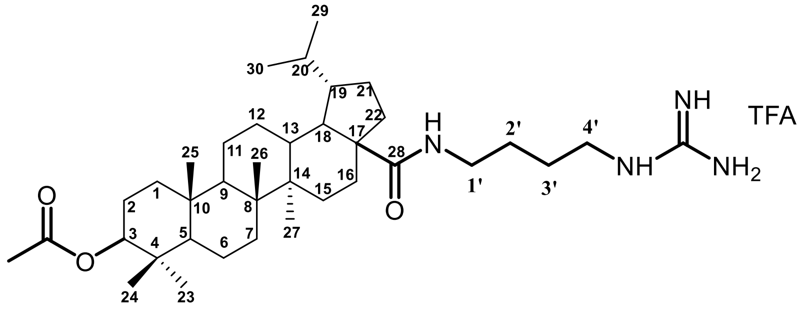

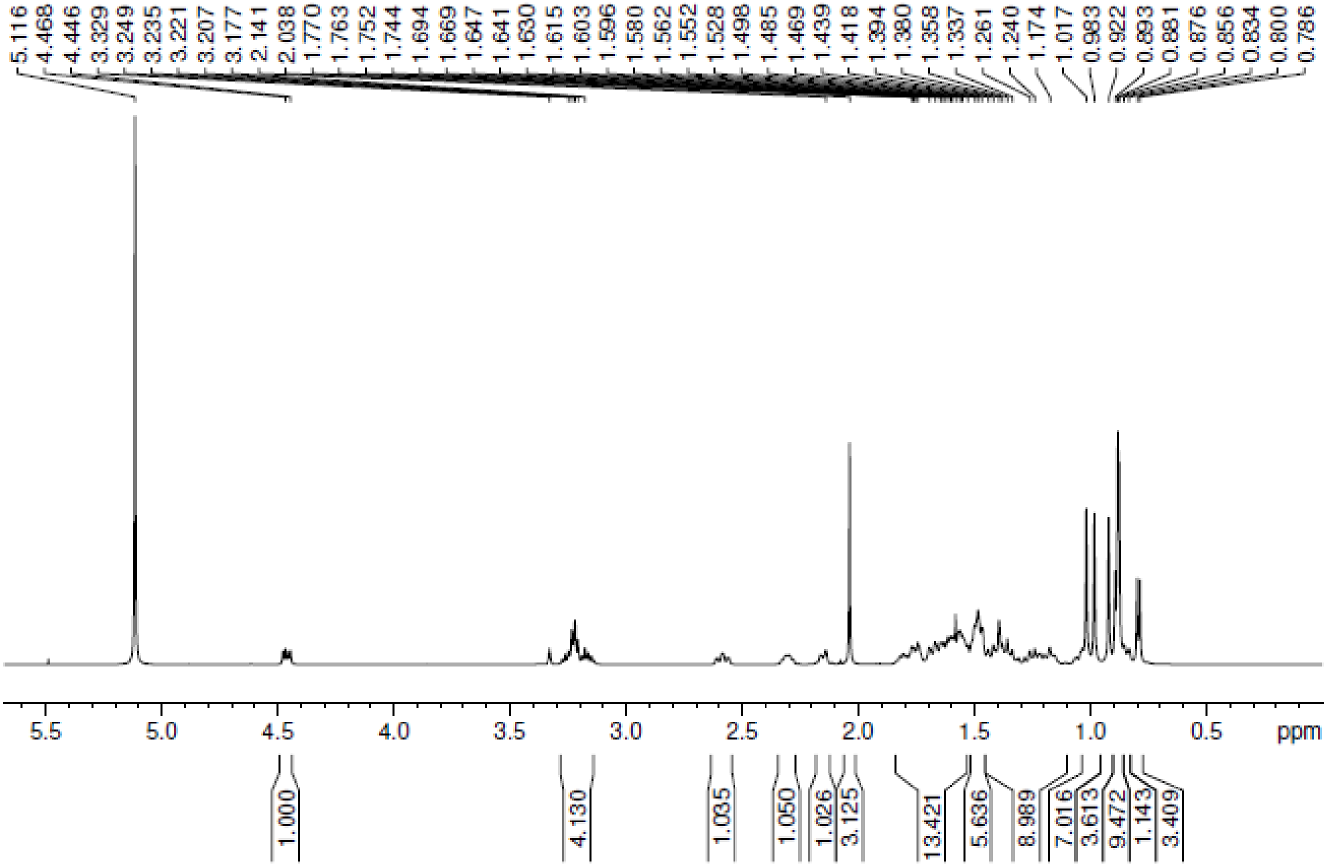

3β-N-(4-buthylgyanidine)-3- O-acetyl-lupane-28-amide trifluoroacetate (11a). ^{13}C NMR spectra (MeOD)

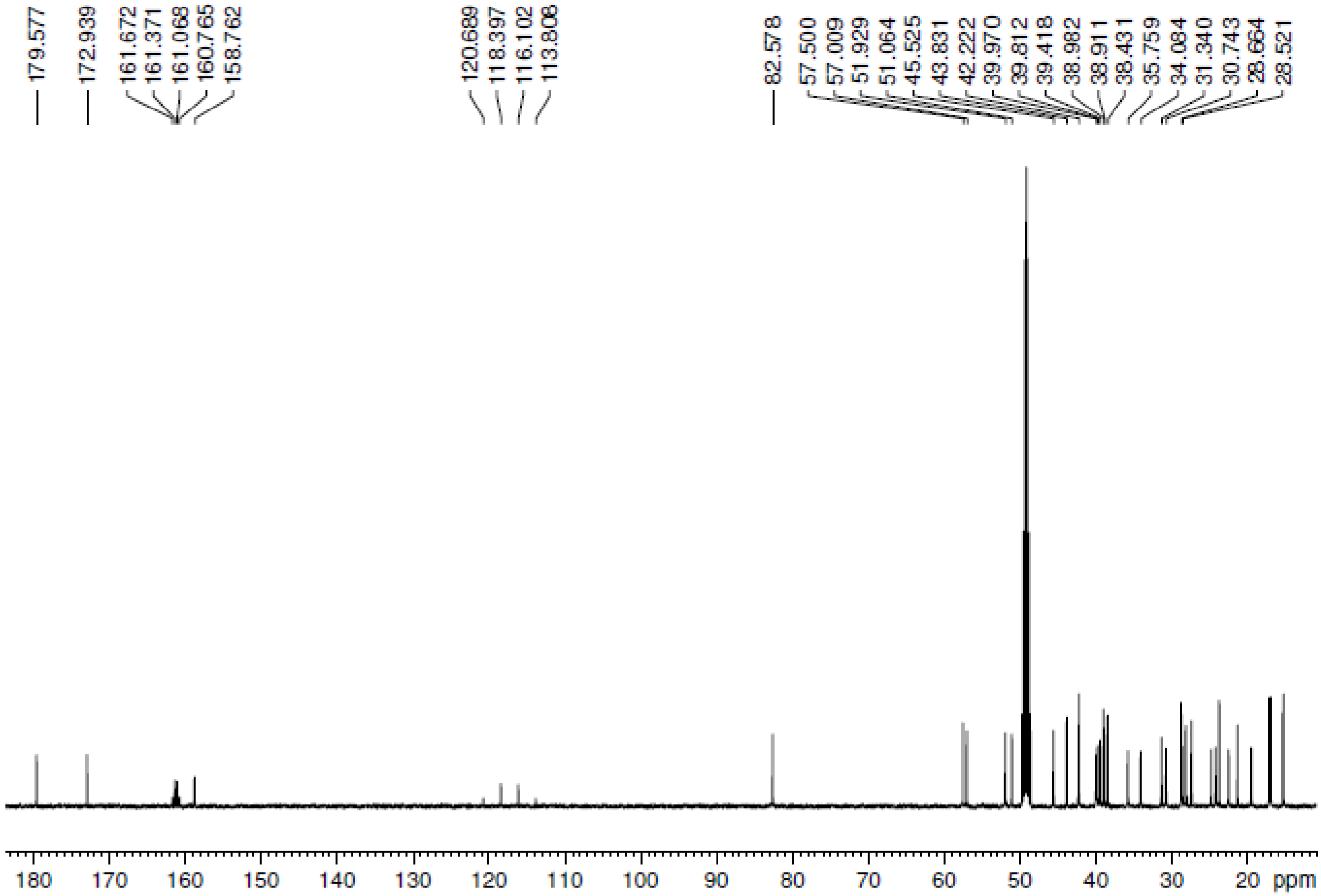

3β-N-(4-buthylgyanidine)-3- O-acetyl-lupane-28-amide trifluoroacetate (11a). ^{19}F NMR spectra (MeOD)

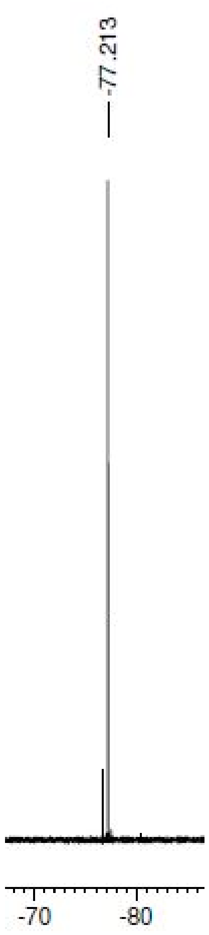

3β-N-[2-(N,N’-bis-ethylgyanidine)-ethyl]-3-O-acetyl-lupane-28-amide trifluoroacetate (12a)

^{1}H NMR spectra (MeOD)

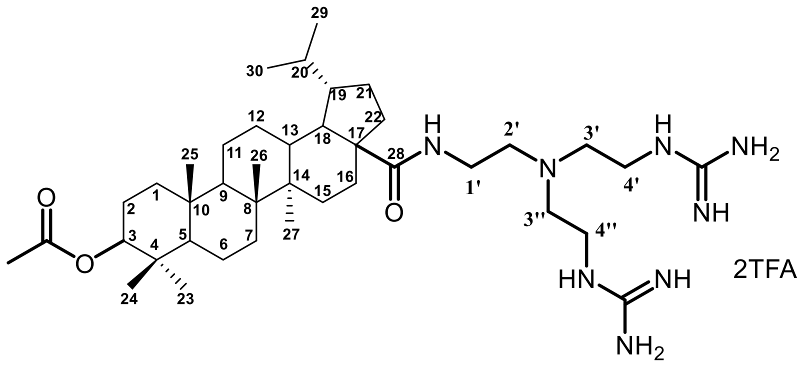

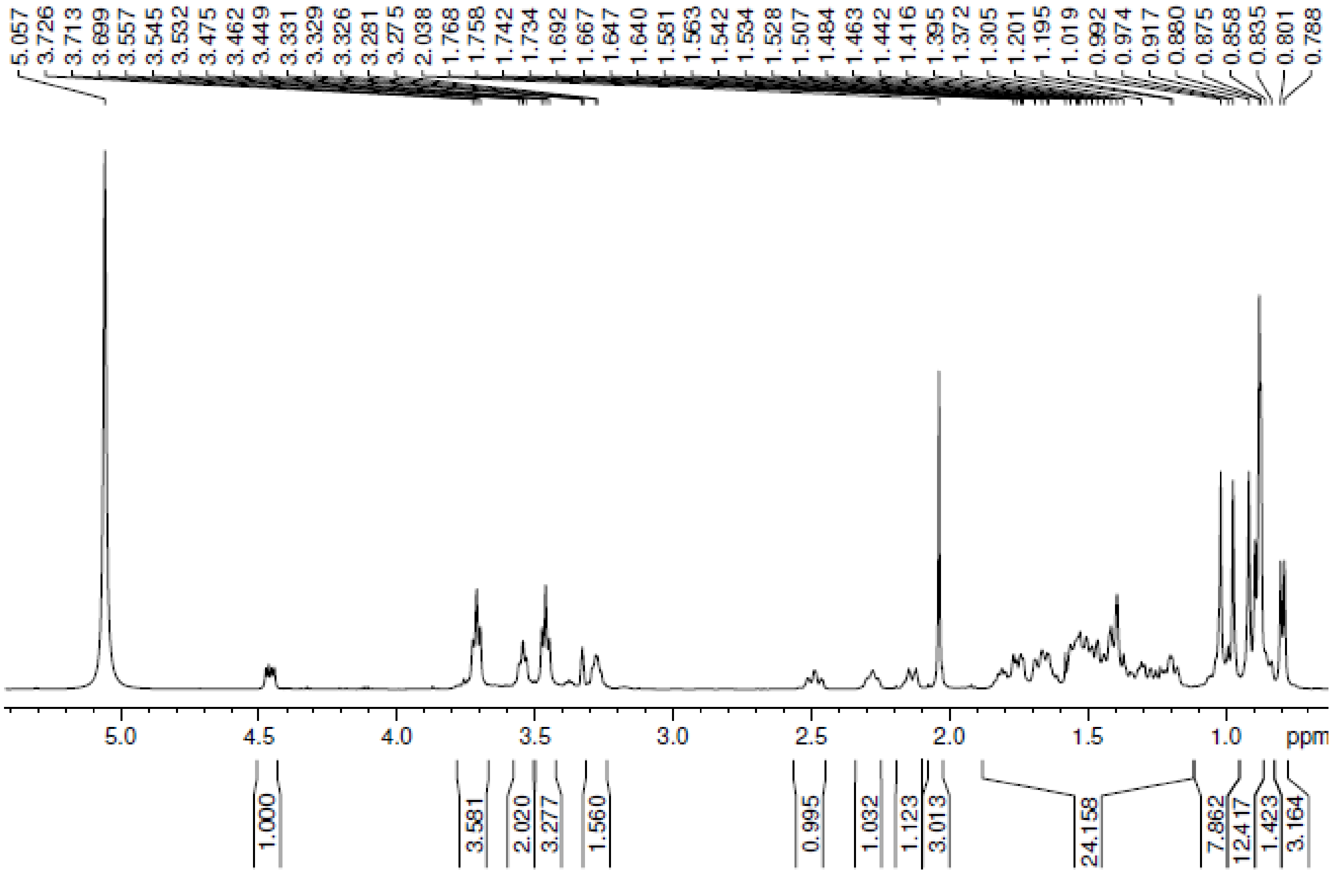

3β-N-[2-(N,N'-bis-ethylgyanidine)-ethyl]-3- O-acetyl -lupane-28-amide trifluoroacetate (12a).

^{13}C NMR spectra (MeOD)

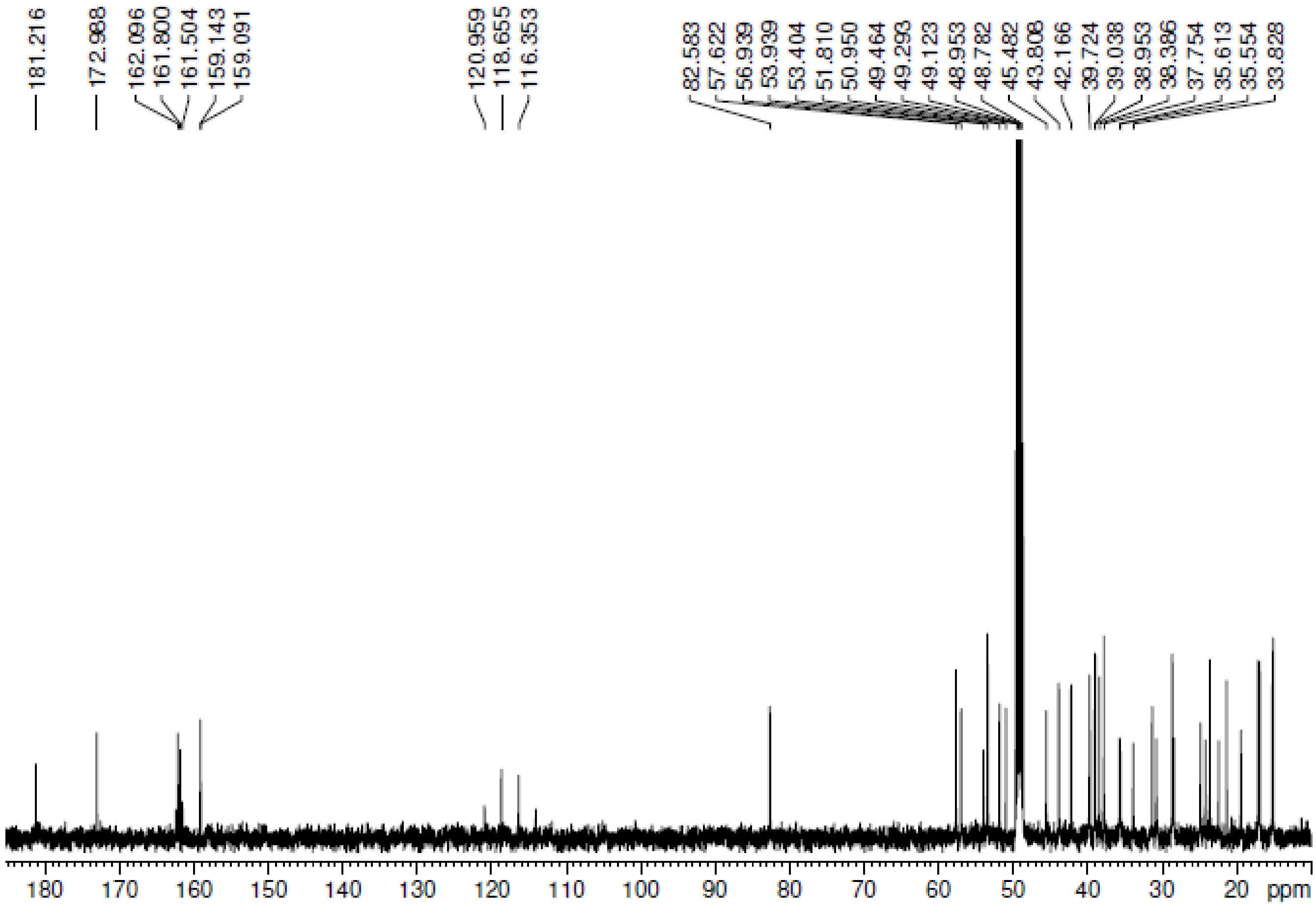

3β-N-[2-(N,N'-bis-ethylgyanidine)-ethyl]-3- O-acetyl -lupane-28-amide trifluoroacetate (12a).

^{19}F NMR spectra (MeOD)

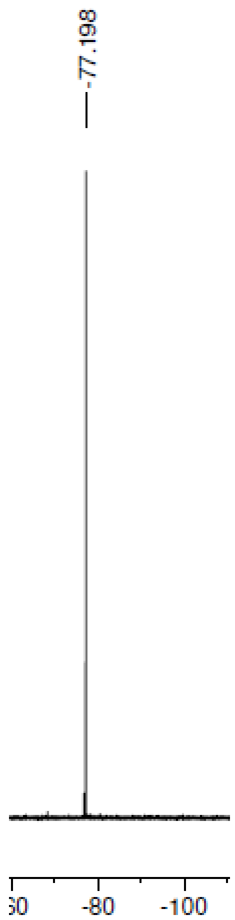

3β-N-{[3-(3-propylguanidine)piperazinyl]propyl}-3-O-acetyl-lupane-28-amide trifluoroacetate (13a).

^{1}H NMR spectra (MeOD)

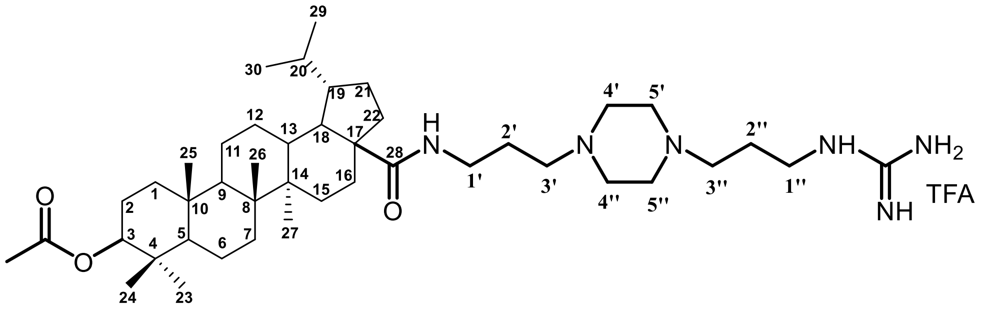

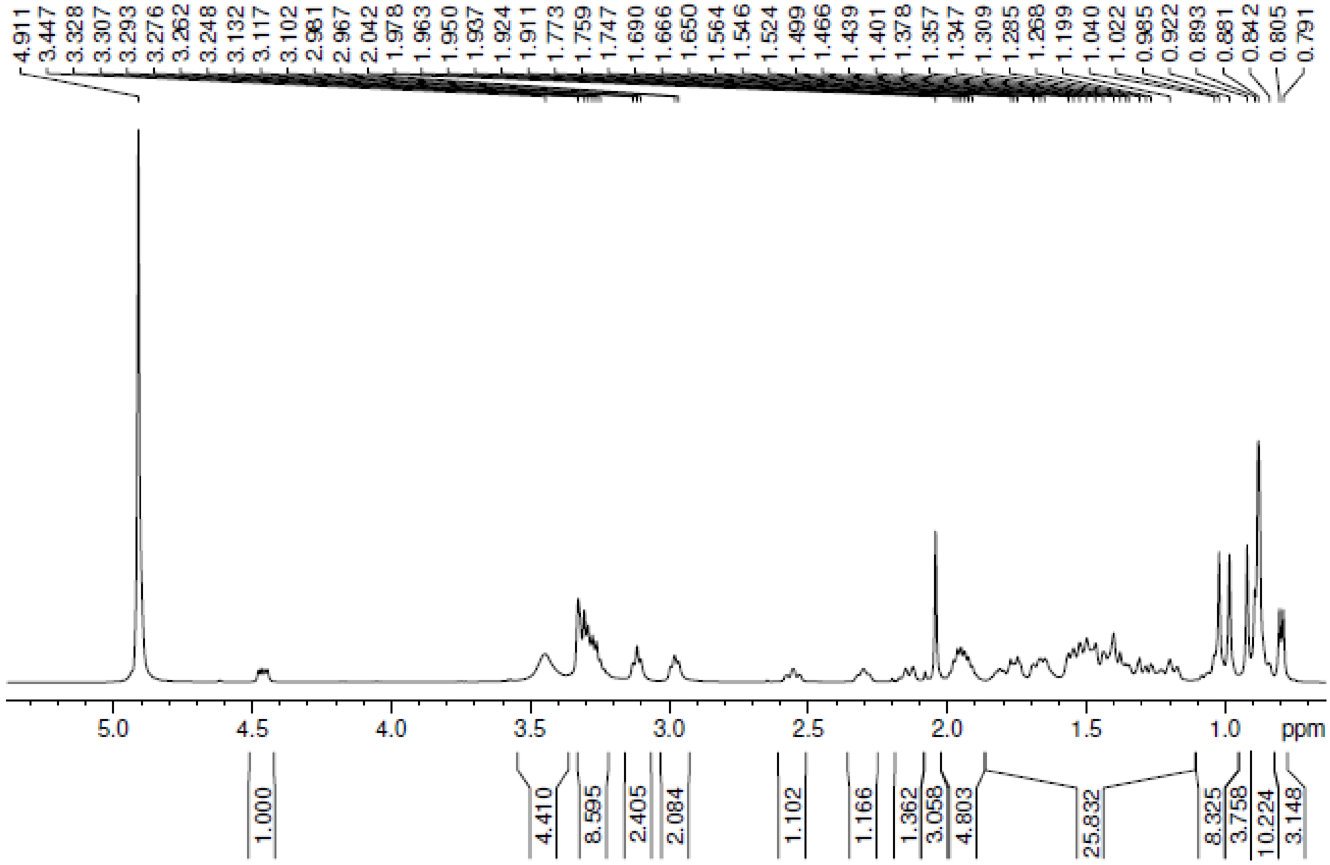

3β-N-{[3-(3-propylguanidine)piperazinyl]propyl}-3-O-acetyl-lupane-28-amide trifluoroacetate (13a).

^{13}C NMR spectra (MeOD)

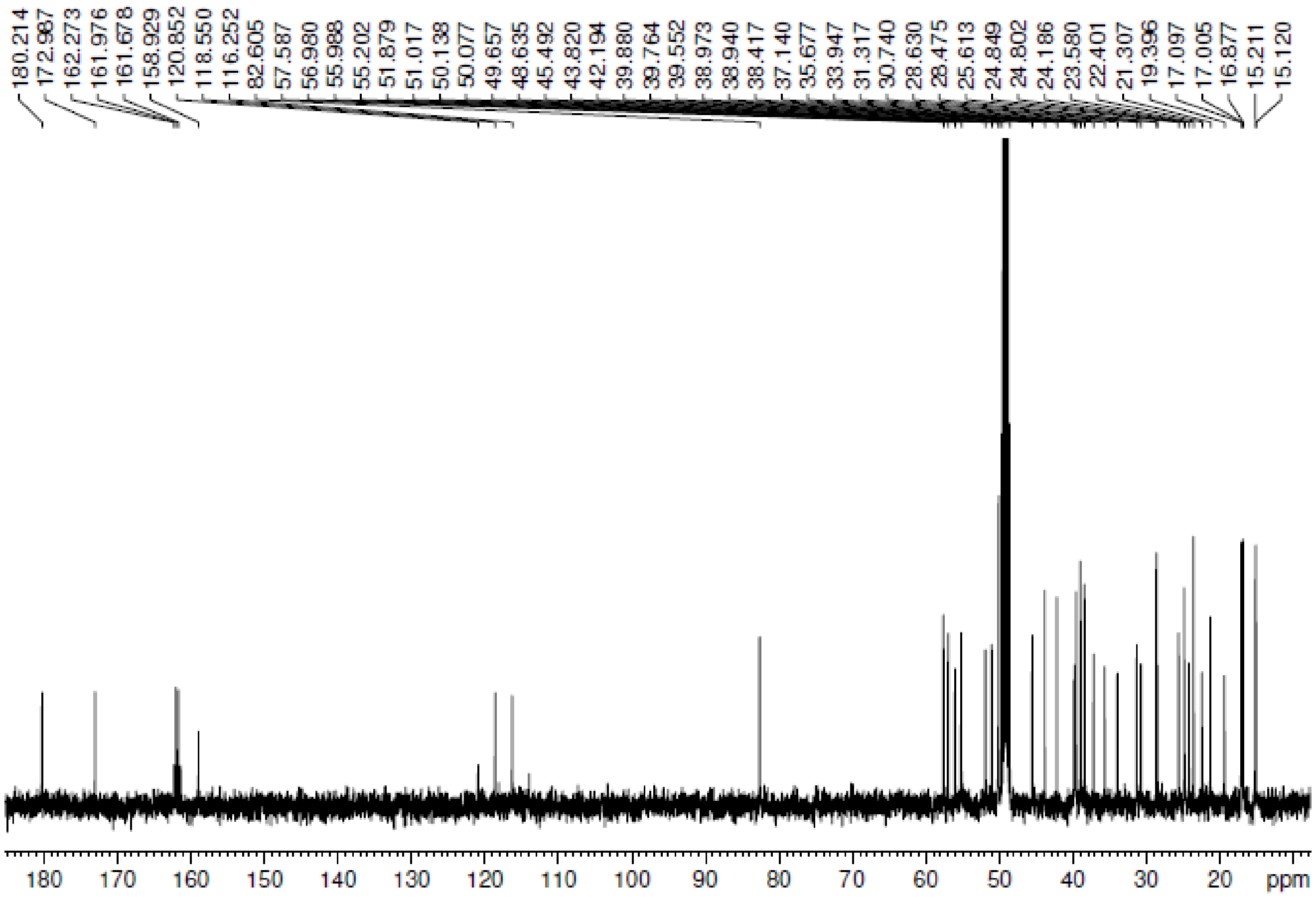

3β-N-{[3-(3-propylguanidine)piperazinyl]propyl}-3-O-acetyl-lupane-28-amide trifluoroacetate (13a).

^{19}F NMR spectra (MeOD)

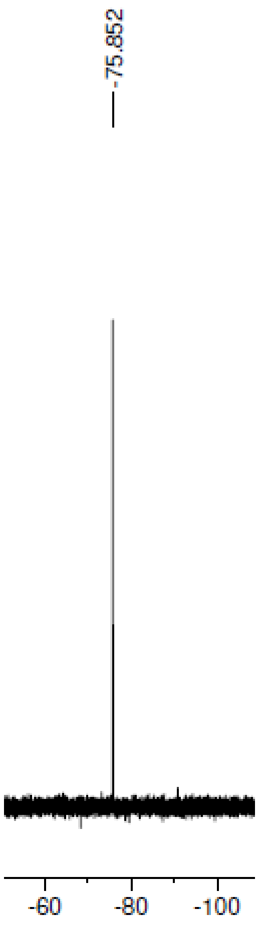

3β-[2-guanidine-3-hydroxy-2-(hydroxymethyl)propyl]-3-O-acetyl-lupane-28-oate trifluoroacetate (15b). ^{1}H NMR spectra ($CDCl_3$)

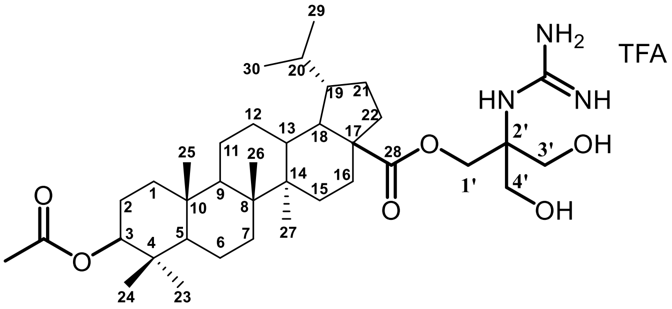

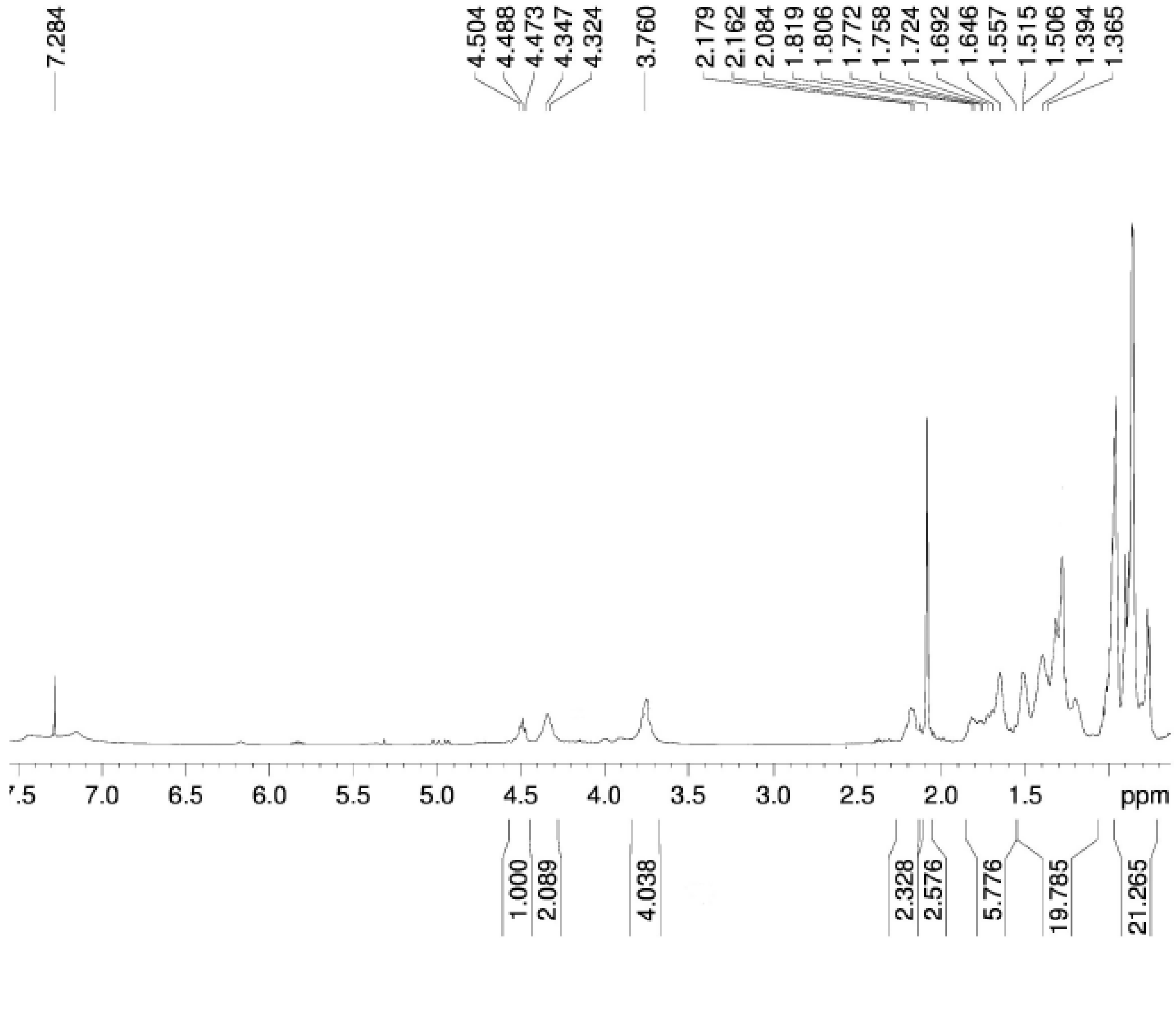

3β-[2-guanidine-3-hydroxy-2-(hydroxymethyl)propyl]-3-O-acetyl-lupane-28-oate trifluoroacetate (15b).^{13}C NMR spectra ($CDCl_3$)

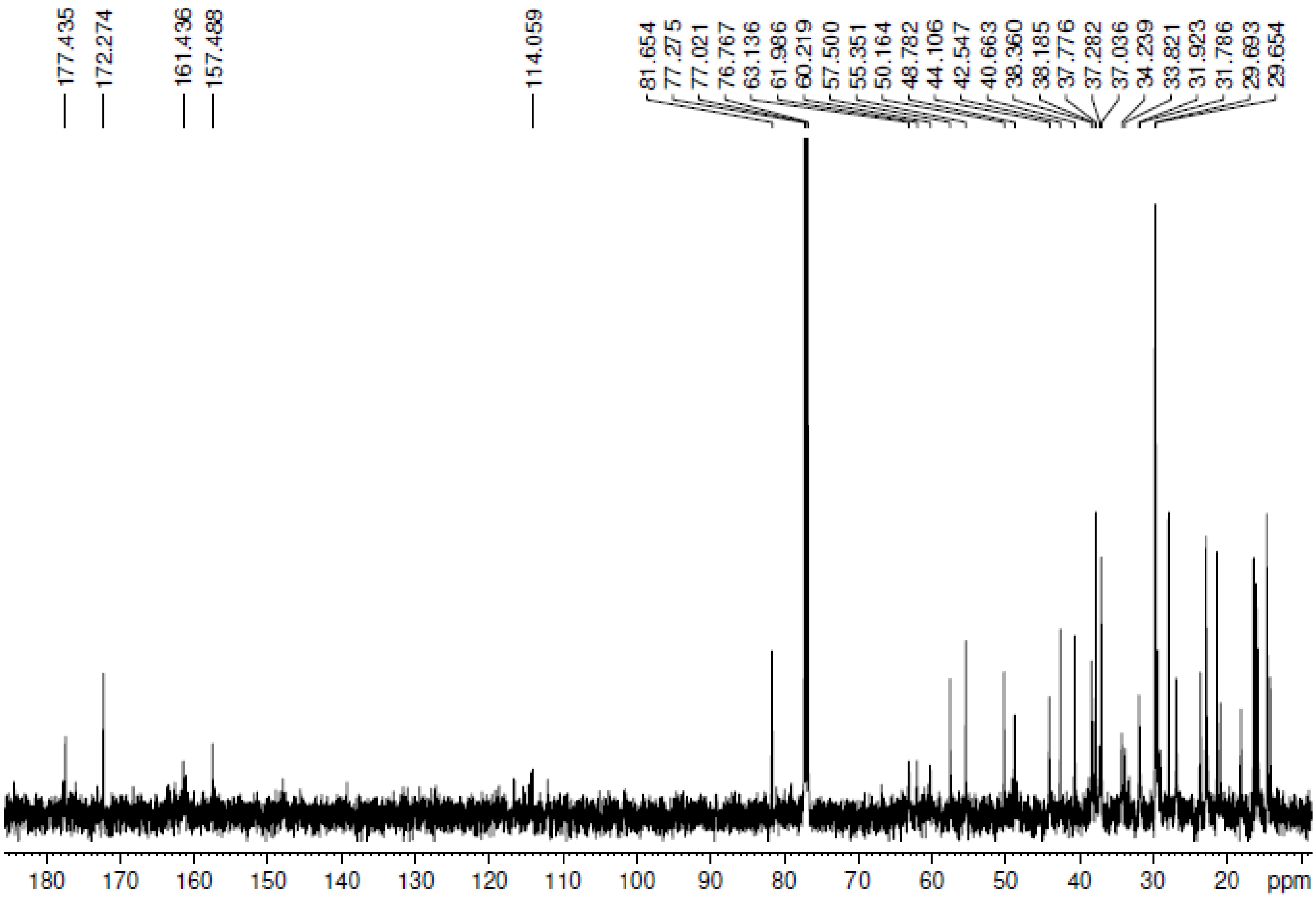

3β-[2-guanidine-3-hydroxy-2-(hydroxymethyl)propyl]-3-O-acetyl-lupane-28-oate trifluoroacetate (15b). ^{19}F NMR spectra ($CDCl_3$)

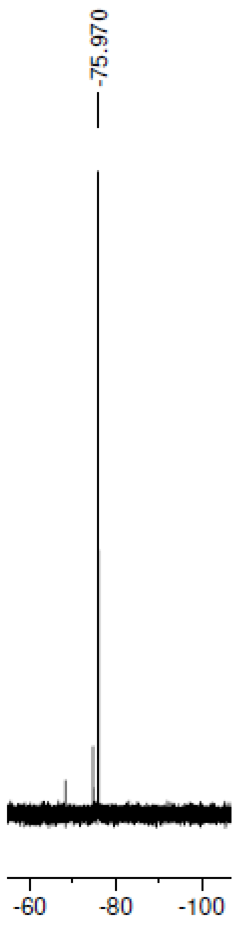

3β-[2-guanidine-3-hydroxy-2-(hydroxymethyl)propyl]-3-O-acetyl-urs-12-en-28-oate trifluoroacetate (18b). ^{1}H NMR spectra (MeOD)

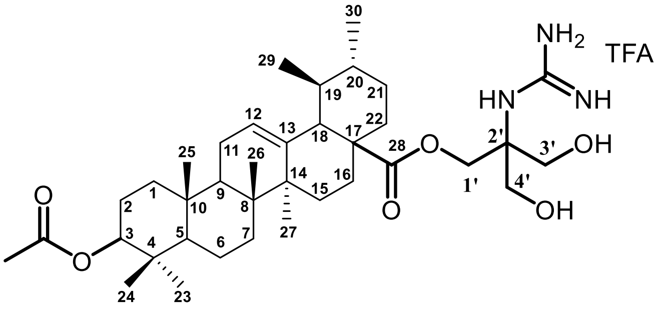

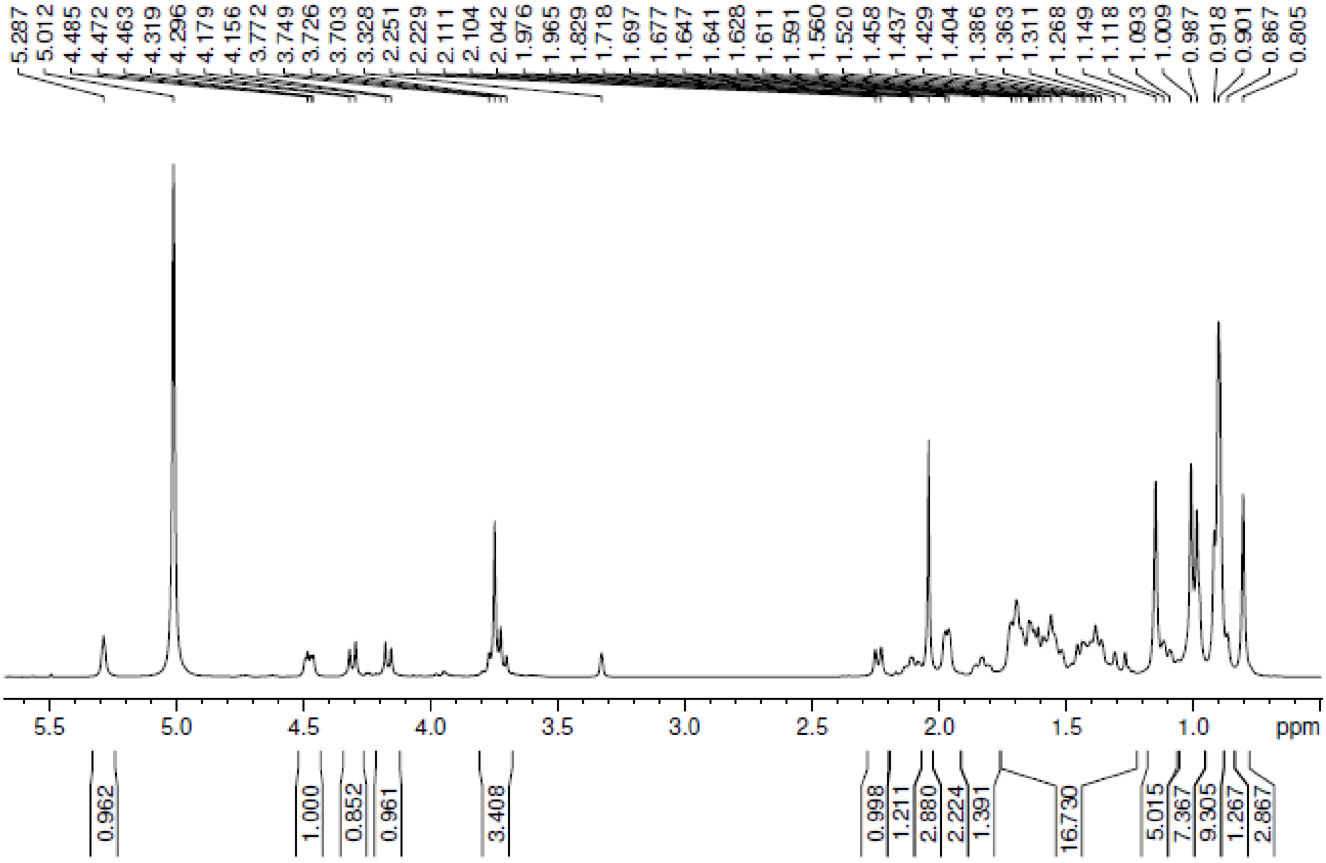

3β-[2-guanidine-3-hydroxy-2-(hydroxymethyl)propyl]-3-O-acetyl-urs-12-en-28-oate trifluoroacetate (18b). ^{13}C NMR spectra (MeOD)

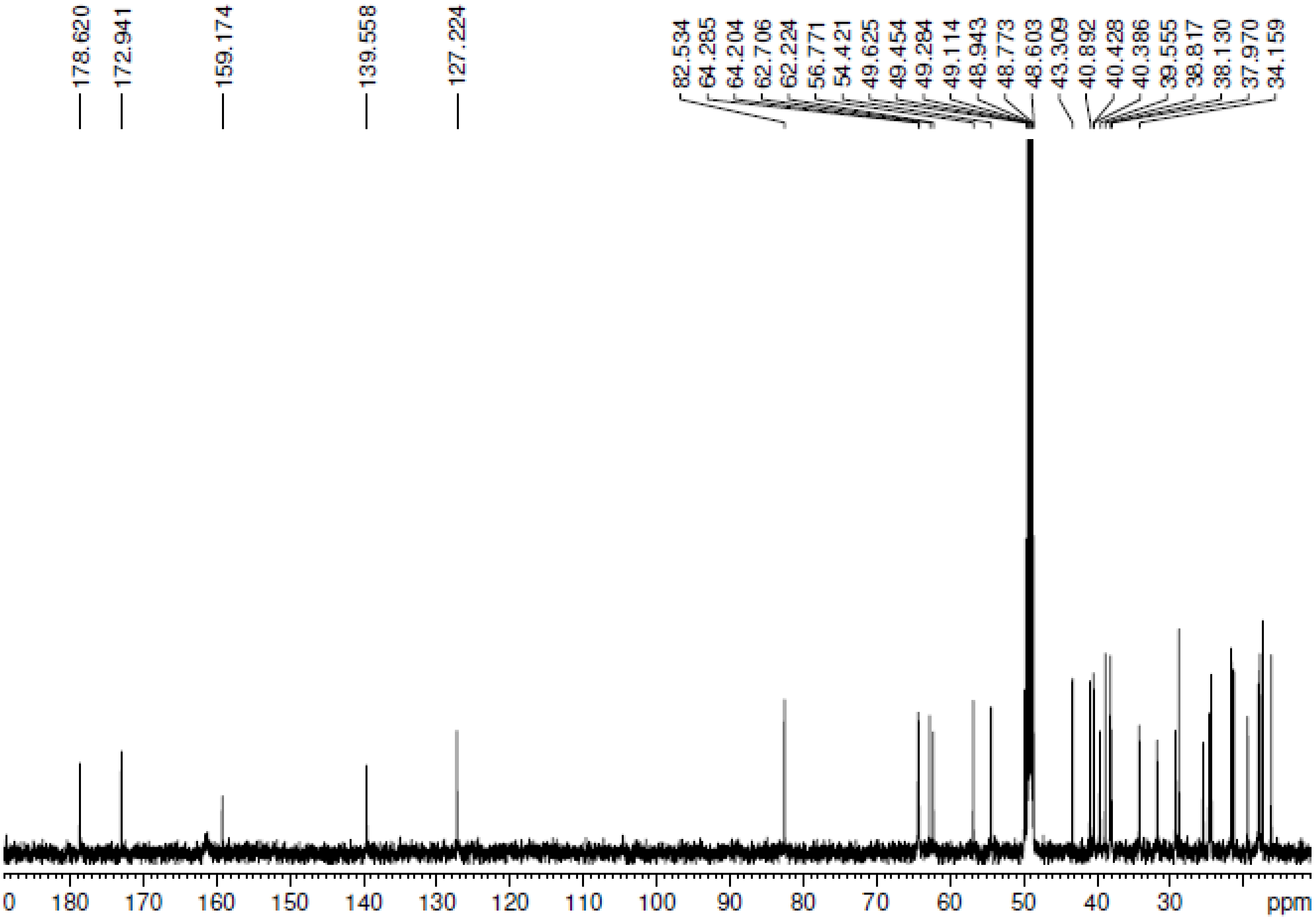

3β-[2-guanidine-3-hydroxy-2-(hydroxymethyl)propyl]-3-O-acetyl-urs-12-en-28-oate trifluoroacetate (18b). ^{19}F NMR spectra (MeOD)

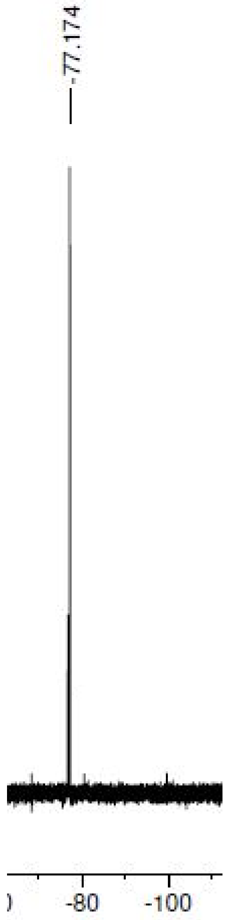

3β-[2-guanidine-3-hydroxy-2-(hydroxymethyl)propyl]-3-O-acetyl-olean-12-en-28-oate trifluoroacetate (20b). ^{1}H NMR spectra (MeOD)

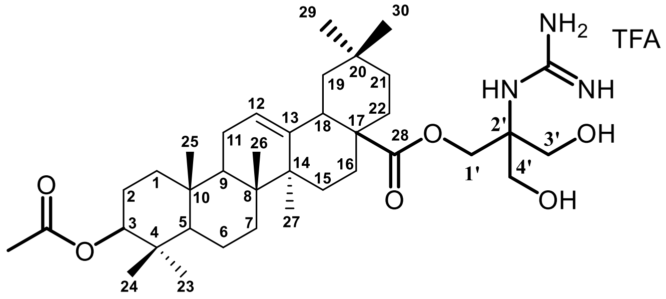

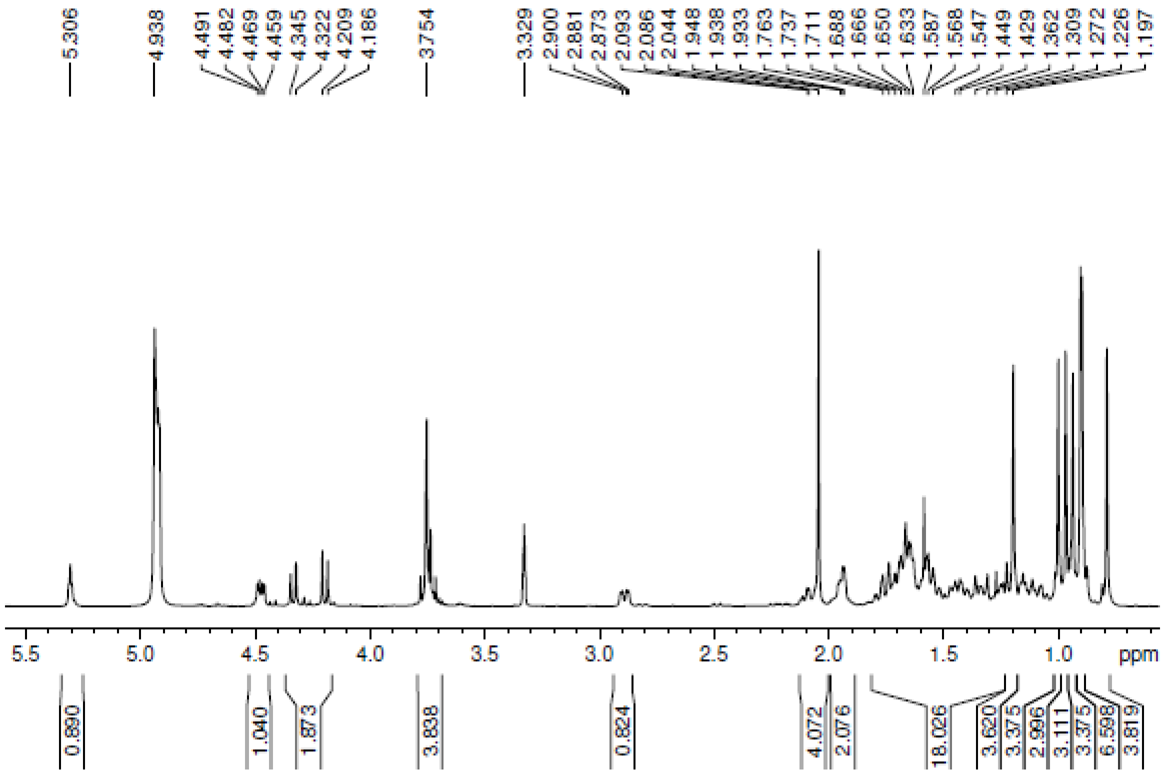

3β-[2-guanidine-3-hydroxy-2-(hydroxymethyl)propyl]-3-O-acetyl-olean-12-en-28-oate trifluoroacetate (20b). ^{13}C NMR spectra (MeOD)

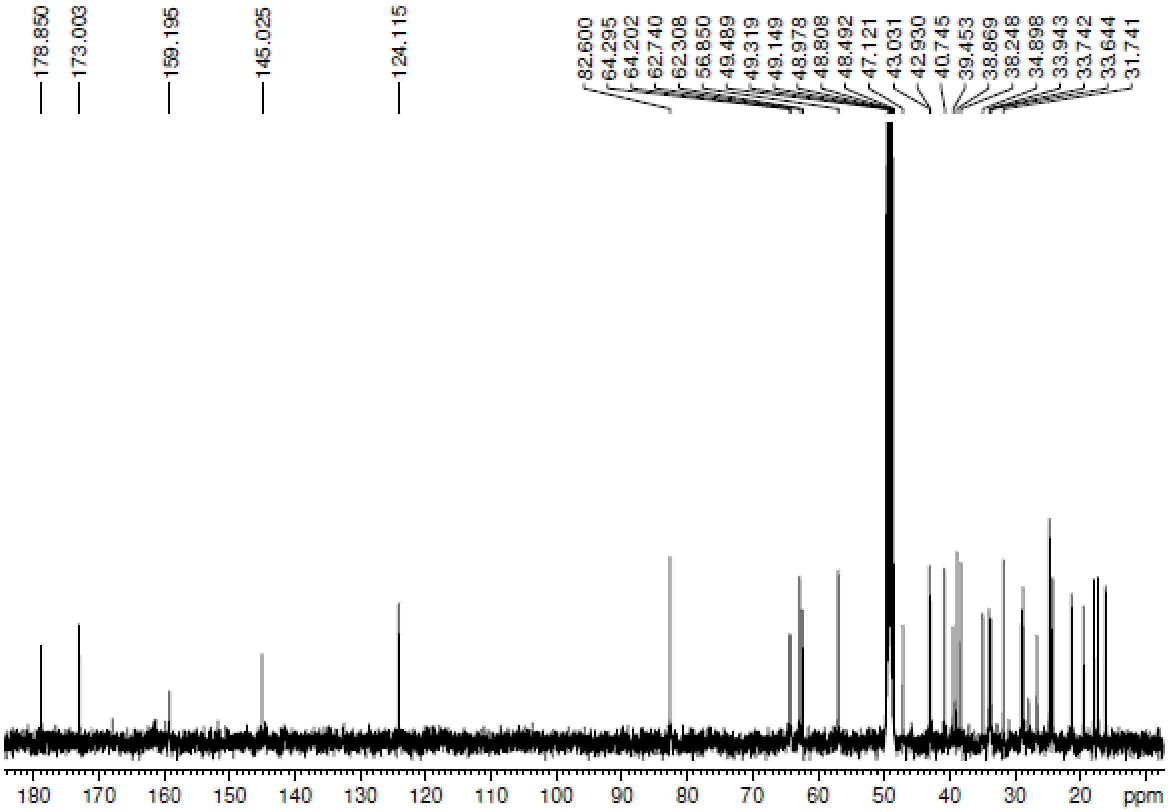

3β-[2-guanidine-3-hydroxy-2-(hydroxymethyl)propyl]-3-O-acetyl- olean -12-en-28-oate trifluoroacetate (20b). ^{19}F NMR spectra (MeOD)

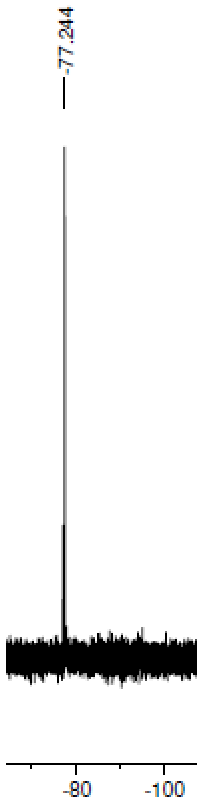

N-(4-buthylgyanidine)-3-oxolupane-28-amide dihydrochloride (9b).

^{1}H NMR spectra (d$_6$-DMSO)

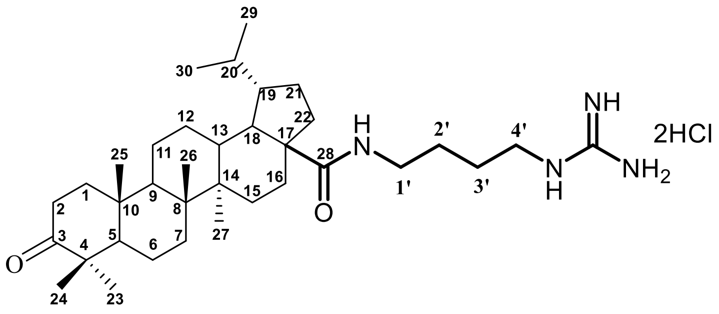

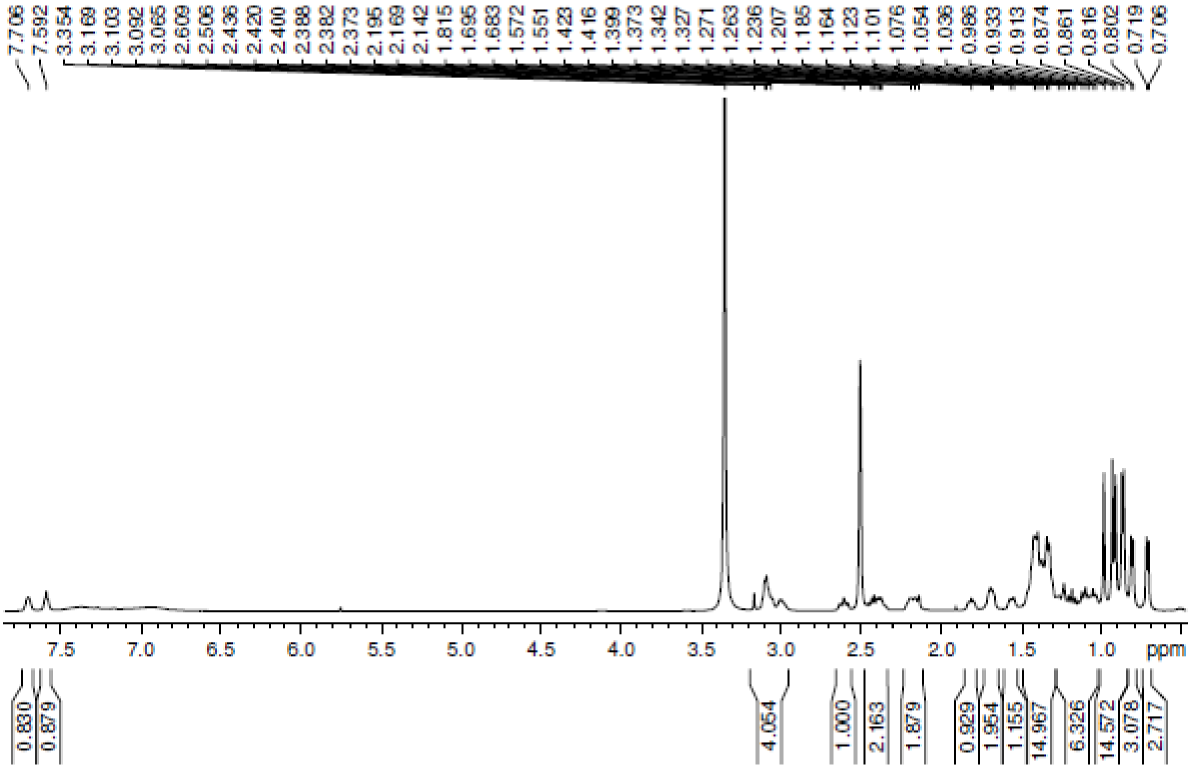

N-(4-buthylgyanidine)-3-oxolupane-28-amide dihydrochloride (9b).

^{13}C NMR spectra (d_6-DMSO)

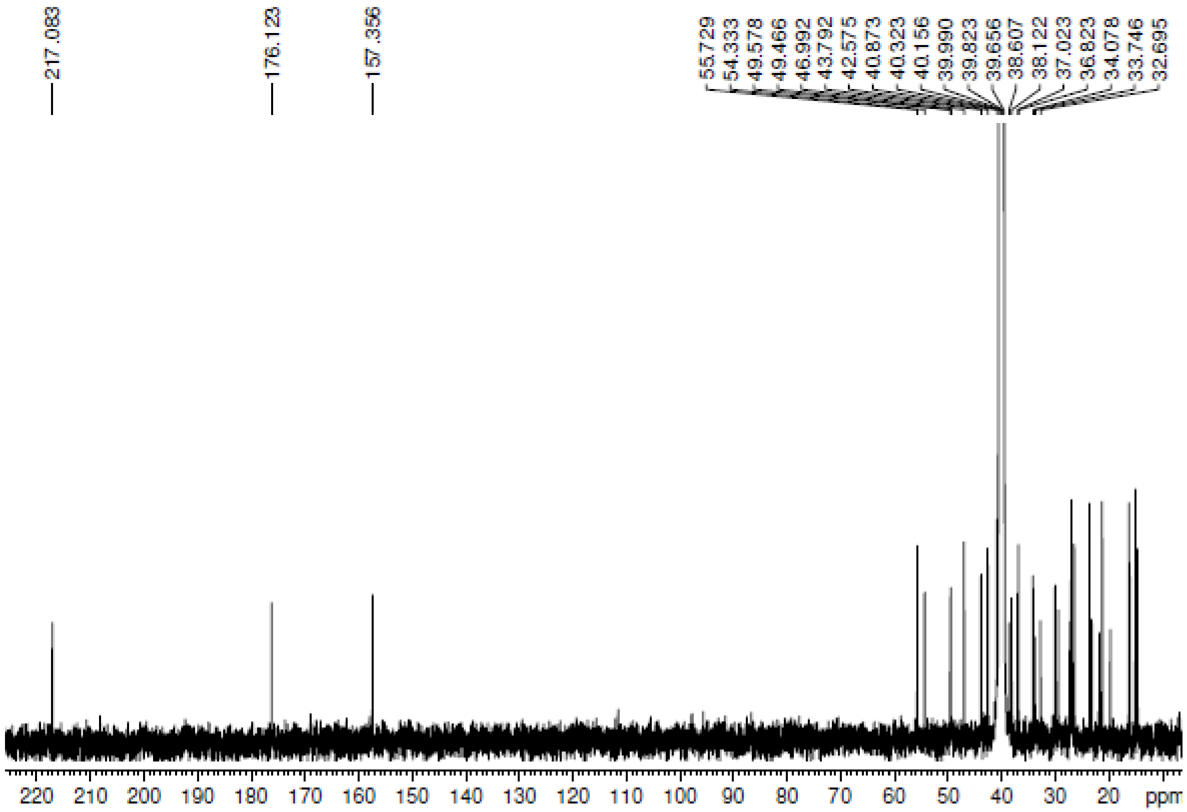

3β-N-(2-ethylgyanidine)-3-O-acetyl-lupane-28-amide hydrochloride (10b).

^{1}H NMR spectra (MeOD)

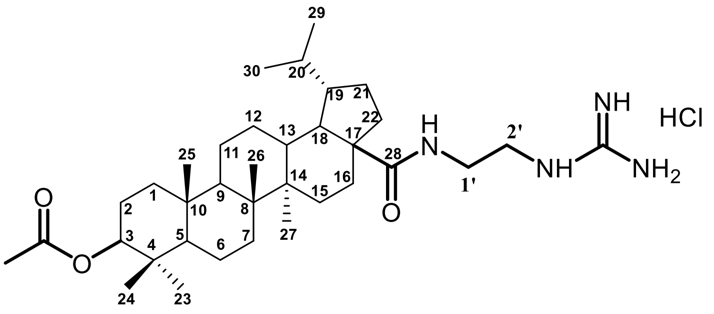

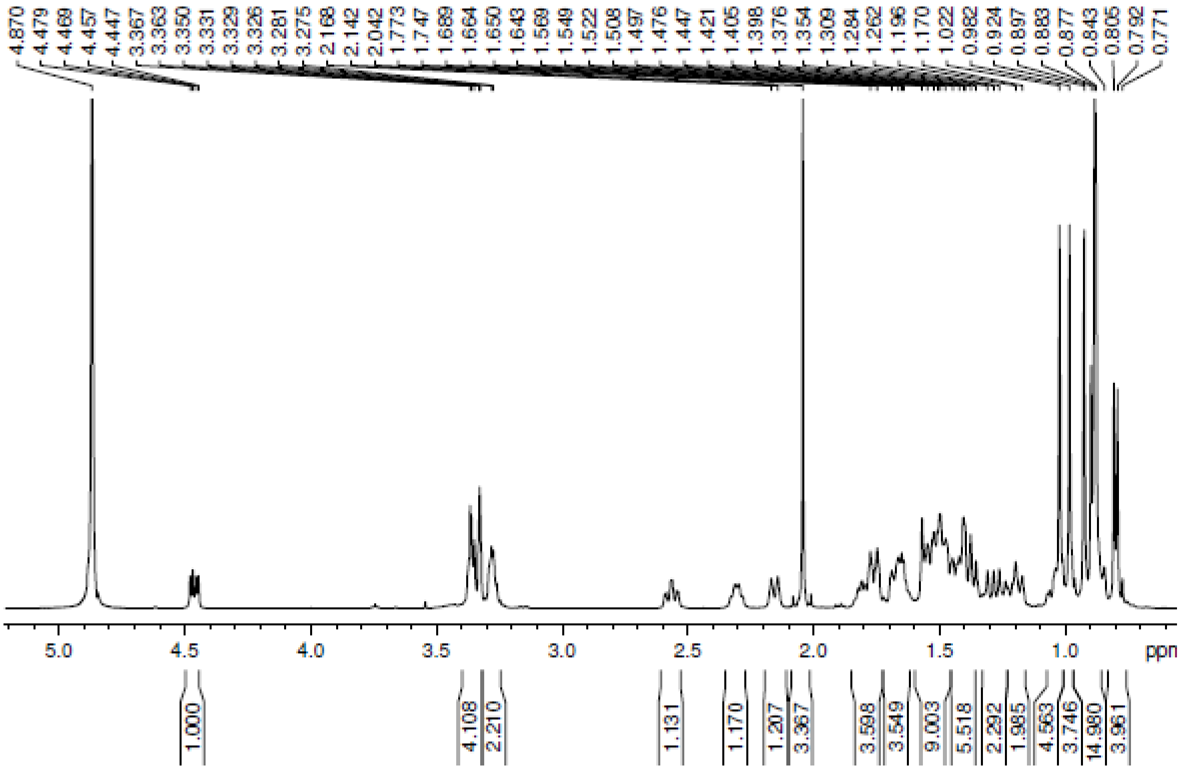

3β-N-(2-ethylgyanidine)-3-O-acetyl-lupane-28-amide hydrochloride (10b).

^{1}H NMR spectra (MeOD)

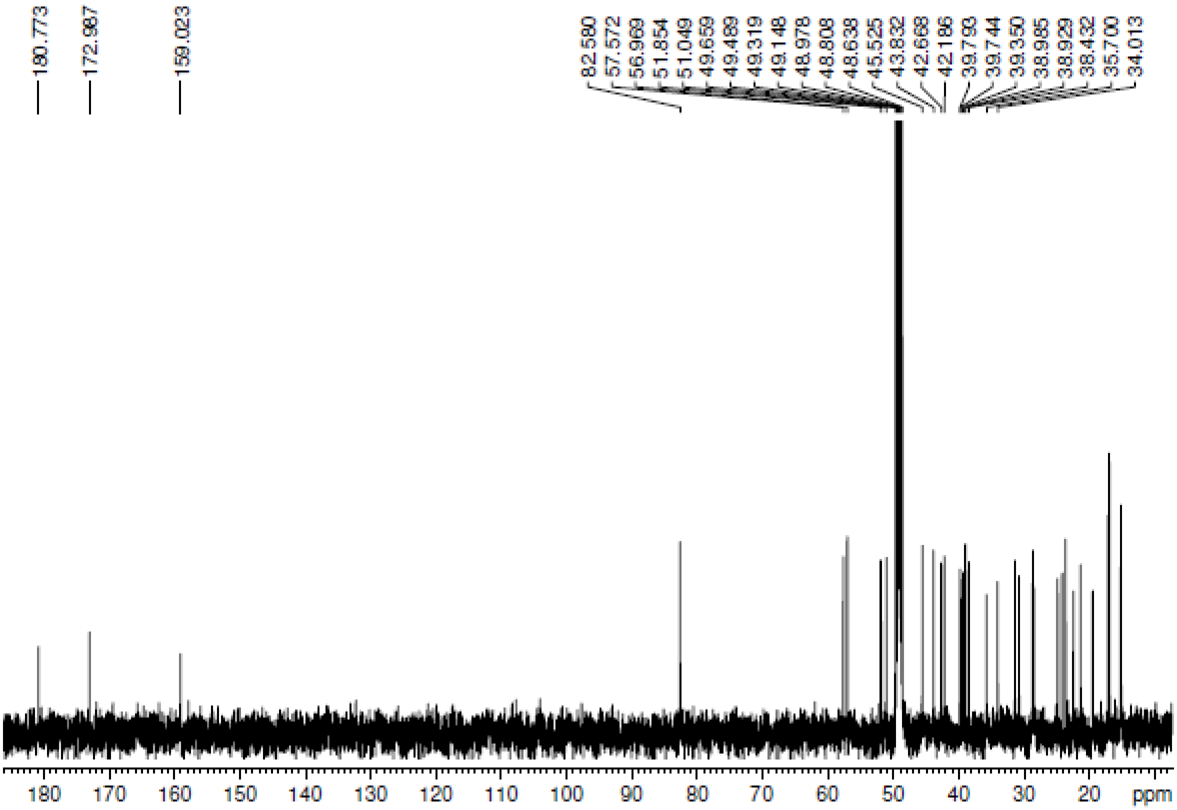

3β-N-(4-buthylgyanidine)-3-O-acetyl-lupane-28-amide hydrochloride (11b).

^{1}H NMR spectra (MeOD)

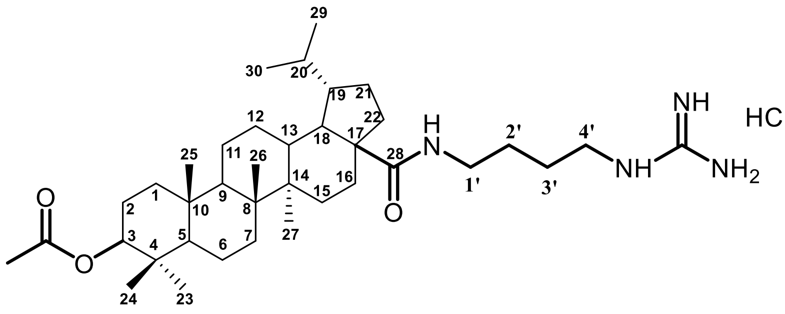

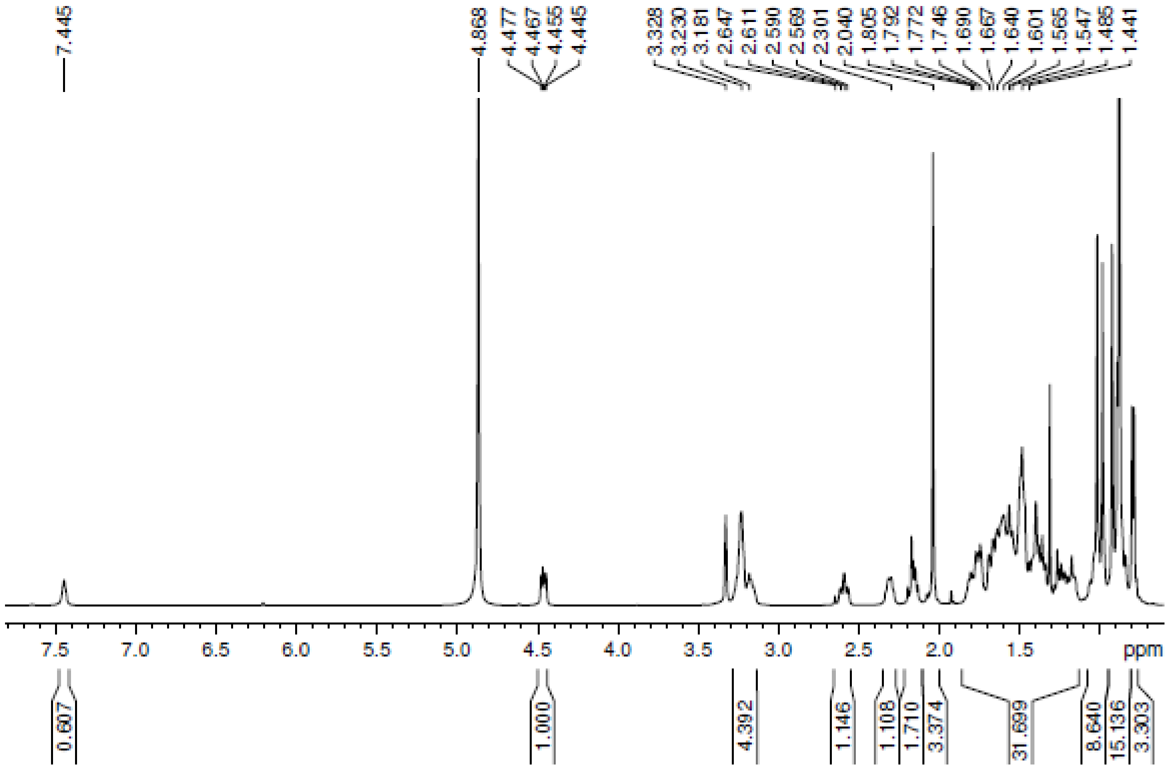

3β-N-(4-buthylgyanidine)-3-O-acetyl-lupane-28-amide hydrochloride (11b).

^{13}C NMR spectra (MeOD)

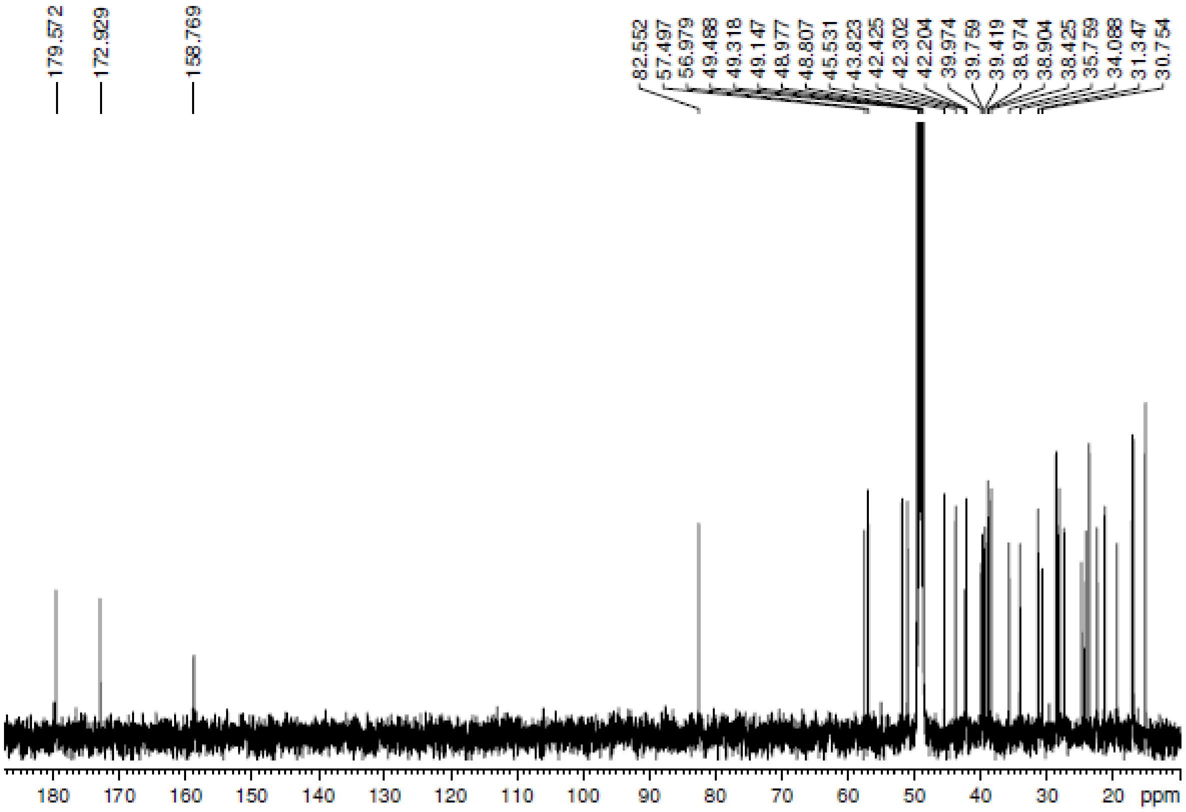

3β-N-[2-(N,N’-bis-ethylgyanidine)-ethyl]-3-O-acetyl-lupane-28-amide dihydrochloride (12b).

^{1}H NMR spectra (MeOD)

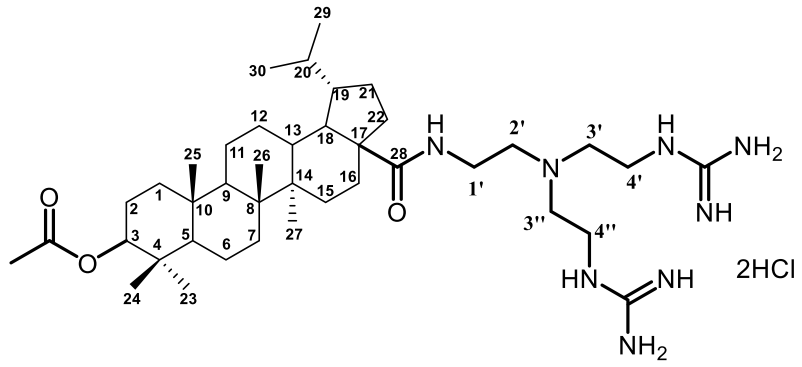

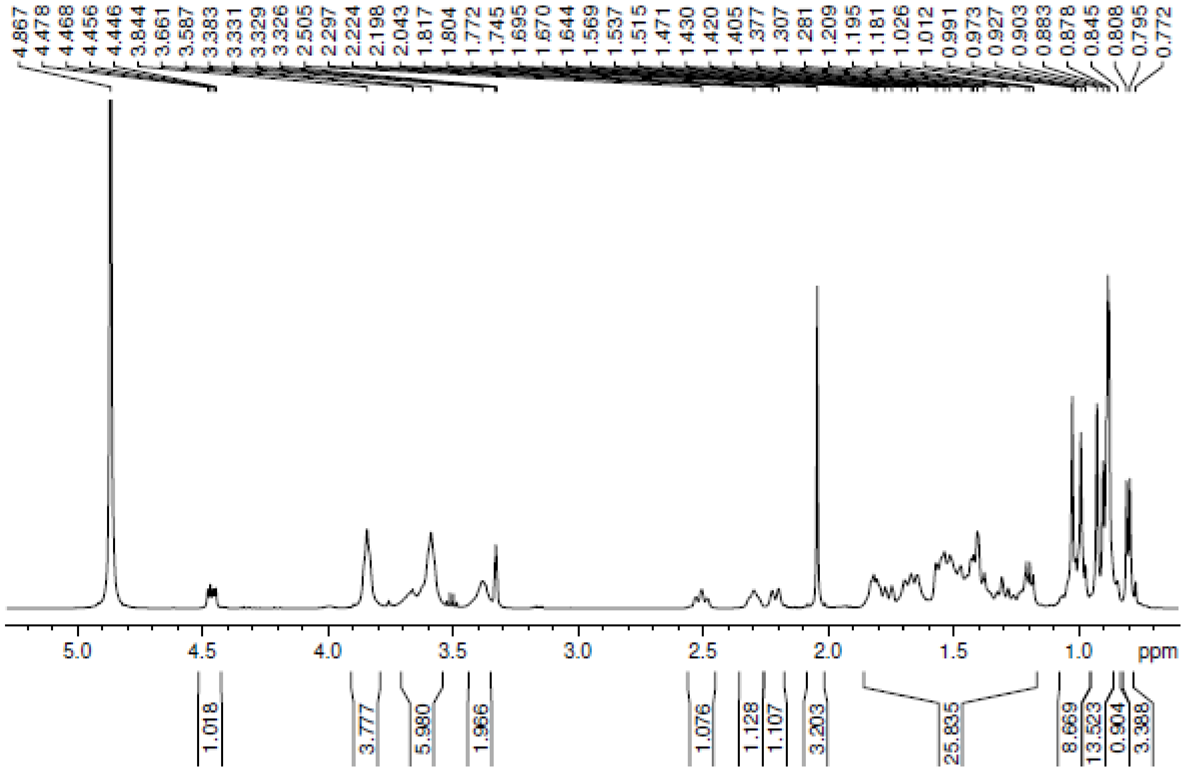

3β-N-[2-(N,N'-bis-ethylgyanidine)-ethyl]-3-O-acetyl-lupane-28-amide dihydrochloride (12b).

^{13}C NMR spectra (MeOD)

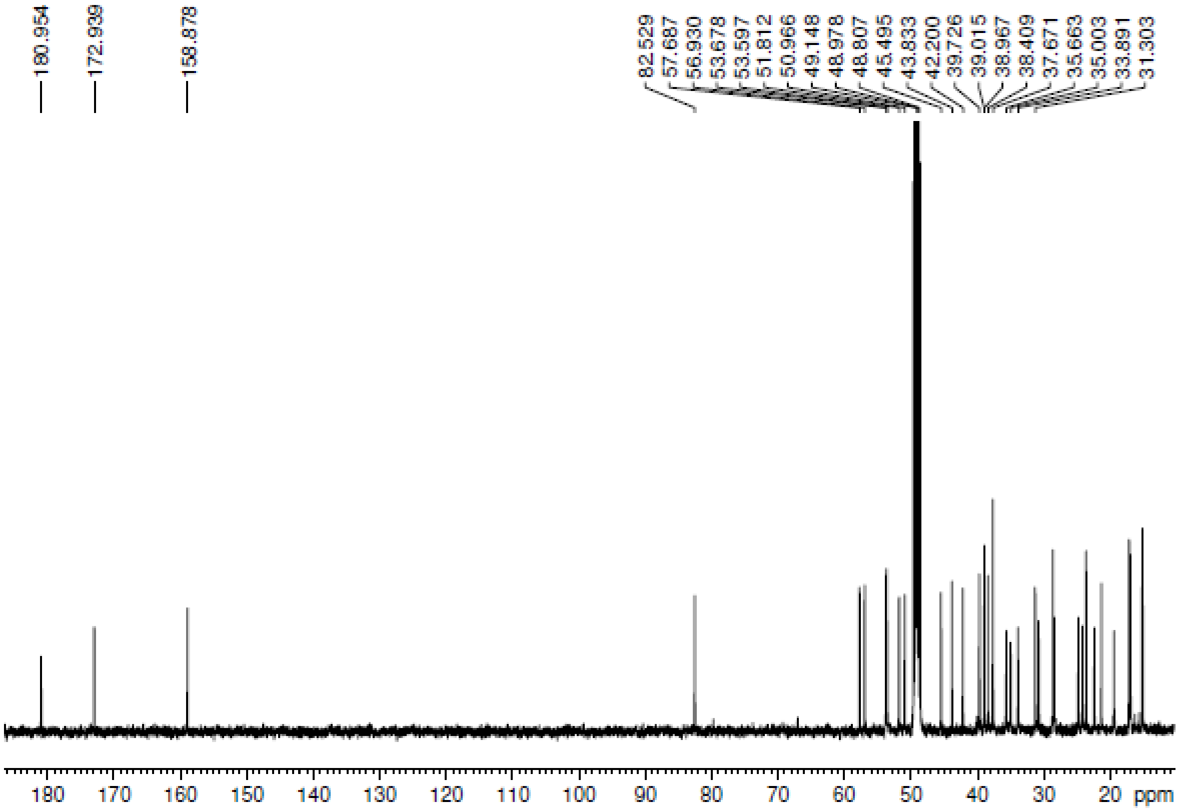

3β-[2-guanidine-3-hydroxy-2-(hydroxymethyl)propyl]-3-O-acetyl-lupane-28-oate hydrochloride (15c).^{1}H NMR spectra (MeOD)

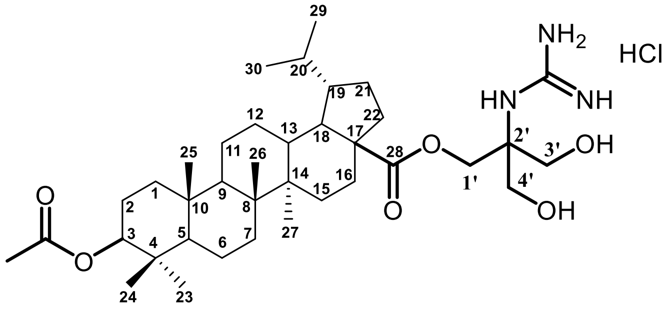

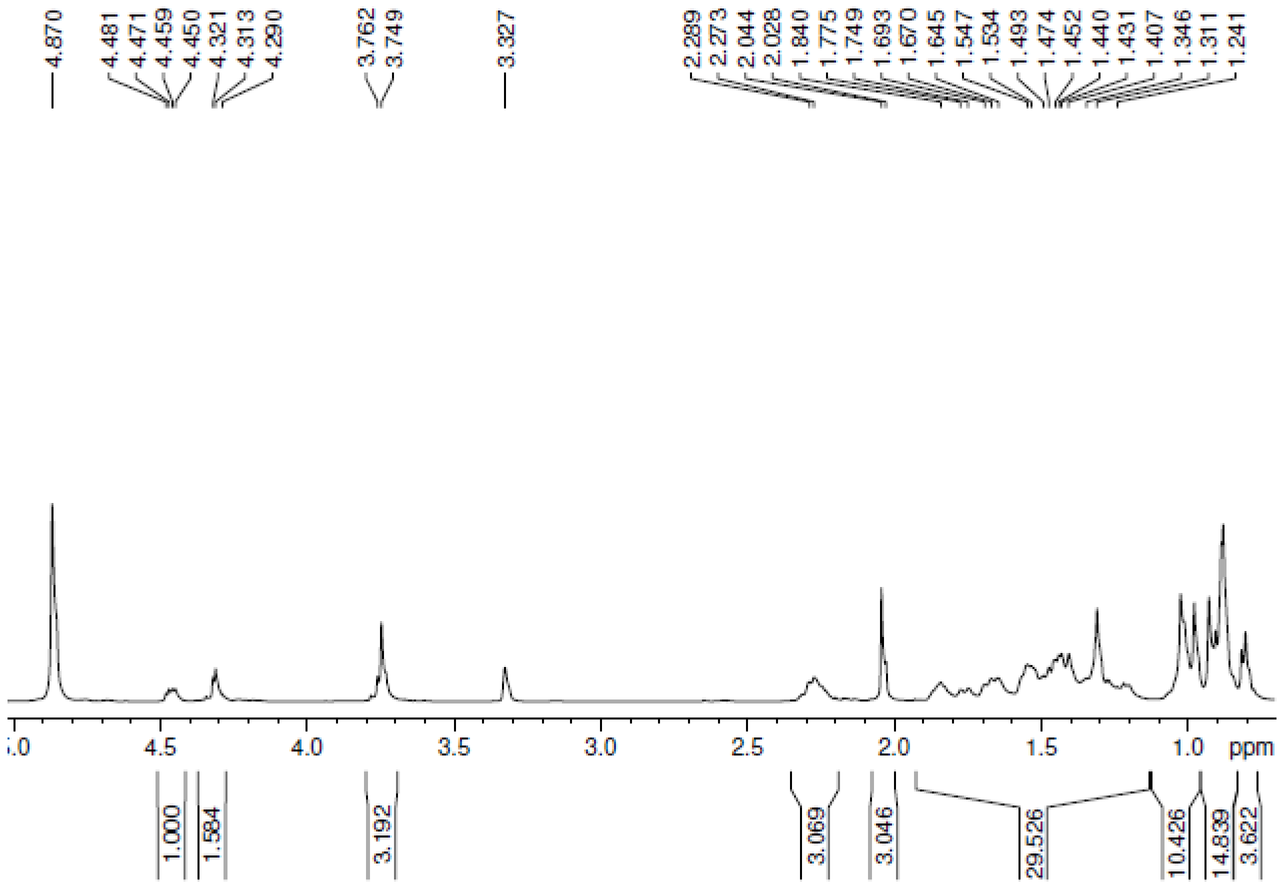

3β-[2-guanidine-3-hydroxy-2-(hydroxymethyl)propyl]-3-O-acetyl-lupane-28-oate hydrochloride (15c).^{13}C NMR spectra (MeOD)

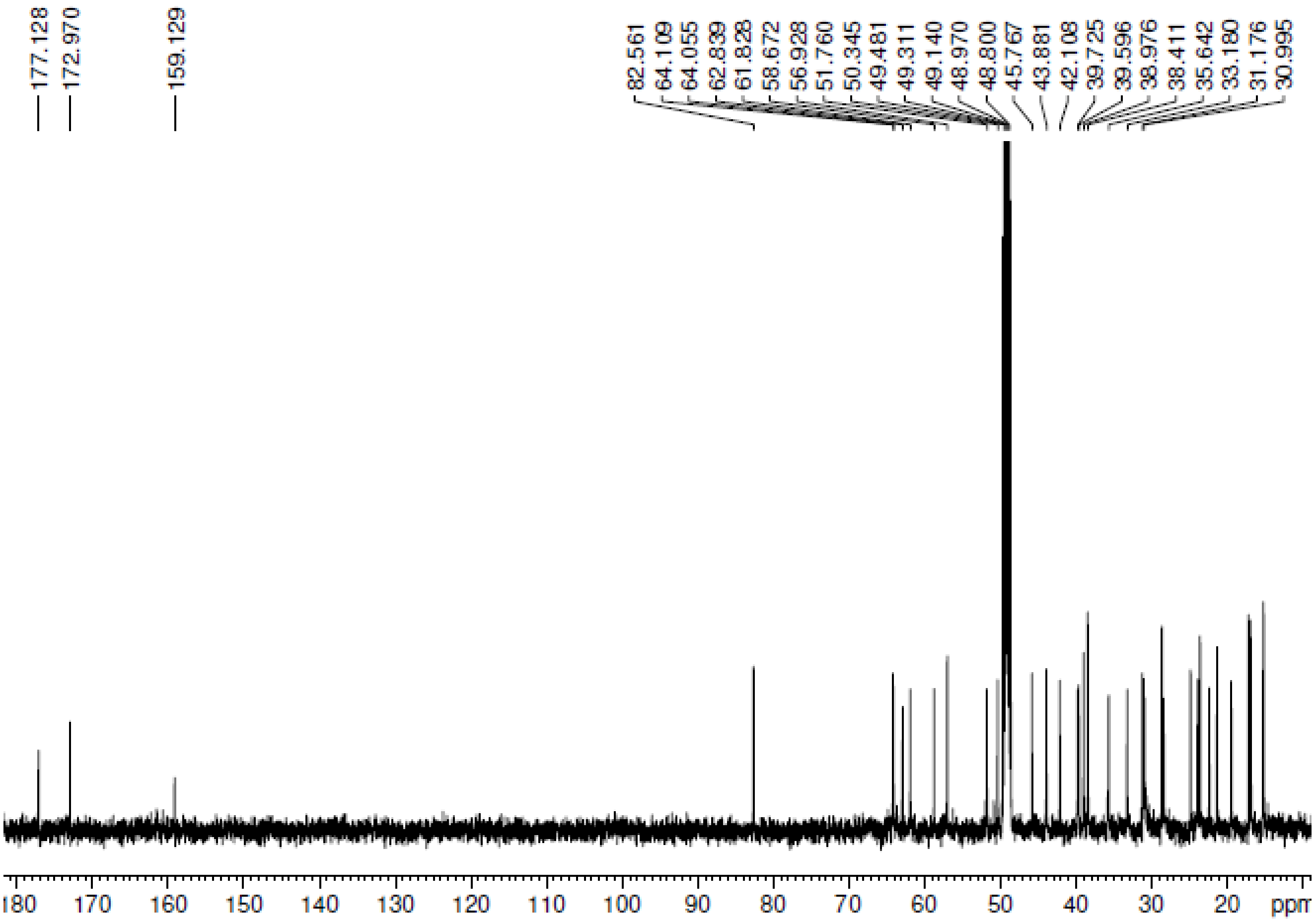

3β-[2-guanidine-3-hydroxy-2-(hydroxymethyl)propyl]-3-O-acetyl-urs-12-en-28-oate hydrochloride (18c). ^{1}H NMR spectra (MeOD)

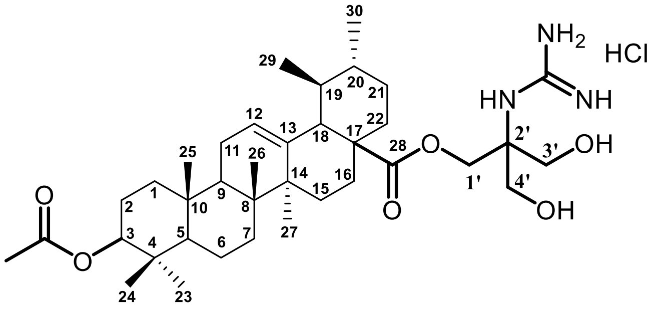

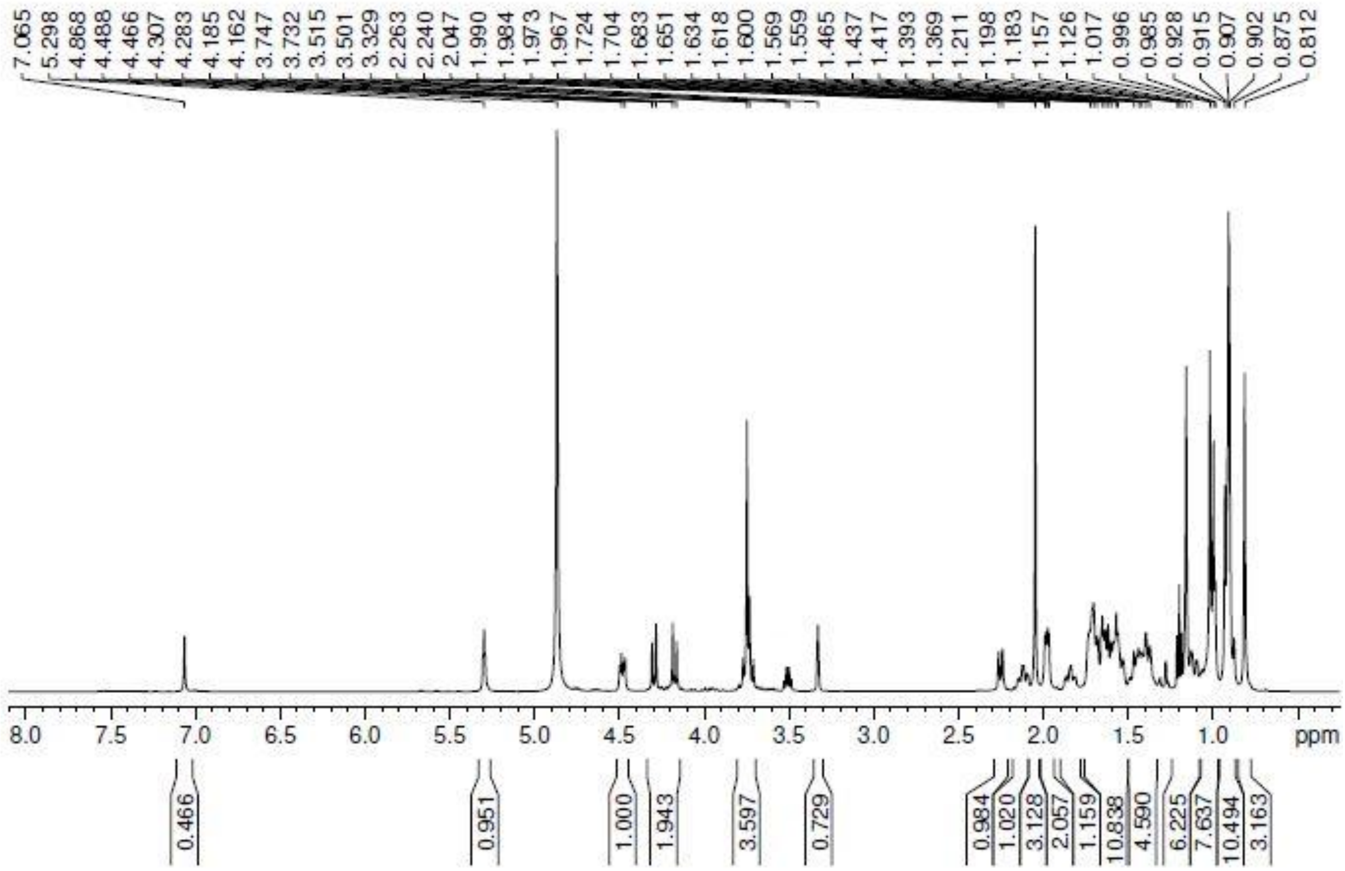

3β-[2-guanidine-3-hydroxy-2-(hydroxymethyl)propyl]-3-O-acetyl-urs-12-en-28-oate hydrochloride (18c). ^{13}C NMR spectra (MeOD)

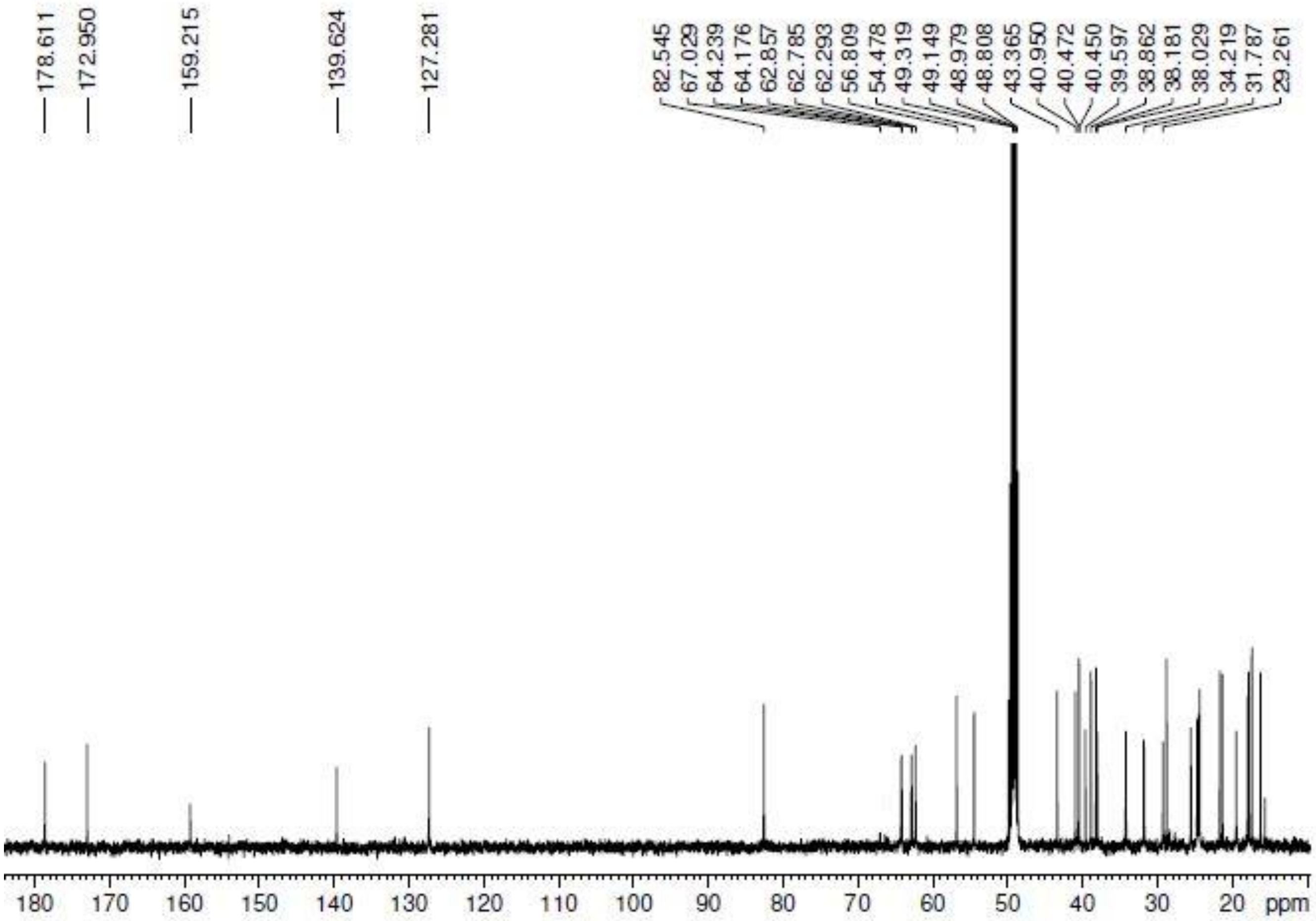

3β-[2-guanidine-3-hydroxy-2-(hydroxymethyl)propyl]-3-O-acetyl-olean-12-en-28-oate hydrochloride (20c). ^{1}H NMR spectra (MeOD)

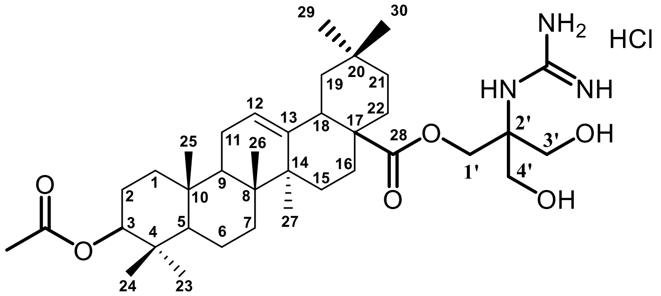

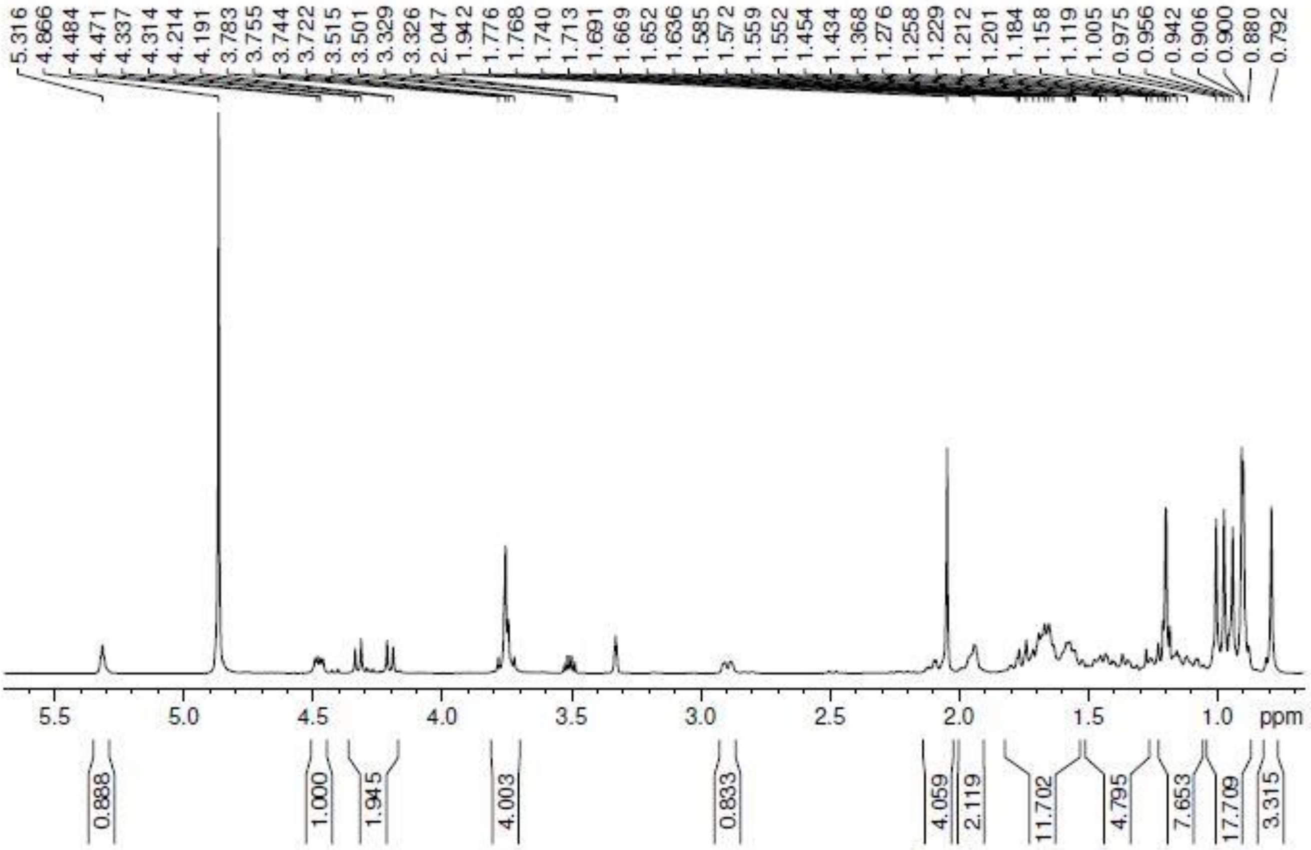

3β-[2-guanidine-3-hydroxy-2-(hydroxymethyl)propyl]-3-O-acetyl-olean-12-en-28-oate hydrochloride (20c). ^{13}C NMR spectra (MeOD)

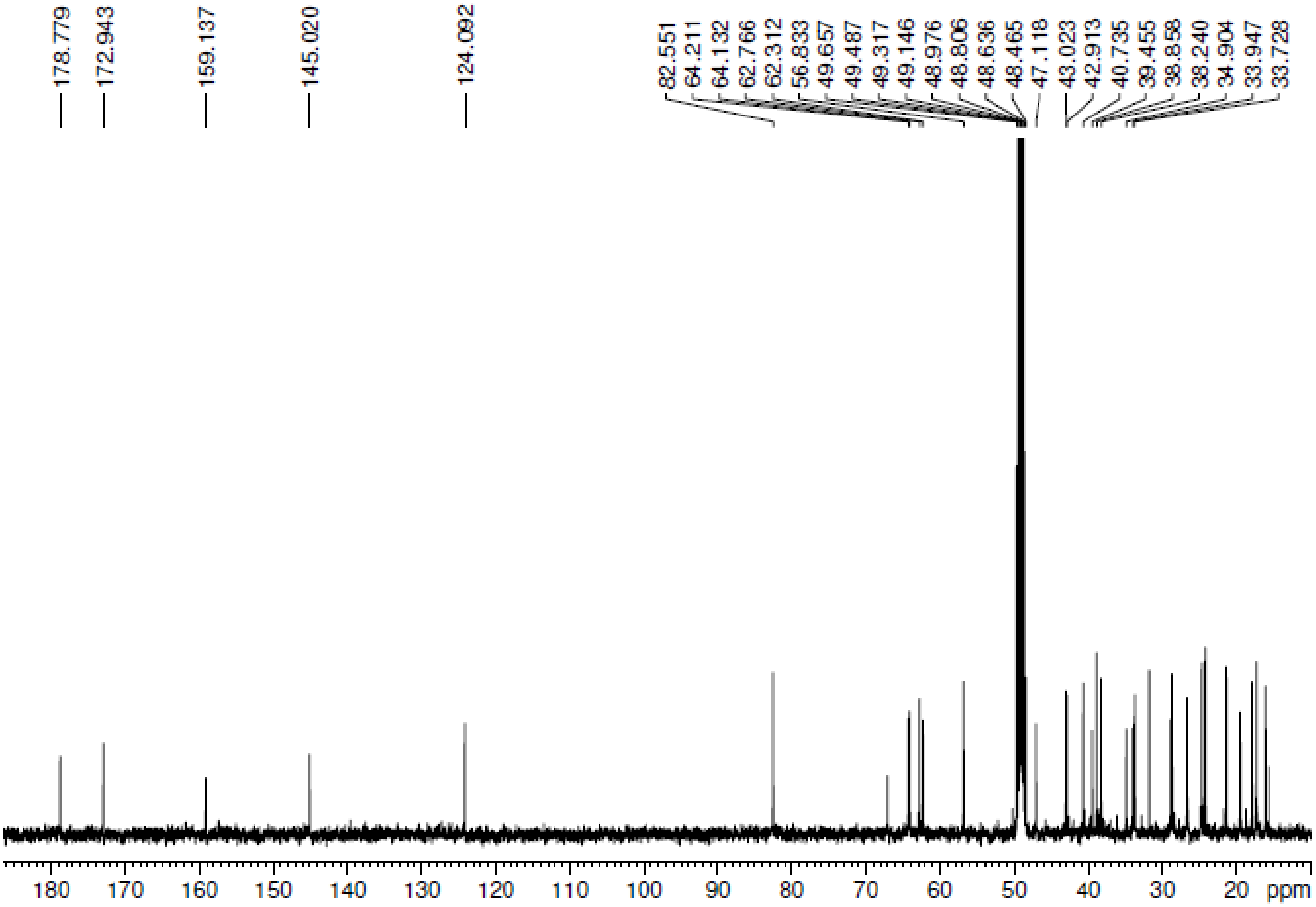

3β-N-(4-buthylgyanidine)-3-hydroxy-lupane-28-amide (14).

^{1}H NMR spectra (d$_6$-DMSO)

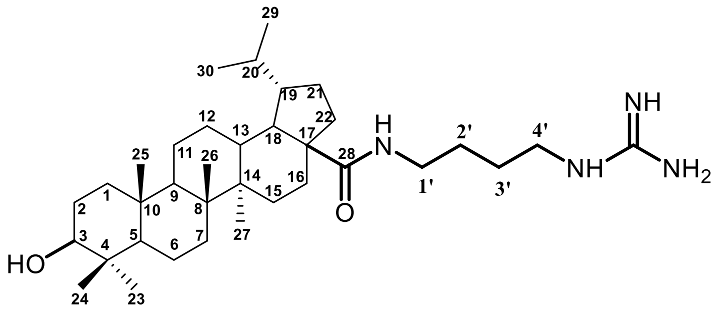

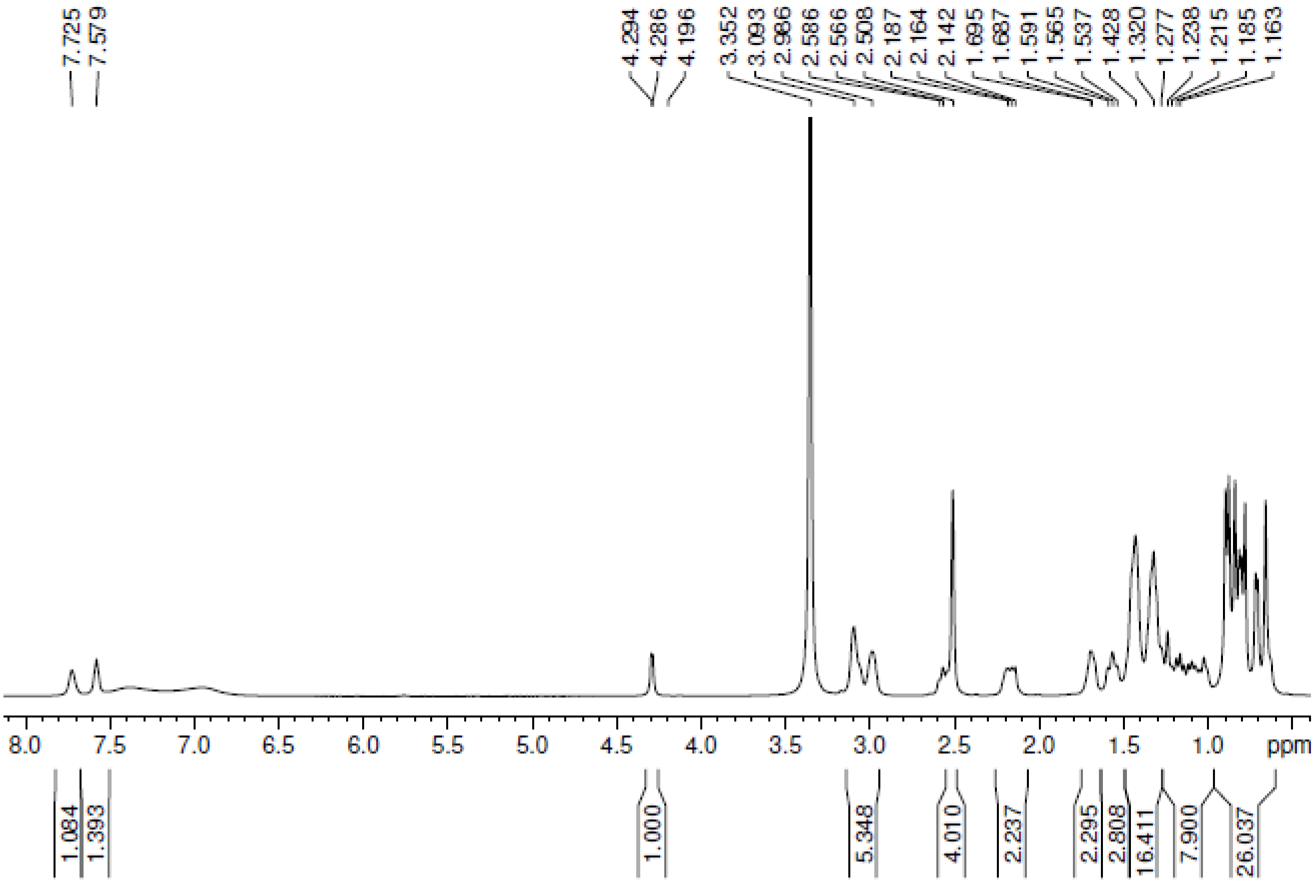

3β-N-(4-buthylgyanidine)-3-hydroxy-lupane-28-amide (14).

^{13}C NMR spectra (d_6-DMSO)

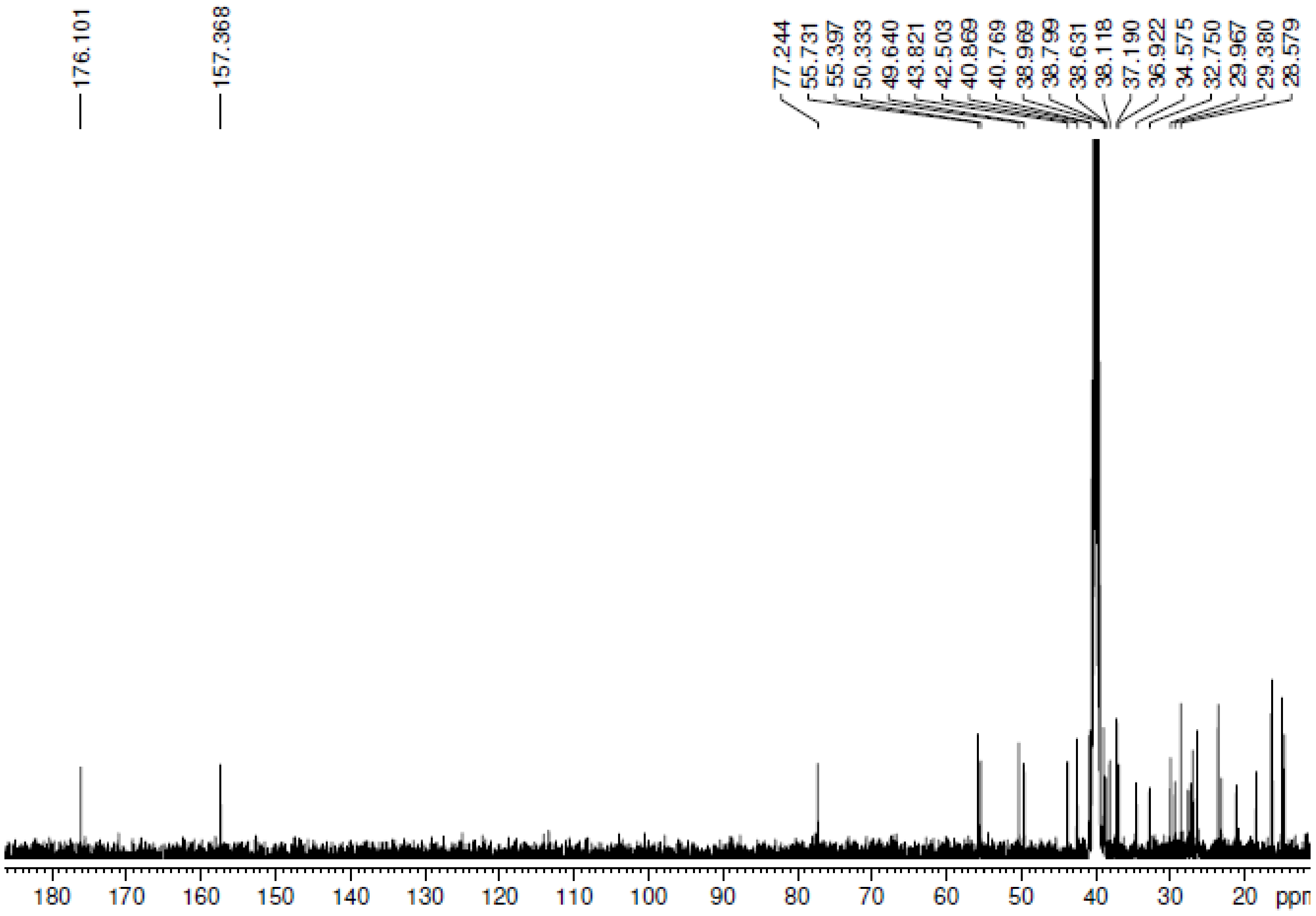

Supplement: Supplementary file 1 [file molecules-23-03000-s001.pdf]
